# Gender bias in cultural tightness across the 50 US states, its correlates, and links to gender inequality in leadership and innovation

**DOI:** 10.1093/pnasnexus/pgad238

**Published:** 2023-07-20

**Authors:** Xin Qin, Roy Y J Chua, Ling Tan, Wanlu Li, Chen Chen

**Affiliations:** Sun Yat-sen Business School, Sun Yat-sen University, No. 135 Xinggang West Road, Guangzhou, 510275, China; Lee Kong Chian School of Business, Singapore Management University, 50 Stamford Road #5064, Singapore, 178899, Singapore; School of Management, Guangdong University of Technology, No. 161 Yinglong Road, Guangzhou, 510520, China; Sun Yat-sen Business School, Sun Yat-sen University, No. 135 Xinggang West Road, Guangzhou, 510275, China; Sun Yat-sen Business School, Sun Yat-sen University, No. 135 Xinggang West Road, Guangzhou, 510275, China

**Keywords:** cultural tightness–looseness, gender inequality, gender bias, leadership, innovation, United States

## Abstract

Cultural tightness theory, which holds that “tight” cultures have rigid norms and sanctions, provides unique insights into cultural variations. However, current theorizing has not analyzed gender differences in cultural tightness. Addressing this gap, this research shows that women are more constrained than men by norms within the same society. By recruiting 15,425 respondents, we mapped state-level gender bias in cultural tightness across the United States. Variability in gender bias in cultural tightness was associated with state-level sociopolitical factors (religion and political ideology) and gender-related threats. Gender bias in cultural tightness was positively associated with state-level gender inequality in (business and political) leadership and innovation, two major challenges faced by women professionals. Overall, this research advances cultural tightness theory and offers a cultural norms account on persistent gender inequalities in society.

**Significance Statement**

Cultural tightness theory, which holds that “tight” cultures have rigid norms and sanctions, provides unique insights into cultural variations. However, current theorizing has not analyzed gender differences in cultural tightness. We propose that gender bias in cultural tightness likely exists and it varies across different societies and regions. Specifically, we mapped state-level gender bias in cultural tightness across the 50 US states. We found that such variability was associated with state-level sociopolitical factors (religion and political ideology) and gender-related threats. Importantly, gender bias in cultural tightness was positively associated with state-level gender inequality in (business and political) leadership and innovation, two major challenges faced by women professionals in the modern society. Taken together, these findings advance cultural tightness–looseness theory by injecting an important gender dimension and offer a cultural norms account on persistent gender inequalities.

Cultural psychologists have used cultural “tightness” and “looseness” to describe different cultures: tight cultures have “strong norms and a low tolerance of deviant behavior,” whereas loose cultures have “weak norms and a high tolerance of deviant behavior” (1). This stream of research provides unique insights toward understanding cultural variations across societies (2–5). However, current theorizing has not analyzed gender differences in cultural tightness. In the current research, we propose that societal-level gender bias in cultural tightness likely exists and that it varies across different societies and regions. Furthermore, we theorize that societal-level gender bias in cultural tightness is associated with societal-level gender inequality in leadership and innovation. We focus on gender inequality in leadership and innovation, as a variety of research and broader statistics have shown that women professionals are starkly underrepresented in elite leadership (including business and political leadership) and fields that involve innovation (6–12). Moreover, gender inequality in leadership and innovation is integrally relevant to cultural tightness theory because both effective leadership and innovation involve revising extant norms and challenging the status quo (13, 14).

There are two theoretical premises for this gender bias at the societal level. First, in most societies, women often face and need to comply with stronger social norms (especially gender stereotypical norms) compared with men (15). For example, societies regard women who choose their career over having children as “selfish”; in contrast, men are unlikely to face such a judgment (16, 17). Similarly, societies see women's engagement in premarital sexual behaviors as “wrong,” whereas it is somehow allowed—or even right—for men (18, 19). Although precarious manhood theory posits that men may be more constrained by certain norms related to protecting their manhood status (e.g. men are expected to exhibit greater agency and dominance in social settings to demonstrate their masculinity) (20, 21), these greater constraints on men pertain to only highly specific domains. Women, on the other hand, face more constraints than men over a wide range of domains. That is, women face overall more constraints than men in societies (15, 19), even though the specific constraints they face are sometimes different (20, 21). Second, women often receive harsher punishments when they deviate from social norms and expectations than men (22, 23). For example, a recent study found that after committing misconduct in the financial advisory industry, female advisers were 20% more likely to lose their jobs and 30% less likely to find new jobs compared with male advisers (23). Thus, we hypothesize that societal-level gender bias in cultural tightness exists—societal-level norms regarding permissible behaviors and tolerance of aberrant behaviors do not apply equally to men and women.

Such gender bias in cultural tightness likely varies across societies. For instance, one study shows that societies with different subsistence economies not only have quite different levels of strictness in child-rearing norms and practices (e.g. obedience training and responsibility training) but also have different norms and practices for raising girls versus boys (24). Specifically, in subsistence economies that rely primarily on agriculture or animal husbandry to provide basic needs, girls are raised to participate in tasks that call for more continuous responsibility, adherence to routines, and obedience (e.g. childbearing and maintaining the good health of the herd) than do the type of tasks that boys are raised for (e.g. decision-making on crop cultivation and animal raising). Accordingly, responsibility, adherence to routines, and obedience are emphasized more strongly in the training of girls than boys (24). Also, sociologists have found that collectivistic societies such as India, Japan, and Kuwait usually have higher levels of hierarchical power structures and have more conservative ideologies (e.g. endorsement of men's domination over women) than individualistic societies such as the United States (25). As such, collectivistic societies may have stronger norms regarding permissible behaviors for women compared with men. In sum, we hypothesize that societies may impose different norms and rules on the two sexes and have different levels of tolerance toward aberrant behaviors for women compared with men. Accordingly, as with most culture-related constructs, gender bias in cultural tightness is a societal-level construct rather than an individual-level construct. Higher societal-level gender bias in cultural tightness means women (compared with men) face overall stronger norms regarding permissible behaviors and intolerance of aberrant behaviors in a given society.

Building upon Gelfand et al.'s (2011) systems framework of cultural tightness (1), we posit that sociopolitical factors (religion and political ideology) and gender-related threats are associated with gender biases that manifest themselves in a society's norms and its tolerance for aberrant behaviors. It is worth noting that these categories of factors are not mutually exclusive (e.g. political ideology might implicate religion and religious doctrines might pose different threats to men and women). We organized the various factors into these two main categories so as to structure the discussion and analyses for greater readability.

First, with regard to sociopolitical factors, research from a variety of disciplinary perspectives has documented that religions often play significant roles in structuring gender norms (26, 27). Typically, the most conservative religions advocate patriarchal gender roles and exclude women from positions of leadership (28), often dictating the “proper” roles for women at home and the control of women's sexuality (29, 30). For example, religious doctrine in the common Christian denominations in the United States overwhelmingly characterizes authority—both human and divine—in masculine terms (31). Conservative Protestant groups are more likely to hold highly traditional views of societal roles for men and women and often idealize women's traditional roles (32, 33). More specifically, evangelical Protestant churches in the United States often embrace home schooling, which can discourage women from reentering the workplace as their children grow. In addition, both evangelical Protestant churches and the Roman Catholic Church preach that abortion and birth control are sinful—beliefs that could also constrain women and restrict them to their traditional gender roles. Also, the Mormon church spelled out its philosophy on gender roles in its 1996 treatise “The Family: A Proclamation to the World,” which indicated: “Mothers are primarily responsible for the nurture of their children.” Thus, religions contribute to conservative beliefs that advocate patriarchal gender roles (28), which are associated with greater constraints on women and hence greater gender bias in cultural tightness.

Furthermore, some political factors (e.g. political conservatism) are linked to traditional gender role beliefs and patriarchal views, which are in turn positively associated with gender bias in cultural tightness. When politically conservative values are dominant in society, people are more likely to seek to defend the institutions and social values of the existing order, including traditional gender roles (34). Specifically, those more conservative in ideology are often more dogmatic, desiring order and certainty, believing more strongly in group hierarchies and traditional societal roles. Accordingly, conservatives tend to endorse a relatively binary view of gender (e.g. the way that men and women ought to look and behave), which helps them rationalize or legitimize a traditional gender-based hierarchy (35). These endorsements of traditional gender role beliefs and patriarchal values would be positively associated with gender bias in cultural tightness.

Second, the experience of threats has been theorized as a key driver of cultural tightness (1, 2, 4). Gender-related social threats (e.g. sexism) and physical threats (e.g. sexual violence, domestic violence, and human trafficking) could vary across societies and therefore differentially constrain women. Sexism is representative of social threats that women might face. Gender studies have identified two forms of sexism in societies: benevolent sexism and hostile sexism (34, 36). In patriarchal societies, benevolent sexism fosters “protective attitudes toward women, a reverence for the role of women as wives and mothers, and an idealization of women as romantic love objects” (36). Men believe that women are vulnerable and need more protection and in turn place more constraints on women to protect them from harm. Conversely, hostile sexism refers to an ideology that characterizes “women as incompetent, overly emotional and attempting to manipulate men to gain power” (37). Those who endorse hostile sexism have derogatory attitudes toward women who challenge men's power (36, 37) and engage in more aggressive behavior toward their partners (38–40). Overall, although benevolent sexism and hostile sexism have different premises and views about women, they share a common assumption that “women inhabit restricted domestic roles and are the ‘weak’ sex (36)” and serve to justify men's power, control, and dominance.

Additionally, women (compared with men) may face more physical threats, ranging from robbery, domestic violence, sexual harassment, to human trafficking (41, 42). While men and boys also face these threats, the majority of individuals identified as victims in violence cases and identified as trafficked for both labor and commercial sex are women and girls. The Global Report on Trafficking in Persons in 2020 found that 84% victims of human trafficking among three countries in North America (i.e. Canada, Mexico, and the United States) were women and girls (43). Accordingly, to protect women from such threats and the associated harm, some societies might develop tighter norms for women compared with men.

## Implications of gender bias in cultural tightness

We suggest that gender bias in cultural tightness has important implications on gender inequality in leadership and innovation. Success in these two domains often requires challenging the status quo and revising extant rules and norms. Specifically, leadership is “the process (act) of influencing the activities of an organized group in its efforts toward goal setting and goal achievement” (44), and it invariably involves leading and managing changes (14, 45, 46). Similarly, innovators—those who successfully generate and implement novel and useful ideas (47)—are “rule breakers” who challenge accepted ways of doing things as they generate and implement creative ideas (13, 48–50). Thus, we suggest gender bias in cultural tightness is associated with gender inequality in business and political leadership and innovation, accounting for variations in key gender disparities across the 50 US states.

## Measuring gender bias in cultural tightness

We recruited participants across the 50 US states through Amazon's Mechanical Turk (MTurk). Although MTurk has been widely used in social science research (51–53), we acknowledge that their workers are not necessarily representative of the general populations (MTurk samples tend to be Internet users, who are younger and more educated). Additionally, MTurk sample composition varies with time, and there might be a concern of repeated participation (54, 55; see Discussion and Supplementary Text for more details on how we discussed and addressed these issues). This study was approved by the Institutional Review Board at Singapore Management University. We obtained informed consent from all participants.

A total of 15,425 individuals from a variety of occupations participated in the survey (57.31% women; 61.63% holding a bachelor's or higher degree; Mean_age_ = 35.94). Data were collected over two waves of survey 1 year and 2 months apart. We took two steps to ensure that each unique participant did not complete the questionnaire twice. First, we restricted the IP addresses for both waves of survey so that each participant can only participate once (i.e. their IP addresses need to be unique). Second, we checked participants’ IP addresses and MTurk IDs for both waves of survey and found no duplicate IPs and MTurk IDs. Because the measures of gender bias in cultural tightness were highly correlated (*r*_[48]_ = 0.86, *P* < 0.001, *n* = 50) and the results were highly consistent between the two waves of data collection (see Supplementary Text and Tables S12–S31), we combined the data in our analyses. On average, each state had 309 participants. This sample size is larger than or comparable to the sample size reported in previous similar research (2, 52, 56, 57). Detailed sample characteristics are reported in Supplementary Text.

To measure gender bias in cultural tightness, we used a six-item scale adapted from Gelfand et al.'s (2011) cultural tightness scale (1). For each item, we asked participants to rate, on a six-point scale, the extent to which women (compared with men) rather than himself/herself are constrained by a tighter culture in their state (e.g. “There are many more social norms that women [compared with men] are supposed to abide by in this state”; 1, “Strongly disagree,” and 6, “Strongly agree”). Results revealed that this measure had good reliability (Cronbach's *α* = 0.80). To test whether the items of gender bias in cultural tightness loaded on one factor, we conduced confirmatory factor analyses (CFAs) using both individual-level and multilevel analyses. Results showed that both individual-level CFA (*χ*^2^ = 124.52, *P* < 0.001, df = 9; CFI = 0.996, TLI = 0.99, RMSEA = 0.03, SRMR = 0.01) and multilevel CFA (*χ*^2^ = 118.58, *P* < 0.001, df = 18; CFI = 0.99, TLI = 0.99, RMSEA = 0.02, SRMR_[within]_ = 0.01) supported the fit of a single latent factor model with the data. Furthermore, results suggested significant state-level variations (Mean/Median *r*_wg[j]_ = 0.89/0.89; *F*_[49, 15375]_ = 17.45, *P* < 0.001; ICC[1] = 0.05; ICC[2] = 0.94); that is, people in some states perceive overall more constraints on women than men. In line with our theorizing, we aggregated participants’ responses to the state level and conducted subsequent analyses using the state-level values of gender bias in cultural tightness. *T*-test results further showed that the mean of the raw scores of state-level gender bias in cultural tightness across the 50 US states (Mean = 3.85, SD = 0.19) was larger than 3.50 (i.e. the mean of Likert 1 to 6 scale), *t*_[49]_ = 13.30, *P* < 0.001, indicating that women are generally more constrained than men at the state level.

In line with previous research (2), to avoid systematically extreme responses caused by potential regional factors and to facilitate the interpretation of our results, we first standardized the 50 states’ scores of gender bias in cultural tightness (i.e. *z*-scores). Next, we added three to each state's *z*-score to remove negative values and make the results more readable. With this transformation, gender bias in cultural tightness among the 50 states ranged from 1.27 to 5.36. This transformation process is commonly used in cross-cultural studies that develop cultural indexes (1, 2, 4). Higher score of gender bias in cultural tightness means that women are more constrained than men in a given state.

State-level gender bias in cultural tightness has a moderately positive correlation with general cultural tightness of the state (*r*_[48]_ = 0.60, *P* < 0.001, *n* = 50). This is likely because some religious (e.g. degree of religiosity) and political (e.g. conservatism) factors that are associated with cultural tightness are also related to gender bias in cultural tightness. However, this correlation is only moderate because other antecedents of cultural tightness may not have direct implications for gender bias in cultural tightness. In addition, gender bias in cultural tightness has a moderately negative correlation with the natural logarithm of gross domestic product (GDP) per capita (1977–2020) (*r*_[48]_ = −0.47, *P* < 0.001, *n* = 50), suggesting that states having lower economic development appear to place more constraints on women than men.

To check the divergent validity of gender bias in cultural tightness, we collected state-level data on the following: (i) gender equality, (ii) masculinity, and (iii) collectivism (see Supplementary Text on how each variable was measured and Table S1 for sources of each variable). Specifically, for state-level gender equality, we collected three gender equality scores^1^ from three sources: (i) state gender parity index from the RepresentWomen's Gender Parity Index 2019 Report, which measures women's recent electoral success at the local, state, and national levels; (ii) state gender equality score from the WalletHub 2020's Best and Worst States for Women's Equality Report, which measures the extent to which women receive equal treatments in three key domains (i.e. workplace environment, education and health, and political empowerment); and (iii) state municipal equality index from the Human Rights Campaign Foundation and the Equality Federation Institute in 2020, which measures the extent to which the state is embodying LGBTQ people inclusion in their laws, policies, and services. For state masculinity, we collected and computed gendered housework disparity ratio—the ratio of women's mean minutes to men's mean minutes in doing routine housework in each state from Ruppanner and Maume (2016) (58), which reflects the traditionalism in families. Highly masculine cultures tend to endorse traditional gender stereotypical views regarding gender role expectations in the family (e.g. women are expected to do more housework than men) (59, 60). As such, we used this ratio as a proxy of state masculinity. For state collectivism, we used the state collectivism index developed by Vandell and Cohen (1999) (61), which used eight items related to family structure and living arrangements, social, political, religious, and economic practices and then summed to create an overall collectivism score for each state. We found that gender bias in cultural tightness has moderately negative correlations with gender equality (for state gender parity index, *r*_[48]_ = −0.50, *P* < 0.001, *n* = 50; for state gender equality score, *r*_[48]_ = −0.62, *P* < 0.001, *n* = 50; for state municipal equality index, *r*_[48]_ = −0.47, *P* < 0.001, *n* = 50) but moderately positive correlations with state masculinity (*r*_[48]_ = 0.39, *P* = 0.005, *n* = 50). However, the relationship between gender bias in cultural tightness and state collectivism was not significant (*r*_[48]_ = 0.08, *P* = 0.593, *n* = 50). Overall, these results indicated that gender bias in cultural tightness appears different from these other gender-related constructs.

**Fig. 1.**
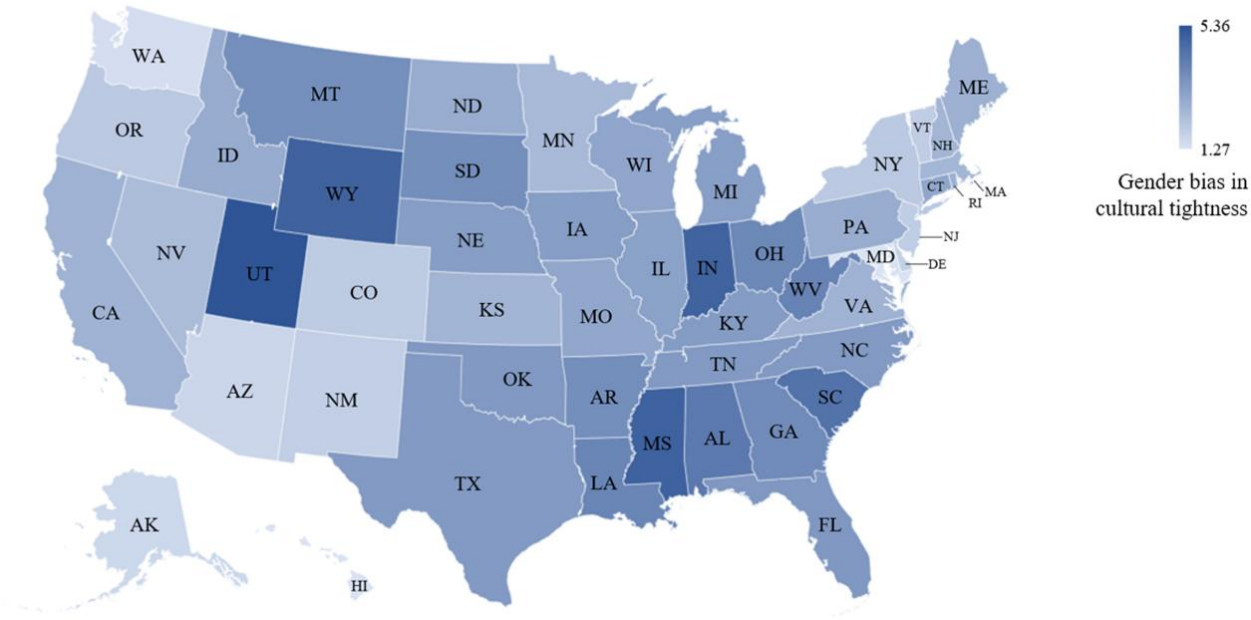
Gender bias in cultural tightness in the 50 US states.

Table 1 shows gender bias in cultural tightness across the 50 states, whereas Fig. 1 presents them on a map format. The 10 states with the largest gender bias in cultural tightness are Utah, Mississippi, Wyoming, Indiana, South Carolina, Alabama, West Virginia, Louisiana, Ohio, and Georgia. The 10 states with the smallest gender bias in cultural tightness are Maryland, Hawaii, Washington, Alaska, Arizona, Delaware, New Mexico, Vermont, New Jersey, and Colorado.

**Table 1.** Gender bias in cultural tightness in the 50 US states.

State	Number of participants	Mean age	Percentage of men	Gender bias in cultural tightness
Alabama	304	36.87	0.41	4.29
Alaska	302	33.30	0.52	1.60
Arizona	309	36.40	0.46	1.63
Arkansas	305	36.44	0.37	3.69
California	332	35.77	0.51	2.65
Colorado	305	35.84	0.40	1.96
Connecticut	301	33.41	0.36	2.99
Delaware	303	36.32	0.45	1.79
Florida	319	38.95	0.45	3.43
Georgia	308	38.02	0.37	3.78
Hawaii	303	34.38	0.44	1.28
Idaho	300	33.71	0.44	2.86
Illinois	307	37.61	0.49	3.15
Indiana	308	35.30	0.42	4.93
Iowa	306	36.90	0.36	3.26
Kansas	308	36.71	0.38	2.58
Kentucky	314	38.18	0.43	3.29
Louisiana	304	36.00	0.40	3.95
Maine	304	34.44	0.38	2.70
Maryland	307	36.26	0.46	1.27
Massachusetts	310	35.37	0.42	2.53
Michigan	312	36.96	0.37	3.22
Minnesota	309	35.25	0.43	2.46
Mississippi	309	35.25	0.44	4.97
Missouri	306	37.44	0.37	3.00
Montana	307	35.29	0.46	3.66
Nebraska	304	35.14	0.44	3.25
Nevada	312	35.70	0.43	2.36
New Hampshire	308	34.04	0.43	2.56
New Jersey	309	36.00	0.48	1.91
New Mexico	306	35.11	0.47	1.83
New York	323	36.14	0.46	2.00
North Carolina	313	38.49	0.37	3.36
North Dakota	306	34.04	0.46	2.83
Ohio	315	36.58	0.42	3.87
Oklahoma	309	35.77	0.39	3.39
Oregon	309	36.43	0.40	2.01
Pennsylvania	315	39.02	0.37	2.83
Rhode Island	303	33.95	0.45	2.71
South Carolina	305	36.27	0.41	4.49
South Dakota	309	35.05	0.46	3.68
Tennessee	305	36.92	0.38	3.34
Texas	336	36.40	0.48	3.36
Utah	309	33.07	0.50	5.36
Vermont	302	36.90	0.43	1.90
Virginia	307	36.14	0.43	2.66
Washington	305	36.35	0.42	1.40
West Virginia	311	34.91	0.42	4.00
Wisconsin	307	37.57	0.39	3.01
Wyoming	295	34.14	0.48	4.95
Mean	309	35.93	0.43	3.00

*Note.* Higher score indicates greater gender bias in cultural tightness (i.e. women are more constrained than men in the given state).

We next tested whether there were differences in gender bias in cultural tightness at the regional (i.e. Northeast, Midwest, South, and West; see Fig. S1) and divisional levels (e.g. New England and South Atlantic; see Fig. S2), given that previous research has found that parts of the regions in the United States are associated with specific cultures (4). An ANOVA test indicated significant differences in gender bias in cultural tightness among the four primary regions—Northeast, Midwest, South, and West—recognized by the U.S. Census Bureau, *F*_(3, 46)_ = 3.41, *P* = 0.025, *η*^2^ = 0.18. Gender bias in cultural tightness for the four primary regions was as follows (from the largest bias to the smallest bias): South region (*n* = 16, Mean = 3.44, SD = 0.93, 95% confidence interval [CI] [2.95, 3.94]), Midwest region (*n* = 12, Mean = 3.27, SD = 0.66, 95% CI [2.85, 3.69]), West region (*n* = 13, Mean = 2.58, SD = 1.32, 95% CI [1.78, 3.38]), and Northeast region (*n* = 9, Mean = 2.46, SD = 0.42, 95% CI [2.14, 2.78]). Results of Tukey's honestly significant difference (HSD) post hoc tests further demonstrated that while the South region score had marginally significant differences with the Northwest region score (*Δ*_mean_ = 0.98, SE = 0.39, *P* = 0.069) and the West region score (*Δ*_mean_ = 0.86, SE = 0.35, *P* = 0.078), there was no significant difference between any two of these four regions (see Table S2 for all descriptive statistics).

However, when investigating the differences of gender bias in cultural tightness at a more specific regional division level, a Welch ANOVA (Levene's test, *F*_[8, 41]_ = 3.84, *P* = 0.002) using the U.S. Census's nine regional divisions (i.e. New England, Middle Atlantic, East North Central, West North Central, South Atlantic, East South Central, West South Central, Mountain, and Pacific) indicated significant differences in gender bias in cultural tightness, (*F*_(8, 14.38)_ = 6.26, *P* = 0.001, *η*^2^ = 0.36). Games–Howell post hoc tests demonstrated that the Pacific (*n* = 5, Mean = 1.79, SD = 0.56, 95% CI [1.10, 2.48]) had the smallest gender bias in cultural tightness and was significantly different compared with the East North Central (*n* = 5, Mean = 3.64, SD = 0.80, 95% CI [2.65, 4.63], *Δ*_mean_ = −1.85, SE = 0.43, *P* = 0.048) and the West South Central (*n* = 4, Mean = 3.60, SD = 0.28, 95% CI [3.16, 4.04], *Δ*_mean_ = −1.81, SE = 0.29, *P* = 0.009). Furthermore, the New England score (*n* = 6, Mean = 2.57, SD = 0.37, 95% CI [2.18, 2.95]) was significantly lower compared with the West South Central score (*Δ*_mean_ = −1.03, SE = 0.20, *P* = 0.016) (see Table S3 for all descriptive statistics).

## Correlates of gender bias in cultural tightness

Gender bias in cultural tightness is likely associated with a variety of sociopolitical factors (religion and political ideology) and gender-related threats at the state level. We collected state-level variables pertaining to these factors (their corresponding data sources are shown in Table S1) and conducted two sets of ordinary least squares (OLS) regression to test the effects of these variables on gender bias in cultural tightness. For each analysis, we first conducted regressions without any control variables, before controlling for GDP per capita and gender imbalance in population in the states (i.e. 1—the number of women in population/the number of men in population). We controlled for GDP per capita because low levels of economic development are often associated with higher levels of gender inequality (62). Furthermore, we controlled for gender imbalance in population (i.e. more men than women) because it may potentially be associated with gender bias in cultural tightness in either direction. For example, a state with more men may mean that potentially more people endorse patriarchal values (34, 36), thereby producing more constraints on women, or, conversely, a state with more men may decrease gender bias in cultural tightness, as men would have to cater to women who are the minority.

### Sociopolitical factors: religion

Conservative religions tend to endorse traditional gender roles for women (32, 33), which is in turn positively associated with stronger constraints on women. Amid this backdrop, we suggest that state religiosity is positively linked to gender bias in cultural tightness. In line with Harrington and Gelfand (2014), we adopted two measures for state religiosity (4). Specifically, we collected data from the Pew Research Center (2014) that show the percentage of adults who are highly religious, importance of religion, frequency of prayer, worship attendance, belief in God, religious belief, and a breakdown religious data of key religious affiliation. In addition, from the Gallup (2016), we collected the percentage of adults who are very religious, moderately religious, and nonreligious. As shown in Table 2, the percentage of adults who are highly religious (*b* = 4.46, SE = 1.22, *P* < 0.001), importance of religion (*b* = 4.41, SE = 1.27, *P* = 0.001), frequency of prayer (*b* = 4.82, SE = 1.37, *P* < 0.001), worship attendance (*b* = 7.58, SE = 1.53, *P* < 0.001), belief in God (*b* = 4.50, SE = 1.42, *P* = 0.003), and religious belief (*b* = 7.10, SE = 2.27, *P* = 0.003) were all positively related to gender bias in cultural tightness. Also, as shown in Table 3, the percentage of adults who are nonreligious (*b* = −6.97, SE = 2.32, *P* = 0.004) was negatively related to gender bias in cultural tightness. As shown in Table 4 (Gallup data), the percentage of adults who are very religious (*b* = 6.48, SE = 1.33, *P* < 0.001) was positively related to gender bias in cultural tightness, whereas the percentage of adults who are nonreligious (*b* = −5.47, SE = 1.24, *P* < 0.001) was negatively related to gender bias in cultural tightness. The percentage of adults who are moderately religious (*b* = 0.60, SE = 4.56, *P* = 0.895) was not significantly related to gender bias in cultural tightness.

**Table 2.** Links between religion (Pew) and gender bias in cultural tightness.

Variables	Model 1	Model 2	Model 3	Model 4	Model 5	Model 6	Model 7	Model 8	Model 9	Model 10	Model 11	Model 12
Percentage of adults who are highly religious^a^	**5**.51*** (1.08)	**4**.46*** (1.22)										
Importance of religion			**5**.45*** (1.09)	**4**.41** (1.27)								
Frequency of prayer					**6**.07*** (1.25)	**4**.82*** (1.37)						
Worship attendance							**8**.83*** (1.41)	**7**.58*** (1.53)				
Belief in God									**5**.83*** (1.26)	**4**.50** (1.42)		
Religious belief											**8**.49*** (2.17)	**7**.10** (2.27)
GDP per capita (log): 1977–2020		−1.34^+^ (0.74)		−1.29^+^ (0.76)		−1.47^+^ (0.73)		−1.26^+^ (0.66)		−1.40^+^ (0.77)		−2.03** (0.71)
Gender imbalance in population: 1970–2020 (more men than women)		0.12 (3.61)		0.51 (3.67)		−0.35 (3.62)		−0.18 (3.30)		−0.71 (3.69)		2.34 (3.90)
Constant	−0.02 (0.60)	14.66^+^ (8.13)	0.13 (0.58)	14.21^+^ (8.37)	−0.29 (0.69)	15.88^+^ (8.08)	−0.16 (0.52)	13.53^+^ (7.23)	−0.69 (0.81)	14.82^+^ (8.60)	−3.47* (1.66)	18.97* (8.01)
*N*	50	50	50	50	50	50	50	50	50	50	50	50
*R* ^2^	0.35	0.40	0.34	0.38	0.33	0.39	0.45	0.49	0.31	0.36	0.24	0.36
*F*	25.92	10.02	25.10	9.48	23.41	9.63	38.94	14.78	21.42	8.60	15.30	8.50

^+^ *P* < 0.1; ^*^P < 0.05; ^**^P < 0.01; ***P < 0.001. Results from OLS regressions. Unstandardized regression coefficients are reported. Standard errors in parentheses. The bold values are coefficients of the relationships between the focal variables and gender bias in cultural tightness. ^a^Religious data in this table were obtained and computed from the Pew Research Center (2014).

**Table 3.** Links between religion (Pew, breakdown of key religious affiliation) and gender bias in cultural tightness.

Variables	Model 1	Model 2	Model 3	Model 4	Model 5	Model 6	Model 7	Model 8	Model 9	Model 10	Model 11	Model 12	Model 13	Model 14	Model 15	Model 16	Model 17	Model 18
Nonreligious	**−8**.38*** (2.23)	**−6**.97** (2.32)																
Buddhists^a,b,c^			**−25**.68* (11.63)	**−16**.**83** **(11.54)**														
Catholics					**−5**.08** (1.47)	**−3**.76* (1.69)												
Evangelical Protestants							**3**.73** (1.20)	**2**.**10** **(1.36)**										
Hindus									**−63**.83** (19.74)	**−56**.86* (23.54)								
Black Protestants											**5**.27* (2.38)	**4**.**67**^+^ **(2.49)**						
Jews													**−27**.54** (8.97)	**−29**.57* (11.02)				
Mainline Protestants															**0**.**13** **(2.47)**	**−0**.**73** **(2.24)**		
Mormons																	**3**.71* (1.70)	**3**.99* (1.63)
GDP per capita (log): 1977–2020		−2.07** (0.71)		−2.32** (0.75)		−1.45 (0.87)		−1.82* (0.86)		−1.28 (0.88)		−2.48** (0.73)		−1.13 (0.87)		−2.53** (0.76)		−2.15** (0.73)
Gender imbalance in population: 1970–2020 (more men than women)		2.27 (3.93)		−0.03 (4.12)		−4.23 (4.03)		−1.91 (3.97)		−6.54 (4.33)		2.23 (4.42)		−8.76^+^ (4.62)		−1.64 (4.06)		−5.24 (4.10)
Constant	4.93*** (0.53)	26.44*** (7.40)	3.18*** (0.16)	27.49** (7.95)	3.96*** (0.31)	18.79* (8.98)	2.03*** (0.34)	21.52* (9.32)	3.23*** (0.15)	16.48^+^ (9.25)	2.71*** (0.19)	28.87*** (7.74)	3.33*** (0.17)	14.95 (9.19)	2.98*** (0.43)	29.70*** (8.11)	2.89*** (0.14)	25.36** (7.72)
*N*	50	50	50	50	50	50	50	50	50	50	50	50	50	50	50	50	50	50
*R* ^2^	0.23	0.35	0.09	0.25	0.20	0.30	0.17	0.26	0.18	0.31	0.09	0.28	0.16	0.33	0.00	0.22	0.09	0.31
*F*	14.13	8.18	4.87	5.23	11.98	6.43	9.65	5.34	10.46	6.81	4.91	5.82	9.42	7.40	0.00	4.36	4.77	6.88

^+^ *P* < 0.1; ^*^P < 0.05; ^**^P < 0.01; ^***^P < 0.001. Results from OLS regressions. Unstandardized regression coefficients are reported. Standard errors in parentheses. The bold values are coefficients of the relationships between the focal variables and gender bias in cultural tightness. ^a^Religious data in this table were obtained and computed from the Pew Research Center (2014). ^b^Some other minor religions (e.g., Jehovah's Witness) were considered but not significant. ^c^To make the variables continuous, the values that were <1% were replaced by zero.

**Table 4.** Links between religion (Gallup) and gender bias in cultural tightness.

Variables	Model 1	Model 2	Model 3	Model 4	Model 5	Model 6
Percentage of adults who are very religious^a^	7.36*** (1.**14)**	6.48*** (1.**33)**				
Percentage of adults who are moderately religious			**0.04** **(4**.**71)**	**0.60** **(4**.**56)**		
Percentage of adults who are nonreligious					−6.39*** (1.**10)**	−5.47*** (1.**24)**
GDP per capita (log): 1977–2020		−0.85 (0.70)		−2.53** (0.77)		−1.26^+^ (0.70)
Gender imbalance in population: 1970–2020 (more men than women)		−0.53 (3.31)		−1.43 (4.35)		1.02 (3.46)
Constant	0.13 (0.45)	9.43 (7.69)	2.99* (1.35)	29.35*** (8.03)	5.09*** (0.38)	18.07* (7.19)
*N*	50	50	50	50	50	50
*R* ^2^	0.47	0.49	0.00	0.22	0.41	0.45
*F*	42.03	14.47	0.00	4.33	33.79	12.66

^+^ *P* < 0.1; ^*^P < 0.05; ^**^P < 0.01; ^***^P < 0.001. Results from OLS regressions. Unstandardized regression coefficients are reported. Standard errors in parentheses. The bold values are coefficients of the relationships between the focal variables and gender bias in cultural tightness. ^a^Religious data in this table were obtained and computed from the Gallup Daily Tracking (2016).

### Sociopolitical factors: political ideology

Gender bias in cultural tightness is also reflected in political institutions, ideologies, and practices. Specifically, states with political conservatives tend to endorse more patriarchal values, which are related to gender bias in cultural tightness (34). Thus, we suggest that states where conservatives make up a larger share of the population are more likely to have larger gender bias in cultural tightness. To analyze this, we collected data from the Pew Research Center (2014) showing state-level data on the percentage of people who hold conservative beliefs. Results in Table 5 indicated that the percentage of conservatives was positively associated with gender bias in cultural tightness (*b* = 11.65, SE = 1.81, *P <* 0.001).

**Table 5.** Links between political ideology and gender bias in cultural tightness.

Variables	Model 1	Model 2	Model 3	Model 4	Model 5	Model 6
Percentage of people having conservative political ideology^a^	11.08*** (1.**48)**	11.65*** (1.**81)**				
Percentage of Republicans in the U.S. Senate			1.37*** (0.**25)**	1.28*** (0.**26)**		
Percentage of Republicans in the House of Representatives					1.70*** (0.**32)**	1.74*** (0.**33)**
GDP per capita (log): 1977–2020		0.24 (0.70)		−1.08 (0.67)		−0.89 (0.67)
Gender imbalance in population: 1970–2020 (more men than women)		−7.18* (3.07)		−6.12^+^ (3.39)		−8.63* (3.48)
Constant	−1.14* (0.56)	−4.14 (7.81)	2.28*** (0.17)	13.43^+^ (7.20)	2.11*** (0.20)	11.13 (7.23)
*N*	50	50	50	50	50	50
*R* ^2^	0.54	0.59	0.39	0.50	0.38	0.51
*F*	55.72	22.03	31.27	15.03	29.04	16.14

^+^ *P* < 0.1; ^*^P < 0.05; ^**^P < 0.01; ^***^P < 0.001. Results from OLS regressions. Unstandardized regression coefficients are reported. Standard errors in parentheses. The bold values are coefficients of the relationships between the focal variables and gender bias in cultural tightness. ^a^Percentage of people having conservative political ideology was obtained and computed from the Pew Research Center (2014), while percentages of republicans in the U.S. Senate and in the House of Representatives were obtained and computed from the Biographical Directory of the United States Congress (2019–2021).

In addition, we collected and computed the proportion of Republicans in the U.S. Senate and House of Representatives from the Biographical Directory of the United States Congress (2019–2021, i.e. the 116th Congress). Results in Table 5 indicated that the percentage of Republicans in the U.S. Senate (*b* = 1.28, SE = 0.26, *P <* 0.001) and in the House of Representatives (*b* = 1.74, SE = 0.33, *P <* 0.001) was all positively related to gender bias in cultural tightness. In sum, these results suggested that states with more people embracing conservative political ideology appear to have larger gender bias in cultural tightness.

### Gender-related threats

Gender-related social threats including both benevolent sexism and hostile sexism are rooted in a belief that “women inhabit restricted domestic roles and are the ‘weak’ sex” (36). They serve to justify men's power, control, and dominance. Thus, women in states where either form of sexism is commonplace are likely to experience greater emphasis on traditional gender roles and hence greater constraints on them. As such, we suggest that sexism is positively related to gender bias in cultural tightness.

A variable related to sexism is societies’ tolerance toward sexual diversity. Societies that have more open attitudes toward lesbians, gays, bisexuals, and transgender (LGBT) individuals are also likely to have more liberal attitudes toward women (63, 64). In addition, societies where people have fewer negative views about those who do not assume traditional gender roles tend to have a less patriarchal culture (63) and thus fewer constraints on women. Accordingly, women in such societies may experience fewer social threats. Thus, we suggest that states that are in favor of protecting LGBT individuals from discrimination would likely have smaller gender bias in cultural tightness.

To test these propositions, we collected data on state-level sexism from (i) the World Value Survey (2017) (i.e. state sexism belief i) and (ii) the DDB Needham Life Style Survey (1975–1998) (i.e. state sexism belief ii). Specifically, state sexism belief i comprised five items that reflected patriarchal gender roles and gender stereotype from the World Value Survey (e.g. “On the whole, men make better political leaders than women do”), whereas state sexism belief ii comprised other five items that reflected patriarchal gender roles and gender stereotype from the DDB Needham Life Style Survey (e.g. “Women's place is in the home”). We also examined statistics from the American Values Atlas (2019) regarding the percentage of people who favor laws protecting the LGBT community from discrimination, as well as data from the Pew Research Center (2014) on the percentage of people viewing homosexuality as acceptable. As shown in Table 6, the scores of sexism belief were all positively related to gender bias in cultural tightness (for state sexism belief i, *b* = 3.27, SE = 0.93, *P* = 0.001; for state sexism belief ii, *b* = 4.74, SE = 1.00, *P <* 0.001); positive attitudes toward LGBT individuals were all negatively related to gender bias in cultural tightness (for percentage of people favoring nondiscrimination LGBT protection, *b* = −6.67, SE = 3.16, *P* = 0.040; for percentage of people viewing homosexuality as acceptable, *b* = −5.82, SE = 1.43, *P <* 0.001).

**Table 6.** Links between gender-related threats and gender bias in cultural tightness.

Variables	Model 1	Model 2	Model 3	Model 4	Model 5	Model 6	Model 7	Model 8	Model 9	Model 10	Model 11	Model 12	Model 13	Model 14	Model 15	Model 16
State sexism belief i^a^	3.84*** (0.**96)**	3.27** (0.**93)**														
State sexism belief ii			4.55*** (0.**76)**	4.74*** (1.**00)**												
Percentage of people favoring nondiscrimination LGBT protection					−7.89* (3.**04)**	−6.67* (3.**16)**										
Percentage of people viewing homosexuality as acceptable							−6.62*** (1.**18)**	−5.82*** (1.**43)**								
Percentage of male-dominated industries: 2001–2018									13.07*** (3.**26)**	13.03*** (3.**64)**						
Sexual violence against women											−**0.07**^+^ **(0**.**04)**	−**0.06** **(0**.**05)**				
Relative domestic violence													**0.36** **(0**.**40)**	**0.23** **(0**.**36)**		
Relative human trafficking															−**0.32**^+^ **(0**.**19)**	−**0.23** **(0**.**18)**
GDP per capita (log): 1977–2020		−1.93* (0.74)		0.09 (0.84)		−1.84* (0.79)		−0.69 (0.79)		−1.07 (0.78)		−2.02^+^ (1.02)		−2.59** (0.75)		−2.30** (0.76)
Gender imbalance in population: 1970–2020 (more men than women)		−2.45 (4.15)		−4.20 (4.06)		−4.94 (4.18)		−3.43 (3.51)		−7.99^+^ (4.01)		2.64 (5.67)		−1.39 (3.99)		−2.31 (4.02)
Constant	−4.82* (1.96)	16.47^+^ (8.42)	−10.04*** (2.19)	−11.71 (10.88)	8.56*** (2.15)	26.90** (7.75)	7.01*** (0.73)	13.62^+^ (7.90)	0.14 (0.72)	11.16 (8.72)	5.08*** (1.17)	25.96* (10.81)	2.53*** (0.56)	29.90** (7.97)	3.63*** (0.40)	27.61** (7.99)
*N*	49	49	48	48	50	50	50	50	50	50	41	41	49	49	50	50
*R* ^2^	0.25	0.36	0.44	0.45	0.12	0.29	0.39	0.43	0.25	0.39	0.07	0.16	0.02	0.25	0.06	0.25
*F*	16.04	8.50	36.01	12.12	6.72	6.22	31.28	11.35	16.10	9.81	3.00	2.39	0.82	4.98	2.88	5.05

^+^ *P* < 0.1; ^*^P < 0.05; ^**^P < 0.01; ^***^P < 0.001. Results from OLS regressions. Unstandardized regression coefficients are reported. Standard errors in parentheses. The bold values are coefficients of the relationships between the focal variables and gender bias in cultural tightness. ^a^State sexism belief i was obtained and computed from the World Value Survey (2017), whereas state sexism belief ii was obtained and computed from the DDB Needham Life Style Survey (1975–1998). Percentage of people favoring nondiscrimination LGBT protection was obtained from Research on LGBT in the PRRI American Values Atlas (2019). Percentage of people viewing homosexuality as acceptable was obtained and computed from the Pew Research Center (2014). Percentage of male-dominated industries was obtained and computed from the NAICS Industry data from the Bureau of Economic Analysis (2001–2018). Sexual violence against women and relative domestic violence were obtained and computed from the National Intimate Partner and Sexual Violence Survey (2010). Relative human trafficking was obtained and computed from the National Human Trafficking Hotline (2018).

Male overrepresentation in a given domain may also present a form of social threats for women who are in the minority. We thus suggest that states with a higher percentage of male-dominated industries are more likely to have larger gender bias in cultural tightness. Male-dominated industries refer to the workforce domains where women constitute less than one-fourth of the total workforce (65). Examples include the mining, quarrying, and oil/gas extraction industries as well as forestry, fishing, and related industries (66). Societies that have more male-dominated industries also tend to have more ingrained masculine gender role identities and patriarchal values (67). We collected the percentage of male-dominated industries in each state from total full-time and part-time employment data published by the U.S. Bureau of Economic Analysis (2001–2018) using the North American Industry Classification System (NAICS). Results in Table 6 indicated that the percentage of male-dominated industries was indeed positively related to gender bias in cultural tightness (*b* = 13.03, SE = 3.64, *P <* 0.001).

To examine the relationship between gender-related physical threats and gender bias in cultural tightness, we collected and computed data on state sexual violence against women (i.e. the weighted percentage of women victimization on sexual violence), relative domestic violence (i.e. the ratio of women victimization to men victimization) from the National Intimate Partner and Sexual Violence Survey (2010), and relative human trafficking (i.e. the ratio of women victimization to men victimization) from the National Human Trafficking Hotline (2018). Results in Table 6 showed that the relationship between physical threats and gender bias in cultural tightness was not significant (for sexual violence against women, *b* = −0.06, SE = 0.05, *P* = 0.223; for relative domestic violence, *b* = 0.23, SE = 0.36, *P* = 0.530; and for relative human trafficking, *b* = −0.23, SE = 0.18, *P* = 0.198). Our interpretation of this finding is that unlike gender-related social threats such as sexism which are pervasive in societies, gender-related physical threats are comparatively less common. As such, the relationship between physical threats and gender bias in cultural tightness is not particularly strong. Gender bias in cultural tightness in American societies appears more associated with gender-related social threats than physical threats.

## Links to gender equality in leadership and innovation

We next explored the implications of gender bias in cultural tightness among the 50 US states. Specifically, we investigated the relationships between gender bias in cultural tightness and gender inequality regarding leadership and innovation at the state level. Given that a larger gender bias in cultural tightness implies stronger norms and less tolerance of aberrant behaviors for women than men, we suggest that gender bias in cultural tightness will be positively associated with gender inequality in areas wherein the status quo needs to be challenged and rules and norms need to be revised, such as in leadership and innovation. All variables and their corresponding data sources were shown in Table S1. We used the following equation to calculate gender inequality in all our state-level dependent variables, with higher scores indicating that fewer women are represented in a particular area, *Y* (i.e. leadership or innovation):


$$\mathrm{The}\;\mathrm{gender}\;\mathrm{inequality}\;\mathrm{in}\;Y=1\;-\;\frac{\mathrm{The}\;\mathrm{number}\;\mathrm{of}\;\mathrm{women}\;\mathrm{in}\;Y}{\mathrm{The}\;\mathrm{number}\;\mathrm{of}\;\mathrm{men}\;\mathrm{in}\;Y}$$

To investigate the relationships between gender bias in cultural tightness and gender inequality in leadership and innovation, we conducted two sets of analyses using hierarchical linear modeling (HLM). For each dependent variable, we first conducted regression without any control variables; then, we controlled for GDP per capita, gender imbalance in population, and the general state-level cultural tightness scores from Harrington and Gelfand (2014) (4). We controlled for GDP per capita given the evidence that low levels of economic development increase gender inequality (62). We also controlled for gender imbalance in population because unequal gender distribution (i.e. more men than women) means men have more advantages and dominance over women, leading to greater gender inequality in leadership and innovation (8, 12). We sought to isolate the unique effect of gender bias in cultural tightness by controlling for other aspects of state cultural tightness. In doing so, we aim to demonstrate that gender bias in cultural tightness has explanatory power over and beyond a general measure of state cultural tightness. In all models, to exclude the influence of aggregate (i.e. time-series) trends, we controlled for the year fixed effects by coding each of the years as dummy variables.

### Gender inequality in business and political leadership

To investigate the relationships between gender bias in cultural tightness and gender inequality in leadership at the state level, we focus on two distinct leadership domains: business leadership and political leadership (68, 69). For business leadership, we collected data from the Institutional Shareholder Services (ISS) and Compustat (2007–2020) to compute the gender inequality in corporate boards and chief executive officers (CEOs) of publicly traded companies. Furthermore, we used data from the U.S. Census Bureau's American Community Survey (ACS; 2005–2019) to compute gender inequality in different management-level occupations, including top executives.

As shown in Table 7, our results indicated that gender bias in cultural tightness was positively associated with gender inequality in boards of publicly traded companies (*b* = 0.03, SE = 0.01, *P* = 0.010), as well as CEOs of such companies (*b* = 0.04, SE = 0.02, *P* = 0.046). Furthermore, the relationship between gender bias in cultural tightness and gender inequality in management occupations was significantly positive (*b* = 0.03, SE = 0.01, *P* = 0.002). In addition, states with larger gender bias in cultural tightness also had greater gender inequality in executives in top management teams (*b* = 0.03, SE = 0.01, *P <* 0.001).

**Table 7.** Links between gender bias in cultural tightness and gender inequality in business leadership.

Variables	Gender inequality in boards of publicly traded companies^a^		Gender inequality in CEOs of publicly traded companies		Gender inequality in management occupations: total		Gender inequality in management occupations: top executives		Gender inequality in business leadership (aggregated)^b^	
	Model 1	Model 2	Model 3	Model 4	Model 5	Model 6	Model 7	Model 8	Model 9	Model 10
Gender bias in cultural tightness	0.03** (0.**01)**	0.03* (0.**01)**	0.03* (0.**01)**	0.04* (0.**02)**	0.04*** (0.**01)**	0.03** (0.**01)**	0.03*** (0.**00)**	0.03*** (0.**01)**	0.30*** (0.**07)**	0.31*** (0.**07)**
GDP per capita (log)		0.18*** (0.04)		0.45*** (0.08)		−0.05 (0.03)		0.04 (0.03)		0.63** (0.23)
Gender imbalance in population (more men than women)		0.52^+^ (0.28)		0.85^+^ (0.48)		1.16*** (0.25)		0.09 (0.16)		7.32*** (1.59)
State cultural tightness		0.00 (0.00)		0.00* (0.00)		0.00 (0.00)		0.00 (0.00)		0.01 (0.01)
Constant	0.55*** (0.03)	−1.47*** (0.44)	0.82*** (0.04)	−4.29*** (0.87)	0.23*** (0.03)	0.75* (0.36)	0.50*** (0.02)	0.08 (0.31)	−1.57*** (0.21)	−8.70*** (2.62)
Year	2007–2020	2007–2020	2007–2020	2007–2020	2005–2019	2005–2019	2005–2019	2005–2019	2007–2019	2007–2019
*N*	676	676	664	664	750	750	750	750	629	629

^+^ *P* < 0.1; ^*^P < 0.05; ^**^P < 0.01; ^***^P < 0.001. Results from hierarchical linear modeling. Unstandardized regression coefficients are reported. Standard errors in parentheses. The bold values are coefficients of the relationships between the focal variables and gender bias in cultural tightness. ^a^Gender inequality in boards and CEOs of publicly traded companies was obtained and computed from the Institutional Shareholder Services (ISS) and Compustat (2007–2020), while gender inequality in management occupations, including top executives, was obtained and computed from the U.S. Census Bureau's American Community Survey (2005–2019). ^b^Gender inequality in business leadership was calculated by taking an average of the standardized gender inequality in boards of publicly traded companies and total management occupations.

We also calculated gender inequality in business leadership by taking an average of the standardized gender inequality in boards of publicly traded companies and total management occupations. Results indicated that gender bias in cultural tightness was positively related to gender inequality in overall business leadership (*b* = 0.31, SE = 0.07, *P <* 0.001).

For political leadership, we used data from the Center for American Women and Politics (CAWP) to compute gender inequality in US Senators and Representatives (1901–2020), as well as in State Senators, Representatives (1975–2020), and Governors (1901–2020). As shown in Table 8, gender bias in cultural tightness was positively related to gender inequality in US Senators (*b* = 0.07, SE = 0.03, *P* = 0.017), State Senators (*b* = 0.05, SE = 0.01, *P* = 0.001), State Representatives (*b* = 0.04, SE = 0.01, *P* = 0.001), and State Governors (*b* = 0.75, SE = 0.37, *P* = 0.043), but not related to gender inequality in US Representatives (*b* = 0.02, SE = 0.02, *P* = 0.503). Similar as above, we calculated gender inequality in political leadership by taking an average of the standardized gender inequality in US Senators and Representatives as well as State Senators, Representatives, and Governors. Results indicated that gender bias in cultural tightness was positively related to gender inequality in overall political leadership (*b* = 0.18, SE = 0.05, *P <* 0.001). Taken together, the above results are consistent with our propositions that states with tighter constraints on women than men have fewer women leaders in business and politics.

**Table 8.** Links between gender bias in cultural tightness and gender inequality in political leadership.

Variables	Gender inequality in US Senators^a^		Gender inequality in US Representatives		Gender inequality in State Senators		Gender inequality in State Representatives		Gender inequality in State Governors		Gender inequality in political leadership (aggregated)^b^	
	Model 1	Model 2	Model 3	Model 4	Model 5	Model 6	Model 7	Model 8	Model 9	Model 10	Model 11	Model 12
Gender bias in cultural tightness	0.05* (0.**02)**	0.07* (0.**03)**	0.06** (0.**02)**	**0.02** **(0**.**02)**	0.07*** (0.**01)**	0.05** (0.**01)**	0.07*** (0.**01)**	0.04** (0.**01)**	0.61* (0.**29)**	0.75* (0.**37)**	0.30*** (0.**05)**	0.18*** (0.**05)**
GDP per capita (log)		0.05 (0.08)		0.03 (0.06)		0.02 (0.03)		0.11*** (0.03)		1.56 (1.21)		0.24* (0.12)
Gender imbalance in population (more men than women)		0.11 (0.56)		0.69 (0.43)		0.33 (0.22)		0.01 (0.19)		5.13 (7.75)		0.96 (0.83)
State cultural tightness		−0.00 (0.00)		0.01** (0.00)		0.00*** (0.00)		0.01*** (0.00)		0.00 (0.03)		0.02*** (0.00)
Constant	0.85*** (0.08)	0.37 (0.88)	0.78*** (0.07)	0.33 (0.66)	0.72*** (0.04)	0.41 (0.32)	0.67*** (0.04)	−0.62* (0.27)	2.48* (1.17)	−13.30 (12.45)	−0.16 (0.16)	−3.06* (1.21)
Year	1977–2020	1977–2020	1977–2020	1977–2020	1977–2020	1977–2020	1977–2020	1977–2020	1977–2020	1977–2020	1977–2020	1977–2020
*N*	2117	2117	2143	2143	2049	2049	2008	2008	2200	2200	1915	1915

^+^ *P* < 0.1; ^*^P < 0.05; ^**^P < 0.01; ^***^P < 0.001. Results from hierarchical linear modeling and hierarchical logistic modeling (Models 11 and 12). Unstandardized regression coefficients are reported. Standard errors in parentheses. The bold values are coefficients of the relationships between the focal variables and gender bias in cultural tightness. ^a^Gender inequality in US Senators and Representatives (1901–2020) and state Senators, Representatives (1975–2020), and state Governors (1901–2020) was obtained and computed from the Center for American Women and Politics (CAWP). ^b^Gender inequality in political leadership was calculated by taking an average of the standardized gender inequality in US Senators and Representatives and state Senators, Representatives, and Governors.

### Gender inequality in innovation

To examine the relationships between gender bias in cultural tightness and gender inequality in innovation, we began by collecting three sets of data: patent success reported by the U.S. Patent and Trademark Office (USPTO; 2008–2020), STEM (science, technology, engineering, and mathematics) occupations from the American Community Survey (ACS; 2005–2019), and higher education attainment from the Current Population Survey (CPS; 2003–2020) reported by the U.S. Census Bureau. We are particularly interested in the attainment of doctoral degrees as these degrees typically involve research and creation of new knowledge. We then computed gender inequality in these three sets of data.

For patent success, we considered three types of patents tracked by the USPTO: (i) the utility patent, granted for invention or discovery of any new and useful process, machine, article of manufacture, or composition of matter, (ii) the design patent, granted for invention of a new, original, and ornamental design for an article of manufacture, and (iii) the plant patent, granted for invention, discovery, or asexual reproduction of any distinct and new variety of plant. To assess the gender of inventors, the USPTO first disambiguated inventors’ names by identifying unique inventors through a series of discriminative algorithms and methods. Subsequently, using disambiguated patent inventor names and name-gender linked data from the Global Name Recognition system, a name-search technology produced by IBM (IBM-GNR), and the WIPO worldwide gender-name dictionary (WGND), the patent office was able to identify the gender of roughly 93% of inventors (70). Our analyses were based on patents wherein the gender of the inventors was previously identified using the above method.

Table 9 showed that gender bias in cultural tightness was positively related to gender inequality in success for patents (*b* = 0.01, SE = 0.004, *P* = 0.004). Specifically, gender bias in cultural tightness was positively related to gender inequality in utility patent success (*b* = 0.01, SE = 0.003, *P <* 0.001) but not significantly related to gender inequality in design patent success (*b* = −0.01, SE = 0.01, *P* = 0.714) or plant patent success (*b* = 0.07, SE = 0.12, *P* = 0.550). One potential explanation is that challenging status quo plays a much more salient role in developing utility patents compared with design and plant patents, as utility patents are granted for new discoveries and inventions of technology and products, which require high levels of inventiveness (71). In contrast, design patents are for new designs of existing products and plant patents are for creation and reproduction of a new plant variety, involving more incremental innovation (2, 72, 73).

**Table 9.** Links between gender bias in cultural tightness and gender inequality in innovation (patent inventors and STEM occupations).

Variables	Gender inequality in patent inventors: utility^a^		Gender inequality in patent inventors: design		Gender inequality in patent inventors: plant		Gender inequality in patent inventors (aggregate of design, plant, and utility patents)		Gender inequality in STEM occupations	
	Model 1	Model 2	Model 3	Model 4	Model 5	Model 6	Model 7	Model 8	Model 9	Model 10
Gender bias in cultural tightness	0.01*** (0.**00)**	0.01*** (0.**00)**	**−0.01** **(0**.**01)**	**−0.01** **(0**.**01)**	**−0.01** **(0**.**08)**	**0.07** **(0**.**12)**	0.01*** (0.**00)**	0.01** (0.**00)**	0.01** (0.**00)**	0.01** (0.**00)**
GDP per capita (log)		−0.07*** (0.01)		−0.25*** (0.07)		1.05^+^ (0.54)		−0.10*** (0.01)		−0.03 (0.02)
Gender imbalance in population (more men than women)		0.23** (0.08)		−0.43 (0.38)		5.68 (3.62)		0.25* (0.10)		0.45*** (0.13)
State cultural tightness		−0.00* (0.00)		−0.00* (0.00)		0.01 (0.01)		−0.00** (0.00)		−0.00 (0.00)
Constant	0.85*** (0.01)	1.68*** (0.15)	0.85*** (0.04)	3.66*** (0.76)	0.36 (0.28)	−11.51^+^ (6.10)	0.85*** (0.01)	2.00*** (0.17)	0.63*** (0.02)	0.97*** (0.22)
Year	2008–2020	2008–2020	2008–2020	2008–2020	2008–2020	2008–2020	2008–2020	2008–2020	2005–2019	2005–2019
*N*	650	650	626	626	187	187	650	650	750	750

^+^ *P* < 0.1; ^*^P < 0.05; ^**^P < 0.01; ^***^P < 0.001. Results from hierarchical linear modeling. Unstandardized regression coefficients are reported. Standard errors in parentheses. The bold values are coefficients of the relationships between the focal variables and gender bias in cultural tightness. ^a^Gender inequality in patent inventors was obtained and computed from the PatentsView reported by the U.S. Patent and Trademark Office (USPTO; 2008–2020), while gender inequality in STEM was obtained and computed from the U.S. Census Bureau's American Community Survey (ACS; 2005–2019).

Since most utility patents are based in STEM fields (8), we also examined the relationship between gender bias in cultural tightness and gender inequality in STEM occupations, which was computed by using the equation mentioned above with the data from the U.S. Census Bureau's ACS (2005–2019). As shown in Table 9, they were indeed positively related (*b* = 0.01, SE = 0.005, *P* = 0.003). That is, states that place more constraints on women than men also have fewer women in STEM occupations.

Similarly, to the extent that education attainment is necessary for innovation and doctoral degrees involve original knowledge creation (especially for utility innovation) (74–76), we examined the relationship between gender bias in cultural tightness and gender inequality in higher education attainment. As shown in Table 10, gender bias in cultural tightness was positively related to gender inequality in attaining a doctorate degree (*b* = 0.01, SE = 0.002, *P <* 0.001) but not significantly related to gender inequality in attaining a bachelor's degree (*b* = −0.0002, SE = 0.01, *P* = 0.871), a master's degree (*b* = 0.01, SE = 0.03, *P* = 0.625), or a professional degree (*b* = 0.04, SE = 0.02, *P* = 0.107). In sum, our results showed that states with tighter cultural constraints on women (compared with men) have fewer number of women patent holders, fewer women in STEM occupations, and fewer women with doctorate degrees.

**Table 10.** Links between gender bias in cultural tightness and gender inequality in higher education attainment.

Variables	Gender inequality in attainment of bachelor's degrees^a^		Gender inequality in attainment of master's degrees		Gender inequality in attainment of professional degrees		Gender inequality in attainment of doctoral degrees	
	Model 1	Model 2	Model 3	Model 4	Model 5	Model 6	Model 7	Model 8
Gender bias in cultural tightness	**−0.01** **(0**.**01)**	**−0.00** **(0**.**01)**	**−0.03** **(0**.**03)**	**0.01** **(0**.**03)**	**0.01** **(0**.**02)**	**0.04** **(0**.**02)**	0.01** (0.**00)**	0.01*** (0.**00)**
GDP per capita (log)		0.02 (0.05)		−0.17 (0.12)		0.09 (0.11)		−0.00 (0.01)
Gender imbalance in population (more men than women)		0.19 (0.30)		0.92 (0.74)		1.11^+^ (0.59)		0.17*** (0.05)
State cultural tightness		−0.00 (0.00)		−0.01** (0.00)		−0.00 (0.00)		−0.00 (0.00)
Constant	−0.12*** (0.03)	−0.32 (0.55)	−0.30*** (0.09)	1.81 (1.30)	0.12 (0.08)	−0.84 (1.19)	0.91*** (0.01)	0.95*** (0.09)
Year	2003–2020	2003–2020	2003–2020	2003–2020	2003–2020	2003–2020	2003–2020	2003–2020
*N*	900	900	900	900	898	898	898	898

^+^ *P* < 0.1; ^*^P < 0.05; ^**^P < 0.01; ^***^P < 0.001. Results from hierarchical linear modeling. Unstandardized regression coefficients are reported. Standard errors in parentheses. The bold values are coefficients of the relationships between the focal variables and gender bias in cultural tightness. ^a^Gender inequality in higher education was obtained and computed from the Current Population Survey (CPS; 2003–2020) reported by the U.S. Census Bureau.

### Additional analyses on gender inequality in entrepreneurship

We also examined the relationships between gender bias in cultural tightness and gender inequality in entrepreneurship as additional evidence on the robustness of the relationships between gender bias in cultural tightness and gender inequality in innovation, given that entrepreneurship often involves disruptive innovation that breaks existing industry rules (77, 78). That is, entrepreneurs are individuals who reform or revolutionize current patterns of production by creating new products, services, and processes (79), and only by breaking rules rather than accepting conventional wisdom can entrepreneurs embrace emerging business opportunities (80–83). Accordingly, we collected data on the ownership of startup firms from the Annual Survey of Entrepreneurs (ASE; 2014–2016) and computed gender inequality among the owners of those firms. As a firm-level survey with a focus on young firms and the experiences of firm owners (i.e. entrepreneurs), the ASE collected information annually on up to 4 owners from a sample of about 290,000 firms with paid employees over the entire private nonagricultural US economy (84). Table S6 showed that gender bias in cultural tightness was positively related to gender inequality in entrepreneurship (i.e. number of startup firms owned by women versus men) (*b* = 0.03, SE = 0.01, *P <* 0.001). To the extent that entrepreneurship is a main path to firm ownership (85) as people leave wage-based employment to start their own businesses (86), we also examined the relationship between gender bias in cultural tightness and gender inequality in firm ownership of all types of firms (Survey of Business Owners [SBO; 2002–2012] from the U.S. Census Bureau) and found that they were also positively related (*b* = 0.02, SE = 0.01, *P* = 0.009). Taken together, our findings suggest that fewer women become entrepreneurs in states where women are more constrained by cultural norms than men, which is consistent with our theory that states with tighter cultural constraints on women (compared with men) have lower levels of innovation.

### Additional analyses on three gender equality scores

To further empirically differentiate gender bias in cultural tightness and three gender equality scores, we first conducted a series of analyses to test the relationships between the three gender equality scores and sociopolitical factors and gender-related threats. The results (see summary in Table S7) indicated that the overall relationships between gender bias in cultural tightness and sociopolitical factors and gender-related threats (percentage of supported propositions = 81.0%) appeared slightly stronger than the relationships between the three gender equality scores and such factors (for state gender parity index, percentage of supported propositions = 76.2%; for state gender equality score, percentage of supported propositions = 76.2%; and for state municipal equality index, percentage of supported propositions = 76.2%). Furthermore, we conducted a series of analyses to test the relationships between the three gender equality scores and gender inequality in leadership and innovation, in which gender bias in cultural tightness and the three gender equality scores were included *separately* into HLMs to predict gender inequality in leadership and innovation. The results (see summary in Table S8) indicated that the strengths of the relationships between the three gender equality scores and gender inequality in leadership and innovation were pretty low (for state gender parity index, percentage of supported propositions = 21.1%; for state gender equality score, percentage of supported propositions = 42.1%; and for state municipal equality index, percentage of supported propositions = 0.0%) and significantly weaker than the strength of the relationships between gender bias in cultural tightness and gender inequality in leadership and innovation (percentage of supported propositions = 84.2%).

For further verification, we conducted a series of analyses to investigate the incremental validity of gender bias in cultural tightness with regard to its effects on gender inequality in leadership and innovation above and beyond the effects of the three gender equality scores (see summary in Table S9). The results of our analyses, in which gender bias in cultural tightness and the three gender equality scores were included *together* into HLMs to predict gender inequality in leadership and innovation, indicated that the effects of gender bias in cultural tightness on gender inequality in leadership and innovation remained significant after controlling for the three gender equality scores (with an exception, its effect on gender inequality in boards of publicly traded companies became marginally significant: *b* = 0.03, SE = 0.01, *P* = 0.074) and most of the three gender equality scores’ effects became nonsignificant (for state gender parity index, percentage of supported propositions = 15.8%; for state gender equality score, percentage of supported propositions = 21.1%; and for state municipal equality index, percentage of supported propositions = 0.0%).

In addition, when gender bias in cultural tightness had significant effects on the outcomes in our main analyses, we further tested whether there were significant differences between the effect sizes of gender bias in cultural tightness and the three gender equality scores, respectively. The results (see summary in Table S9) indicated that gender bias in cultural tightness had significantly stronger effect sizes than the three gender equality scores in most of the propositions (i.e. effect sizes stronger than that of state gender parity index in 93.8% of propositions, effect sizes stronger than that of state gender equality score in 81.3% of propositions, and effect sizes stronger than that of state municipal equality index in 93.8% of propositions).

Overall, although the three gender equality scores appeared related to gender bias in cultural tightness, they had significantly weaker associations with gender inequality in leadership and innovation. We interpret these findings as evidence that gender bias in cultural tightness and the three gender equality scores as residing in different nomological nets.^2^ Importantly, we also find evidence that gender bias in cultural tightness was still related to gender inequality in leadership and innovation above and beyond the effects of the three gender equality scores and the effect sizes were generally stronger than those of the three gender equality scores.

## Discussion

This research reveals that, in general, women are more constrained by cultural norms than men in the United States and that there is significant variation in gender bias in cultural tightness across the 50 states. Such variability appears to be associated with sociopolitical factors (religion and political ideology) and gender-related threats. Importantly, we found that gender bias in cultural tightness is associated with gender inequality (favoring men) in business and political leadership and innovation at the state level.

This research makes several theoretical contributions to the literatures on cultural tightness and gender inequality. First, it contributes to the cultural tightness theory by offering new insights into whether a given society's cultural norms apply equally to men and women. Prior research has documented that there is wide variability in tightness across nations, states, and provinces (1, 2, 4). However, this earlier research did not investigate whether the extent of cultural tightness is the same for both men and women in a given nation or region. We argue that gender bias in cultural tightness exists across societies. We test this thesis with data from the US 50 states and found that even within the same state, there may be different degrees of normative constraints and tolerance of aberrant behaviors for women versus men (i.e. gender bias in cultural tightness). Our findings highlight that cultural tightness may not be applied equally to every individual within a given society. In particular, some societies appear to place more constraints on women than on men.

Second, this research sheds further light upon how such gender bias in cultural tightness is potentially formed in a given state. Our findings revealed that gender bias in cultural tightness appears to be associated with a variety of sociopolitical factors and gender-related threats. For example, people in states that highly value religion and have more residents holding certain religious beliefs (e.g. Mormonism) are more likely to endorse traditional gender roles; these attitudes are associated with greater gender bias in cultural tightness. Interestingly, not all religions are associated with greater constraints on women (e.g. negative association, Catholic and Jews; nonsignificant association, Protestants and other Christian), suggesting the specific religious doctrines matter. Additionally, political ideology (e.g. political conservatism) and gender-related social threats (e.g. sexism and men overrepresentation) were positively associated with gender bias in cultural tightness.

Third, our research demonstrates that gender bias in cultural tightness has important implications on gender inequality in leadership and innovation. Greater involvements and achievements in leadership and innovation among men than women have been consistently found in many economies (8, 12). Researchers have increasingly focused on understanding the gender inequality in these domains. One stream of research examines differences in individual characteristics between men and women (e.g. personality, intelligence, and risk preferences) and how they are associated with gender inequality in leadership and innovation (8, 89, 90). Another stream of work investigates economic factors (e.g. difference in the pay of men and women), institutional and political factors (e.g. differences in social security and accessibility of finance or resources for men and women), and social factors (e.g. prejudices, preconceptions, and stereotypes about women) (8, 89, 91). The role of local cultural norms in shaping gender inequality in leadership and innovation, however, has been largely neglected. By examining the relationships between gender bias in cultural tightness and gender inequality in leadership and innovation, our research offers an additional account to help better understand gender inequality in society from a cultural perspective. For example, researchers have noted inequality in innovation achievement between men and women (2, 92). To the extent that culture is associated with creativity and innovation (2), the present work highlights how a greater degree of constraints imposed on women versus men could partially explain the innovation challenges they face.

As with all research, the current investigation has some limitations. First, the data were collected online via MTurk. To unpack this concern, we conducted a series of analyses on sample representativeness. We first conducted HLMs to examine if the gender distribution (0, women; 1, men), average age (in years), and average education level (0, lower than a bachelor's degree; 1, equal to or higher than a bachelor's degree) of the general population within the state (from the U.S. Census Bureau [2020]) are associated with our sample's gender, age, and education level. Results showed that gender distribution and average education level of the general population within the state were positively and significantly associated with our sample's gender and education level (for gender: *b* = 8.74, SE = 2.71, *P* = 0.001; for education level: *b* = 0.00005, SE = 0.00005, *P* = 0.029); however, the average age of the general population within the state was positively but not significantly associated with our sample's age (*b* = 0.14, SE = 0.09, *P* = 0.102). These results suggested that our sample's demographics were generally positively associated with population demographics within the state. Furthermore, we used *t*-tests to compare our sample's gender, age, and education level with those of the general population within the state, respectively (see Table S10 for the descriptive summary). In most of the states (i.e. 45 states in 50 states), our sample's age was lower than the average age of the general population within the state, and our sample's education level was higher than the average education level of the general population within the state for all 50 states. In some states (i.e. 32 states, such as Colorado, Main, and Michigan), our sample's men ratio (i.e. the number of men/[the number of men + the number of women]) was significantly lower than the men ratio of the general population within the state, whereas in other states (i.e. 18 states, such as California, Illinois, and Texas), our sample's men ratio was not significantly different from the men ratio of the general population within the state. Overall, our samples were younger and more educated compared with the general population within the state, and our samples from some states were more representative than others. However, additional analyses on sample characteristics revealed that any deviations between our sample and the general population in terms of gender, age, and education level were not materially associated with gender bias in cultural tightness (for the difference in gender: *b* = −3.15, SE = 3.74, *P* = 0.404; for the difference in age: *b* = 0.06, SE = 0.06, *P* = 0.357; for the difference in education level: *b* = 1.83, SE = 1.81, *P* = 0.316; see Supplementary Text and Table S11). Nevertheless, future research could use random sampling methods with more representative samples to further test the generalizability of our findings.

Second, although we measured gender bias in cultural tightness over two waves of survey 1 year and 2 months apart and found that gender bias in cultural tightness at the state level are relatively stable over time (i.e. our results showed good test–retest reliability of gender bias in cultural tightness at the state level and that the data collected from the two waves had comparable associations with sociopolitical factors and gender-related threats, as well as gender inequality in leadership and innovation), it is still unclear whether these differences across states would change if societal mores and norms change over longer time periods. Thus, future research could use our measures to track how gender bias in cultural tightness evolves over longer time periods such as over the next 5 to 10 years.

Third, as with all cross-sectional research, we were unable to unequivocally ascertain causality among the key variables. We acknowledge that it is plausible that both gender bias in cultural tightness and gender inequality outcomes are driven by the same factors (e.g. religious beliefs or political ideologies). To test this possibility, we conducted additional analyses and found that when the two main types of potential antecedent variables (i.e. sociopolitical factors and gender-related threats) were included in the same model as gender bias in cultural tightness, gender bias in cultural tightness still exerted statistically significant effects on most outcomes of interests (i.e. gender inequality in [business and political] leadership and innovation). These findings highlight the unique contribution of gender bias in cultural tightness beyond those related factors and partially assuage concerns that some third factors are simultaneously driving the variances in gender bias in cultural tightness and gender inequality in leadership and innovation. Nevertheless, future research may explore novel methods to establish causality and replicate our findings.

Finally, this research focused on the gender bias in cultural tightness at the state level. Future work could examine national-level gender bias in cultural tightness, as differences in gender bias in cultural tightness likely exist across nations given different religious and political environments. Furthermore, although we found that women are overall more constrained by cultural norms than men across a variety of domains in their work and life, precarious manhood theory suggests that men (compared with women) are more restricted by some specific norms regarding agency and dominance (20, 21, 93). Our theorizing and precarious manhood theory coexist because they cover different scopes of norms—gender bias in cultural tightness covers all aspects of norms, whereas precarious manhood covers only specific aspects of norms related to the protection of manhood status. Nevertheless, future research could investigate conditions under which men are more constrained, and thus face tighter cultural norms, than women.

These limitations notwithstanding, the current work represents a step forward in advancing the theory of cultural tightness. By recognizing that cultural constraints are applied differently on men and women within the same society and that such differences are associated with outcomes such as gender inequality in leadership and innovation, we set the stage for future inquiries to investigate the implications of cultural tightness with more nuanced lenses and levels of analyses.

## Supplementary Material

pgad238_Supplementary_Data

## Data Availability

All data are available in https://osf.io/wqtnh/.

## Footnotes

1 We acknowledge that gender bias in cultural tightness and the three gender equality scores are conceptually similar in that they are all related to gender inequality. Despite the similarity, they are also conceptually different. The key difference is that they represent different aspects of gender inequality. We conceptualized gender bias in cultural tightness as a cultural dynamic that measures the extent to which norms regarding permissible behaviors and tolerance of aberrant behaviors toward women are tighter than toward men. This construct does not specify any specific tangible outcomes that women and men may receive. As such, gender bias in cultural tightness may very well be detrimental or beneficial to both women and men. Conversely, the gender equality scores measure specific tangible gender gap outcomes in a variety of domains (e.g., employment, workplace environment, education, civil rights, and health conditions). Additionally, gender bias in cultural tightness focuses squarely on differences in norms dynamics that women and men face, whereas the three gender equality scores are more about fair access to a broad range of opportunities and resources between the genders. Gender bias in cultural tightness could very well lead to inequitable access to opportunities and resources. However, inequitable access to opportunities and resources could have other antecedents beyond gender bias in cultural tightness (e.g., gender stereotypes, job demands, laws, and policies).

2 Nomological network refers to “a system of scientific laws that relates constructs to each other and to observations” (87). Different nomological nets mean that the compared constructs have different relationships with other variables (e.g. outcomes) (88). For example, in our study, gender bias in cultural tightness was associated with gender inequality in US Senators, but the three gender equality scores were not associated with this outcome, suggesting that gender bias in cultural tightness and the three gender equality scores reside in different nomological nets.
