# Supplementary material for: Gender bias in cultural tightness across the 50 US states, its correlates, and links to gender inequality in leadership and innovation
Source: PNAS Nexus. 2023 Jul 20;2(8):pgad238. doi: 10.1093/pnasnexus/pgad238 (PMC10396071; doi:10.1093/pnasnexus/pgad238)
Supplement: pgad238_Supplementary_Data [file pgad238_supplementary_data.docx]

Supplementary Materials for

**Gender Bias in Cultural Tightness Across the 50 U.S. States, Its Correlates, and Links to Gender Inequality in Leadership and Innovation**

**Authors:**

Xin Qin^1^, Roy Y. J. Chua^2*^, Ling Tan^3^, Wanlu Li^1^, Chen Chen^1*^

Correspondence to: Roy Y. J. Chua: royyjchua@smu.edu.sg; Chen Chen: chench28@mail.sysu.edu.cn.

**This PDF file includes:**

Supplementary Text

Tables S1 to S33

Additional references not in the main text

**Supplementary Text**

**Sample and Procedures**

Participants were recruited from Amazon Mechanical Turk (MTurk) in the U.S. via two waves of data collection from March to June 2020, and from August to December 2021. To ensure the authenticity and validity of our data, we restricted our participants to the individuals who met the following criteria: (i) at least 18 years old, U.S. citizens; (ii) must be current residents of the U.S. who are born and currently live within the same state; (iii) each participant can only participate once (i.e., their IP addresses needs to be unique). Each participant was compensated about 0.60 USD for their time.

In addition to demographic information, participants completed the following six-item scale regarding gender bias in cultural tightness (1 = “Strongly disagree,” 6 = “Strongly agree”):

1. There are many more social norms that women (compared with men) are supposed to abide by in this state.
2. In this state, there are much clearer expectations for how women (compared with men) should act in most situations.
3. People agree upon much more what behaviors for women (compared with for men) are appropriate versus inappropriate in most situations in this state.
4. Women (compared with men) in this state have much more freedom in deciding how they want to behave in most situations. (reversely coded)
5. In this state, if a woman (compared with a man) acts in an inappropriate way, others will much more strongly disapprove.
6. Women (compared with men) in this state much more comply with social norms.

Because non-locals (i.e., individuals who were born out-of-state but have lived within the given state) may have been imprinted by different cultures, languages, and religions that would confound our results, we only included locals (individuals who are born and currently live within the same state) in our following analyses. The final sample includes 15,425 participants in total and 309 participants on average (ranging from 295 to 336) for each state. Of 15,425 participants, 57.31% were women; 61.63% received a bachelor’s or higher degree; mean age was 35.94 years, ranging from 18 to 70 years. Participants were from a broad range of social groups, including professionals (e.g., sales personnel, accountants, doctors, R&D staffs, managers, and teachers), students, and others.

**Preliminary Analyses**

To check whether the two waves of data can be merged for further analyses, we conducted several tests to compare mean differences between the first wave (i.e., from March 2020 to June 2020) and the second wave (i.e., from August 2021 to December 2021). We first examined mean differences of three demographical variables: gender (0 = women; 1 = men), age (in years), and education level (0 = lower than a bachelor’s degree; 1 = equal to or higher than a bachelor’s degree). The results showed that there were no differences in gender (*mean difference* = -0.003, *SE* = 0.01, *t*(49) = -0.32, *P* = 0.753), age (*mean difference* = 0.51, *SE* = 0.36, *t*(49) = 1.41, *P* = 0.164), and education level (*mean difference* = 0.0006, *SE* = 0.01, *t*(49) = 0.04, *P* = 0.965) between the two waves of participants.

Next, we compared the two waves’ gender bias in cultural tightness and found that the second wave’s gender bias in cultural tightness was significantly higher than the first wave’s (*mean difference* = 0.09, *SE* = 0.01, *t*(49) = 6.15, *P* < 0.001). One interpretation for this difference is that the COVID-19 pandemic’s different implications for men and women in the U.S. might have over time placed greater constraints on women than men (e.g., women’s increased housework during the COVID-19 pandemic have restricted them to traditional gender roles) (1-3).

To check whether this difference in gender bias in cultural tightness between these two waves of data has implications for our key variables, we conducted the following analyses. First, we analyzed the correlation between the two waves of data and found that gender bias in cultural tightness at the state level between the two waves was highly correlated (*r*_[48]_ = 0.86, *P* < 0.001, *n* = 50). Second, we calculated the extent of changes in overall relative ranking of gender bias in cultural tightness among states from Wave 1 to Wave 2. The results showed that 84% of states’ relative ranking remains unchanged, indicating that most of states’ relative rankings of gender bias in cultural tightness are stable. Third, we analyzed the associations between gender bias in cultural tightness and the key variables (i.e., socio-political factors and gender-related threats, as well as gender inequality in leadership and innovation) by using data collected in Wave 1 and Wave 2 separately.

Specifically, we first analyzed the associations between socio-political factors and gender-related threats and gender bias in cultural tightness by using data collected in Wave 1 and Wave 2 separately. The results showed that most of the results were comparable in terms of coefficients and significant levels between the two waves of data. There were however several exceptions: (a) the percentage of Catholics (all data reported in the main text: *b* = -3.76, *SE* = 1.69, *P =* 0.031; Wave 1: *b* = -4.13, *SE* = 1.72, *P* = 0.020; Wave 2: *b* = -3.27, *SE* = 1.72, *P* = 0.064), (b) the percentage of Black Protestants (all data reported in the main text: *b* = 4.67, *SE* = 2.49, *P =* 0.067; Wave 1: *b* = 4.02, *SE* = 2.57, *P* = 0.125; Wave 2: *b* = 4.90, *SE* = 2.49, *P* = 0.055), and (c) the percentage of people favoring non-discrimination LGBT protection (all data reported in the main text: *b* = -6.67, *SE* = 3.16, *P =* 0.040; Wave 1: *b* = -5.42, *SE* = 3.29, *P* = 0.106; Wave 2: *b* = -7.09, *SE* = 3.16, *P* = 0.030), see Tables S12-S21. It is worth noting that these differences largely pertained to the level of statistical significance of a given effect; the directions of these effects were consistent across both waves of data.

We next analyzed the associations between the leadership and innovation variables and gender bias in cultural tightness by using data collected in Wave 1 and Wave 2 separately. The results showed that most of the results were comparable in terms of coefficients and significant levels between the two waves of data. There were however a few exceptions: (a) gender inequality in CEOs of publicly traded companies (all data reported in the main text: *b* = 0.04, *SE* = 0.02, *P* = 0.046; Wave 1: *b* = 0.03, *SE* = 0.02, *P* = 0.134; Wave 2: *b* = 0.04, *SE* = 0.02, *P* = 0.041), (b) U.S. Senators (all data reported in the main text: *b* = 0.07, *SE* = 0.03, *P =* 0.017; Wave 1: *b* = 0.07, *SE* = 0.03, *P* = 0.016; Wave 2: *b* = 0.06, *SE* = 0.03, *P* = 0.052), (c) State Governors (all data reported in the main text: *b* = 0.75, *SE* = 0.37, *P =* 0.043; Wave 1: *b* = 0.73, *SE* = 0.35, *P* = 0.038; Wave 2: *b* = 0.69, *SE* = 0.38, *P* = 0.067), and (d) attainment of professional degrees (all data reported in the main text: *b* = 0.04, *SE* = 0.02, *P* = 0.107; Wave 1: *b* = 0.04, *SE* = 0.02, *P* = 0.066; Wave 2: *b* = 0.03, *SE* = 0.02, *P* = 0.187), see Tables S22-S31. Again, these differences largely pertained to the level of statistical significance of a given effect; the directions of these effects were consistent across both waves of data.

Overall, these results showed good test-retest reliability of gender bias in cultural tightness at the state level and that the data collected from the two waves had comparable associations with socio-political factors and gender-related threats, as well as gender inequality in leadership and innovation. As such, we used the combined data from the two waves of data collection for further analyses.

We also conducted an OLS regression analysis to test if our sample’s demographics at the individual level (i.e., gender, age, education level, and socioeconomic status) are associated with gender bias in cultural tightness perceptions. Specifically, we included the following variables simultaneously in the model: gender (0 = women; 1 = men), age (in years), education level (0 = lower than a bachelor’s degree; 1 = equal to or higher than a bachelor’s degree), and socioeconomic status (annual family income: 1 = lower than or equal to $30,000; 2 = $30,001-$60,000; 3 = $60,001-$90,000; 4 = $90,001-$120,000; 5 = higher than $120,000), and a fixed effect of participants’ state. Our results (Table S32) revealed that gender was significantly related to gender bias in cultural tightness perceptions (*b* = -0.08, *SE* = 0.01, *P* < 0.001) such that women reported greater bias in cultural tightness perceptions than men. It thus appears that individuals who are more constrained by society also experience the bias in constraints more acutely. However, age (*b* = -0.00004, *SE* = 0.001, *P* = 0.945), education level (*b* = 0.01, *SE* = 0.01, *P* = 0.462), and socioeconomic status (*b* = -0.01, *SE* = 0.01, *P* = 0.212) were not significantly related to gender bias in cultural tightness perceptions.

Given that women reported greater bias in cultural tightness perceptions than men, we conducted two supplementary sets of analyses to see how this finding affects our overall thesis. First, we conducted additional analyses by including state gender ratio in the data as a control variable and found that the results were highly similar compared with those reported in the main text. Second, we analyzed the associations between gender bias in cultural tightness and the key variables (i.e., socio-political factors and gender-related threats, as well as gender inequality in leadership and innovation) by using data reported by women and men separately. Results reported below indicate that findings from women and men data are highly comparable (to keep the Supplementary Materials to a manageable length, we did not include the full table of these results here. A full report of these results is available from the authors upon request).^[[1]](#footnote-1)^

We first analyzed the associations between socio-political factors and gender-related threats and gender bias in cultural tightness by using data reported by women and men separately. We found that the results were highly comparable in terms of coefficients and significant levels between the two sets of data (there were two exceptions: (a) the percentage of Catholics [all data reported in the main text: *b* = -3.76, *SE* = 1.69, *P =* 0.031; women data: *b* = -2.92, *SE* = 1.71, *P* = 0.095; men data: *b* = -3.99, *SE* = 1.75, *P* = 0.028], (b) the percentage of Black Protestants [all data reported in the main text: *b* = 4.67, *SE* = 2.49, *P =* 0.067; women data: *b* = 2.21, *SE* = 2.54, *P* = 0.389; men data: *b* = 6.77, *SE* = 2.49, *P* = 0.009]).

We next analyzed the associations between gender inequality in leadership and innovation and gender bias in cultural tightness by using data reported by women and men separately. Again, we found that the results were highly comparable in terms of coefficients and significant levels between the two sets of data. There were several exceptions: (a) gender inequality in CEOs of publicly traded companies (all data reported in the main text: *b* = 0.04, *SE* = 0.02, *P* = 0.046; women data: *b* = 0.04, *SE* = 0.02, *P* = 0.038; men data: *b* = 0.03, *SE* = 0.02, *P* = 0.126), (b) gender inequality in management occupations: total (all data reported in the main text: *b* = 0.03, *SE* = 0.01, *P =* 0.002; women data: *b* = 0.04, *SE* = 0.01, *P* < 0.001; men data: *b* = 0.02, *SE* = 0.01, *P* = 0.082), (c) gender inequality in U.S. Senators (all data reported in the main text: *b* = 0.07, *SE* = 0.03, *P =* 0.017; women data: *b* = 0.08, *SE* = 0.03, *P* = 0.005; men data: *b* = 0.05, *SE* = 0.03, *P* = 0.118), (d) gender inequality in state governors (all data reported in the main text: *b* = 0.75, *SE* = 0.37, *P =* 0.043; women data: *b* = 0.66, *SE* = 0.36, *P* = 0.069; men data: *b* = 0.62, *SE* = 0.36, *P* = 0.085), and (e) gender inequality in patent inventors (aggregate of design, plant, and utility patents) (all data reported in the main text: *b* = 0.01, *SE* = 0.004, *P =* 0.004; women data: *b* = 0.01, *SE* = 0.004, *P* < 0.001; men data: *b* = 0.01, *SE* = 0.004, *P* = 0.072). Despite these few differences in the level of statistical significance in the results for men versus women, the directions of the effects were all consistent. Overall, both data reported by women and men had comparable associations with socio-political factors and gender-related threats, as well as gender inequality in leadership and innovation.

Next, we conducted another OLS regression analysis to examine if differences between our sample’s demographics from those of the general population are associated with gender bias in cultural tightness. Specifically, we collected the average gender, age, and education level of each state from the U.S. Census Bureau (2020), and subsequently computed the differences by subtracting our sample’s average gender, age, and education level at the state level from those of the general population within the state (socioeconomic status was not included because state-level data of the general population on this variable was not available). The results (Table S11) demonstrated that differences in gender (*b* = -3.15, *SE* = 3.74, *P* = 0.404), age (*b* = 0.06, *SE* = 0.06, *P* = 0.357), and education level (*b* = 1.83, *SE* = 1.81, *P* = 0.316) were not significantly related to gender bias in cultural tightness at the state level.^[[2]](#footnote-2)^

**Divergent and Convergent Validity Analyses**

To check the divergent validity of gender bias in cultural tightness, we first tested whether state-level gender bias in cultural tightness was distinct from state-level gender-related constructs. To measure state-level gender equality, we collected three gender equality scores including (i) state gender parity index from the RepresentWomen’s Gender Parity Index 2019 Report (5), (ii) state gender equality score from the WalletHub 2020’s Best and Worst States for Women’s Equality Report (6), and (iii) state municipal equality index from the Human Rights Campaign Foundation and the Equality Federation Institute in 2020 (7). The state gender parity index represents women’s recent electoral success at the local, state, and national level. The scale ranges from 0 (if no women were elected to any offices) to 100 (if women held all elected offices) points. A higher score means there is more women’s representation in politics. For state gender equality score from the WalletHub, it is evaluated by considering three dimensions (i.e., workplace environment, education and health, and political empowerment) with their corresponding weights. A higher score represents a more favorable condition for women equality. For the state municipal equality index, the score is based on non-discrimination laws, municipal employment, municipal services, law enforcement and leadership on LGBTQ equality. A higher score indicates more inclusive municipal laws and policies, as well as services that are friendly toward LGBTQ people in each state.

We calculated gendered housework disparity ratio—the ratio of women’s mean minutes to men’s mean minutes in doing routine housework in each state (8). We used this ratio as a proxy of state masculinity. A greater ratio represents higher masculinity because it means women are doing more housework and spending more time with their family than men.

We measured state collectivism using the United States collectivism index developed by Vandell and Cohen (1999) (9). This index was measured by eight items that related to family structure and living arrangements (i.e., percentage of people living alone, percentage of elderly people (aged 65+ years) living alone, and percentage of households with grandchildren in them), and other social (i.e., divorce to marriage ratio), political (i.e., average percentage voting Libertarian over the last four presidential elections), religious (i.e., percentage of people with no religious affiliation), and economic (i.e., ratio of people carpooling to work to people driving alone and percentage of self-employed workers) practices (9). A higher score indicates greater collectivism.

Second, we tested whether gender bias in cultural tightness was distinct from general cultural tightness. We recruited an additional 1237 participants (52.38% women; 63.38% holding a bachelor’s or higher degree; *Mean*_age_ = 39.48) across the 50 U.S. states through Prolific and invited them to rate gender bias in cultural tightness scale and general cultural tightness scale developed by Gelfand and colleague (2011), respectively. We conducted a series of CFAs to test whether our measure was distinct from the general cultural tightness measure by Gelfand and colleague (2011). First, we conducted CFAs for gender bias in cultural tightness and general cultural tightness at the individual level. Results suggested that the theorized two-factor model (*χ*^2^ = 560.59, *P* < 0.001, *df* = 53; CFI = 0.93, TLI = 0.915, RMSEA = 0.09, SRMR = 0.06) fit the data better than the single-factor model (*χ*^2^ = 2503.16, *P* < 0.001, *df* = 54; CFI = 0.67, TLI = 0.60, RMSEA = 0.19, SRMR = 0.13). Next, we conducted multi-level CFAs. Results suggested that the theorized two-factor model (*χ*^2^ = 795.01, *P* < 0.001, *df* = 106; CFI = 0.92, TLI = 0.90, RMSEA = 0.07, SRMR_[within]_ = 0.06) fit the data better than the single-factor model (*χ*^2^ = 3382.53, *P* < 0.001, *df* = 108; CFI = 0.62, TLI = 0.54, RMSEA = 0.16, SRMR_[within]_ = 0.14). Overall, these results indicated that gender bias in cultural tightness is distinct from general cultural tightness at both individual and state levels of analyses.

As an additional convergent validity test, we explored the relationship between gender bias in cultural tightness and gender difference in rule violations. If a society places more constraints on women than men, then women are more likely than men to comply with rules. We collected and computed gender difference in using seat belts when driving (i.e., the frequency of men in using seat belts when driving minus the frequency of women in using seat belts when driving) and gender difference in driving after drinking (i.e., the number of times that men drove after drinking minus the number of times that women drove after drinking) during the past 30 days from the Behavioral Risk Factor Surveillance System of the Centers for Disease Control and Prevention in 2018 (10). We found that gender bias in cultural tightness had moderately negative correlations with gender difference in using seat belts when driving (*r*_[48]_ = -0.27, *P* = 0.058, *n* = 50) but had moderately positive correlations with gender difference in driving after drinking (*r*_[48]_ = 0.32, *P* = 0.026, *n* = 50).

**Additional Analyses on Regional or Divisional Differences in Gender Bias in Cultural Tightness Perceptions**

To further check whether such regional or divisional differences in gender bias in cultural tightness perceptions are associated with individual level factors, we conducted a series of analyses based on the individual level data and controlled for individuals’ gender (0 = women; 1 = men), age (in years), education level (0 = lower than a bachelor’s degree; 1 = equal to or higher than a bachelor’s degree), and social economic status (annual family income: 1 = lower than or equal to $30,000; 2 = $30,001-$60,000; 3 = $60,001-$90,000; 4 = $90,001-$120,000; 5 = higher than $120,000). For all the individual level analyses, we controlled for the state level effect, which took into account the nested structure of our data. The results indicated that, after controlling for these factors (for gender: *F*(1, 15416) = 32.53, *P* < 0.001, *η*^2^_p_ = 0.002; for age: *F*(1, 15416) = 0.39, *P* = 0.533, *η*^2^_p_ = 0.00003; for education level: *F*(1, 15416) = 0.37, *P* = 0.542, *η*^2^_p_ = 0.00002; for social economic status: *F*(1, 15416) = 5.55, *P* = 0.018, *η*^2^_p_ = 0.0004), there were significant differences in gender bias in cultural tightness perceptions among the four regions (*F*(3, 15416) = 49.39, *P* < 0.001, *η*^2^_p_ = 0.01), and the relative ranking of gender bias in cultural tightness perceptions among four regions were the same as that in the state level data analyses (Table S4). Similarly, after controlling for these factors (for gender: *F*(1, 15411) = 32.86, *P* < 0.001, *η*^2^_p_ = 0.002; for age: *F*(1, 15411) = 0.29, *P* = 0.588, *η*^2^_p_ = 0.00002; for education level: *F*(1, 15411) = 0.83, *P* = 0.361, *η*^2^_p_ = 0.00005; for social economic status: *F*(1, 15411) = 3.69, *P* = 0.055, *η*^2^_p_ = 0.0002), there were significant differences in gender bias in cultural tightness among the nine regional divisions (*F*(8, 15411) = 36.28, *P* < 0.001, *η*^2^_p_ = 0.02), and the relative ranking of gender bias in cultural tightness perceptions among nine regional divisions were the same as that in the state level data analyses (Table S5).

**Additional Analyses on Correlates of Gender Bias in Cultural Tightness**

Further analyses on the breakdown of key religious affiliation (Table 3) indicated that the percentage of Mormons (*b* = 3.99, *SE* = 1.63, *P =* 0.018) was positively related to gender bias in cultural tightness, the percentage of Black Protestants (*b* = 4.67, *SE* = 2.49, *P =* 0.067) was marginally related to gender bias in cultural tightness, but the percentage of Catholics (*b* = -3.76, *SE* = 1.69, *P =* 0.031), the percentage of Hindus (*b* = -56.86, *SE* = 23.54, *P =* 0.020), and the percentage of Jews (*b* = -29.57, *SE* = 11.02, *P =* 0.010) were negatively related to gender bias in cultural tightness. The percentage of Buddhists (*b* = -16.83, *SE* = 11.54, *P =* 0.151), the percentage of Evangelical Protestants (*b* = 2.10, *SE* = 1.36, *P =* 0.129), and the percentage of Mainline Protestants (*b* = -0.73, *SE* = 2.24, *P =* 0.745) were not significantly related to gender bias in cultural tightness.

**Table S1. Data sources.** The column Source presents the source from which the variables were drawn.

| **Variable** | **Source** | **Years** | **States with Missing Data (excluded from analyses)** |
| --- | --- | --- | --- |
| **Variables for Divergent Validity** | | | |
| ***(i) Gender equality*** |  |  |  |
| 1. State gender parity index | Gender Parity Index 2019 Report, RepresentWomen | 2019 | **-** |
| 1. State gender equality score | WalletHub 2020’s Best & Worst States for Women’s Equality Report | 2020 | - |
| 1. State municipal equality index | Human Rights Campaign Foundation and the Equality Federation Institute | 2020 | - |
| ***(ii) Masculinity*** |  |  |  |
| 1. Ratio of women’s mean minutes to men’s mean minutes in doing routine housework | Ruppanner & Maume | 2016 | - |
| ***(iii) Collectivism*** |  |  |  |
| 1. Collectivism | Vandello & Cohen | 1999 | - |
| ***(iv) Rule-violation/compliance*** |  |  |  |
| 1. Using seat belts when driving | Behavioral Risk Factor Surveillance System, U.S. Centers for Disease Control and Prevention (CDC) | 2018 | - |
| 1. Driving after drinking during the past 30 days | Behavioral Risk Factor Surveillance System, U.S. Centers for Disease Control and Prevention (CDC) | 2018 | - |
| **Control Variables** | | | |
| 1. GDP per capita^[[3]](#footnote-3)^ | U.S. Bureau of Economic Analysis (BEA) | 1977-2020 | - |
| 1. Gender imbalance in population | State Population by Characteristics, the U.S. Census Bureau | 1970-2020 | - |
| **Variables for Antecedents** | | | |
| ***(i) Socio-political factors: religion*** |  |  |  |
| 1. Percentage of adults who are highly religious | Religious Landscape Study, Pew Research Center | 2014 | - |
| 1. Importance of religion | Religious Landscape Study, Pew Research Center | 2014 | - |
| 1. Frequency of prayer | Religious Landscape Study, Pew Research Center | 2014 | - |
| 1. Worship attendance | Religious Landscape Study, Pew Research Center | 2014 | - |
| 1. Belief in god | Religious Landscape Study, Pew Research Center | 2014 | - |
| 1. Religious belief | Religious Landscape Study, Pew Research Center | 2014 | - |
| 1. Percentage of adults who are non-religious | Religious Landscape Study, Pew Research Center | 2014 | - |
| 1. Breakdown religious data of key religious affiliation | Religious Landscape Study, Pew Research Center | 2014 | - |
| 1. Percentage of adults who are very religious | Gallup | 2016 | - |
| 1. Percentage of adults who are moderately religious | Gallup | 2016 | - |
| 1. Percentage of adults who are non-religious | Gallup | 2016 | - |
| ***(ii) Socio-political factors: political ideology*** |  |  |  |
| 1. Percentage of people having conservative political ideology | Religious Landscape Study, Pew Research Center | 2014 | - |
| 1. Percentage of Republicans in the U.S Senate and House of Representatives | Biographical Directory of the United States Congress | 2019-2021 | - |
| ***(iii) Gender-related threats*** |  |  |  |
| 1. State sexism belief i^[[4]](#footnote-4)^ | World Values Survey (WVS) | 2017 | Alaska |
| 1. State sexism belief ii^[[5]](#footnote-5)^ | DDB Needham Life Style Survey | 1975-1998 | Alaska, Hawaii |
| 1. Percentage of people favoring non-discrimination LGBT protection | Research on LGBT, PRRI American Values Atlas | 2019 | - |
| 1. Percentage of people viewing homosexuality as acceptable | Religious Landscape Study, Pew Research Center | 2014 | - |
| 1. Percentage of male-dominated industries | Total Full-Time and Part-Time Employment by NAICS Industry, U.S. Bureau of Economic Analysis (BEA) | 2001-2018 | - |
| 1. Sexual violence against women | National Intimate Partner and Sexual Violence Survey: 2010 Summary Report, National Center for Injury Prevention and Control of the Centers for Disease Control and Prevention (CDC) | 2010 | Connecticut, Delaware, Hawaii, Kansas, Massachusetts, Mississippi, New Jersey, North Dakota, South Dakota |
| 1. Relative domestic violence | National Intimate Partner and Sexual Violence Survey: 2010 Summary Report, National Center for Injury Prevention and Control of the Centers for Disease Control and Prevention (CDC) | 2010 | Vermont |
| 1. Relative human trafficking | National Human Trafficking Hotline | 2018 | - |
| **Variables for Outcomes** | | | |
| ***(i) Leadership*** |  |  |  |
| *Business leadership* |  |  |  |
| 1. Boards and CEOs of publicly traded companies | Institutional Shareholder Services (ISS) & Compustat^[[6]](#footnote-6)^ | 2007-2020 | Vermont |
| 1. Managerial occupations: including top executives | American Community Survey (ACS) | 2005-2019 | - |
| *Political leadership* |  |  |  |
| 1. U.S. Senators | Center for American Women and Politics (CAWP) | 1901-2020 | - |
| 1. U.S. Representatives | Center for American Women and Politics (CAWP) | 1901-2020 | - |
| 1. State Senators | Center for American Women and Politics (CAWP) | 1975-2020 | - |
| 1. State Representatives^[[7]](#footnote-7)^ | Center for American Women and Politics (CAWP) | 1975-2020 | - |
| 1. State Governors^[[8]](#footnote-8)^ | Center for American Women and Politics (CAWP) | 1901-2020 | - |
| ***(ii) Innovation*** |  |  |  |
| *Patent inventors* |  |  |  |
| 1. Patent inventors^[[9]](#footnote-9)^ | PatentsView, the U.S. Patent and Trademark Office | 2008-2020 | - |
| *STEM occupations* |  |  |  |
| 1. Science, technology, engineering, and mathematics (STEM) occupations^[[10]](#footnote-10)^ | American Community Survey (ACS) | 2005-2019 | - |
| *Higher education* *attainment* |  |  |  |
| 1. Higher education attainment | Current Population Survey (CPS) | 2003-2020 | - |
| ***(iii) Entrepreneurship*** |  |  |  |
| 1. Number of startup firms | Annual Survey of Entrepreneurs (ASE) | 2014-2016 | - |
| 1. Firm ownership of all types of firms | Survey of Business Owners (SBO), the U.S. Census Bureau | 2002-2012 | - |

**Table S2. Gender bias in cultural tightness scores (at the state level) for the four U.S. census regions.**

| Region | N | Mean | Standard Deviation | 95% Confidence Interval |
| --- | --- | --- | --- | --- |
| 1. Northeast^*^ | 9 | 2.46 | 0.42 | 2.14–2.78 |
| 2. Midwest^†^ | 12 | 3.27 | 0.66 | 2.85–3.69 |
| 3. South^‡^ | 16 | 3.44 | 0.93 | 2.95–3.94 |
| 4. West^§^ | 13 | 2.58 | 1.32 | 1.78–3.38 |

*Note.* Higher score indicates greater gender bias in cultural tightness.

^*^ Connecticut, Maine, Massachusetts, New Hampshire, New Jersey, New York, Pennsylvania, Rhode Island, and Vermont.

^†^ Illinois, Indiana, Iowa, Kansas, Michigan, Minnesota, Missouri, Nebraska, North Dakota, Ohio, South Dakota, and Wisconsin.

^‡^ Alabama, Arkansas, Delaware, Florida, Georgia, Kentucky, Louisiana, Maryland, Mississippi, North Carolina, Oklahoma, South Carolina, Tennessee, Texas, Virginia, and West Virginia.

^§^ Alaska, Arizona, California, Colorado, Hawaii, Idaho, Montana, Nevada, New Mexico, Oregon, Utah, Washington, and Wyoming.

**Table S3. Gender bias in cultural tightness scores (at the state level) for the nine U.S. census regional divisions.**

| Regional Division | N | Mean | Standard Deviation | 95% Confidence Interval |
| --- | --- | --- | --- | --- |
| 1. New England^*^ | 6 | 2.57 | 0.37 | 2.18–2.95 |
| 2. Middle Atlantic^†^ | 3 | 2.25 | 0.51 | 0.99–3.50 |
| 3. East North Central^‡^ | 5 | 3.64 | 0.80 | 2.65–4.63 |
| 4. West North Central^§^ | 7 | 3.01 | 0.43 | 2.61–3.40 |
| 5. South Atlantic^¶^ | 8 | 3.10 | 1.11 | 2.17–4.03 |
| 6. East South Central^\|\|^ | 4 | 3.97 | 0.81 | 2.68–5.26 |
| 7. West South Central^**^ | 4 | 3.60 | 0.28 | 3.16–4.04 |
| 8. Mountain^††^ | 8 | 3.08 | 1.44 | 1.87–4.28 |
| 9. Pacific^‡‡^ | 5 | 1.79 | 0.56 | 1.10–2.48 |

*Note.* Higher score indicates greater gender bias in cultural tightness.

^*^ Connecticut, Maine, Massachusetts, New Hampshire, Rhode Island, and Vermont.

^†^ New Jersey, New York, and Pennsylvania.

^‡^ Illinois, Indiana, Michigan, Ohio, and Wisconsin.

^§^ Iowa, Kansas, Minnesota, Missouri, Nebraska, North Dakota, and South Dakota.

^¶^ Delaware, Florida, Georgia, Maryland, North Carolina, South Carolina, Virginia, and West Virginia.

^||^ Alabama, Kentucky, Mississippi, and Tennessee.

^**^ Arkansas, Louisiana, Oklahoma, and Texas.

^††^ Arizona, Colorado, Idaho, Montana, Nevada, New Mexico, Utah, and Wyoming.

^‡‡^ Alaska, California, Hawaii, Oregon, and Washington.

**Table S4. Gender bias in cultural tightness perceptions (at the individual level) for the four U.S. census regions.**

| Region | N | Mean | Standard Error | 95% Confidence Interval |
| --- | --- | --- | --- | --- |
| 1. Northeast^*^ | 2775 | 3.74 | 0.02 | 3.71–3.77 |
| 2. Midwest^†^ | 3697 | 3.90 | 0.01 | 3.87–3.93 |
| 3. South^‡^ | 4959 | 3.93 | 0.01 | 3.91–3.96 |
| 4. West^§^ | 3994 | 3.78 | 0.01 | 3.76–3.81 |

*Note.* Higher score indicates greater gender bias in cultural tightness perceptions. All analyses controlled for the state level effect.

^*^ Connecticut, Maine, Massachusetts, New Hampshire, New Jersey, New York, Pennsylvania, Rhode Island, and Vermont.

^†^ Illinois, Indiana, Iowa, Kansas, Michigan, Minnesota, Missouri, Nebraska, North Dakota, Ohio, South Dakota, and Wisconsin.

^‡^ Alabama, Arkansas, Delaware, Florida, Georgia, Kentucky, Louisiana, Maryland, Mississippi, North Carolina, Oklahoma, South Carolina, Tennessee, Texas, Virginia, and West Virginia.

^§^ Alaska, Arizona, California, Colorado, Hawaii, Idaho, Montana, Nevada, New Mexico, Oregon, Utah, Washington, and Wyoming.

**Table S5. Gender bias in cultural tightness perceptions (at the individual level) for the nine U.S. census regional divisions.**

| Regional Division | N | Mean | Standard Error | 95% Confidence Interval |
| --- | --- | --- | --- | --- |
| 1. New England^*^ | 1828 | 3.77 | 0.02 | 3.73–3.80 |
| 2. Middle Atlantic^†^ | 947 | 3.70 | 0.03 | 3.65–3.75 |
| 3. East North Central^‡^ | 1549 | 3.97 | 0.02 | 3.93–4.01 |
| 4. West North Central^§^ | 2148 | 3.85 | 0.02 | 3.82–3.89 |
| 5. South Atlantic^¶^ | 2473 | 3.87 | 0.02 | 3.84–3.90 |
| 6. East South Central^\|\|^ | 1232 | 4.04 | 0.02 | 4.00–4.09 |
| 7. West South Central^**^ | 1254 | 3.96 | 0.02 | 3.92–4.00 |
| 8. Mountain^††^ | 2443 | 3.87 | 0.02 | 3.84–3.90 |
| 9. Pacific^‡‡^ | 1551 | 3.64 | 0.02 | 3.60–3.68 |

*Note.* Higher score indicates greater gender bias in cultural tightness perceptions. All analyses controlled for the state level effect.

^*^Connecticut, Maine, Massachusetts, New Hampshire, Rhode Island, and Vermont.

^†^ New Jersey, New York, and Pennsylvania.

^‡^ Illinois, Indiana, Michigan, Ohio, and Wisconsin.

^§^ Iowa, Kansas, Minnesota, Missouri, Nebraska, North Dakota, and South Dakota.

^¶^ Delaware, Florida, Georgia, Maryland, North Carolina, South Carolina, Virginia, and West Virginia.

^||^ Alabama, Kentucky, Mississippi, and Tennessee.

^**^ Arkansas, Louisiana, Oklahoma, and Texas.

^††^ Arizona, Colorado, Idaho, Montana, Nevada, New Mexico, Utah, and Wyoming.

^‡‡^ Alaska, California, Hawaii, Oregon, and Washington.

**Table S6. Links between gender bias in cultural tightness and gender inequality in entrepreneurship.**

| Variables | Gender inequality in number of startup firms | | Gender inequality in all firm ownership | |
| --- | --- | --- | --- | --- |
|  | Model 1 | Model 2 | Model 3 | Model 4 |
| Gender bias in cultural tightness | **0.02^***^ (0.01)** | **0.03^***^ (0.01)** | **0.02^**^ (0.01)** | **0.02^**^ (0.01)** |
| GDP per capita (log) |  | 0.08^**^ (0.03) |  | 0.11^**^ (0.04) |
| Gender imbalance in population (more men than women) |  | -0.62^***^ (0.18) |  | -0.17 (0.24) |
| State cultural tightness |  | -0.00 (0.00) |  | 0.00 (0.00) |
| Constant | 0.61^***^ (0.02) | -0.28 (0.35) | 0.30^***^ (0.02) | -0.90^*^ (0.41) |
| Year | 2014-2016 | 2014-2016 | 2002-2012 | 2002-2012 |
| *N* | 150 | 150 | 150 | 150 |

^+^*P* < 0.1; ^*^*P* < 0.05; ^**^*P* < 0.01; ^***^*P* < 0.001. Results from hierarchical linear modeling. Unstandardized regression coefficients are reported. Standard errors in parentheses.

^†^ Gender inequality in number of startup firms was obtained and computed from the Annual Survey of Entrepreneurs (ASE; 2014-2016), while gender inequality in all firm ownership was obtained and computed from the Survey of Business Owners (SBO; 2002-2012) from the U.S. Census Bureau.

**Table S7. Summary of the relationships between socio-political factors and gender-related threats and gender bias in cultural tightness versus three gender equality scores.**

| Domain | ID | Variables | Gender bias in cultural tightness | | State gender parity index | | State gender equality score | | State municipal equality index | |
| --- | --- | --- | --- | --- | --- | --- | --- | --- | --- | --- |
| Socio-political factors: religion | 1 | Percentage of adults who are highly religious | **4.46^***^ (1.22)** | √ | **-58.97^***^**  **(14.47)** | √ | **-49.68^***^ (12.61** | √ | **-92.01^***^ (23.72)** | √ |
|  | 2 | Importance of religion | **4.41^**^ (1.27)** | √ | **-57.27^***^**  **(15.15)** | √ | **-50.54^***^ (13.00)** | √ | **-96.17^***^ (24.22)** | √ |
|  | 3 | Frequency of prayer | **4.82^***^ (1.37)** | √ | **-65.11^***^**  **(16.11)** | √ | **-51.58^***^ (14.30)** | √ | **-98.37^***^ (26.65)** | √ |
|  | 4 | Worship attendance | **7.58^***^ (1.53)** | √ | **-79.15^***^**  **(19.88)** | √ | **-82.13^***^ (15.81)** | √ | **-118.34^***^ (32.98)** | √ |
|  | 5 | Belief in God | **4.50^**^ (1.42)** | √ | **-64.46^***^ (16.54)** | √ | **-45.15^**^ (15.07)** | √ | **-100.62^***^ (27.08)** | √ |
|  | 6 | Religious belief | **7.10^**^ (2.27)** | √ | **-98.65^***^**  **(26.67)** | √ | **-76.10^**^ (23.72)** | √ | **-150.36^**^ (43.88)** | √ |
|  | 7 | Non-religious^†^ | **-6.97^**^ (2.32)** | √ | **99.06^***^**  **(27.17)** | √ | **77.08^**^ (24.10)** | √ | **156.79^***^ (44.25)** | √ |
|  | 8 | Percentage of adults who are very religious | **6.48^***^ (1.33)** | √ | **-70.12^***^**  **(17.01)** | √ | **-72.26^***^ (13.50)** | √ | **-116.98^***^ (27.21)** | √ |
|  | 9 | Percentage of adults who are moderately religious | 0.60 (4.56) | × | -38.92  (55.22) | × | 68.69 (46.87) | × | 28.85 (89.75) | × |
|  | 10 | Percentage of adults who are non-religious | **-5.47^***^ (1.24)** | √ | **61.79^***^**  **(15.48)** | √ | **51.94^***^ (13.49)** | √ | **94.26^***^ (25.54)** | √ |
| Socio-political factors: political ideology | 11 | Percentage of people having conservative political ideology | **11.65^***^**  **(1.81)** | √ | **-112.55^***^**  **(25.40)** | √ | **-95.43^***^ (22.10)** | √ | **-142.98^**^ (44.34)** | √ |
|  | 12 | Percentage of Republicans in the U.S. Senate | **1.28^***^**  **(0.26)** | √ | **-14.79^***^**  **(3.18)** | √ | **-14.25^***^ (2.59)** | √ | **-18.53^**^ (5.61)** | √ |
|  | 13 | Percentage of Republicans in the House of Representatives | **1.74^***^**  **(0.33)** | √ | **-22.05^***^ (3.91)** | √ | **-18.48^***^ (3.45)** | √ | **-20.53^*^ (7.66)** | √ |
| Gender-related threats | 14 | State sexism belief i | **3.27^**^ (0.93)** | √ | 0.97 (12.58) | × | **-27.16^*^ (10.26)** | √ | -29.39 (19.87) | × |
|  | 15 | State sexism belief ii | **4.74^***^ (1.00)** | √ | **-51.68^***^ (12.84)** | √ | **-43.60^***^ (10.72)** | √ | **-73.58^**^ (21.49)** | √ |
|  | 16 | Percentage of people favoring non-discrimination LGBT protection | **-6.67^*^**  **(3.16)** | √ | **110.36^**^ (36.82)** | √ | 41.89 (34.21) | × | **233.36^***^ (55.31)** | √ |
|  | 17 | Percentage of people viewing homosexuality as acceptable | **-5.82^***^**  **(1.43)** | √ | **64.12^***^ (17.99)** | √ | **50.02^**^ (15.93)** | √ | **120.18^***^ (27.70)** | √ |
|  | 18 | Percentage of male-dominated industries | **13.03^***^ (3.64)** | √ | **-134.23^**^ (45.94)** | √ | **-110.34^**^ (40.03)** | √ | **-208.21^**^ (74.92)** | √ |
|  | 19 | Sexual violence against women | -0.06  (0.05) | × | 0.88  (0.60) | × | 0.03  (0.51) | × | -0.20  (0.93) | × |
|  | 20 | Relative domestic violence | 0.23  (0.36) | × | 4.28  (4.39) | × | 0.82  (3.80) | × | 4.31  (7.20) | × |
|  | 21 | Relative human trafficking | -0.23  (0.18) | × | 0.82  (2.19) | × | 3.42^+^  (1.83) | × | 2.24  (3.54) | × |
|  |  | Percentage of supported propositions | 17/21 = 81.0% | | 16/21 = 76.2% | | 16/21 = 76.2% | | 16/21 = 76.2% | |

*Note.* Percentage of supported propositions = the number of supported propositions / the number of propositions. √ represents that the proposition is supported, while × represents that the proposition is not supported. The statistical criteria of determining whether a proposition is supported or not is whether the effect is significant (*P* < 0.05) in the expected direction (i.e., the direction of the coefficient is as the same as the direction of the proposition).

^†^ Regarding the key religious affiliation (e.g., the percentage of Buddhists, Catholics, Evangelical Protestants, Hindus, Black Protestants, Jews, Mainline Protestants, and Mormons), we did not expect the specific type of religious affiliations to be related to gender bias in cultural tightness, thus we did not include them in the table.

**Table S8. Summary of the relationships between gender bias in cultural tightness versus three gender equality scores (included separately) and gender inequality in leadership and innovation.**

| Domain | ID | Variables | Gender bias in cultural tightness | | State gender parity index | | State gender equality score | | State municipal equality index | |
| --- | --- | --- | --- | --- | --- | --- | --- | --- | --- | --- |
| Business leadership | 1 | Gender inequality in boards of publicly traded companies | **0.03^*^ (0.01)** | √ | -0.00 (0.00) | × | **-0.00^*^ (0.00)** | √ | -0.00 (0.00) | × |
|  | 2 | Gender inequality in CEOs of publicly traded companies | **0.04^*^ (0.02)** | √ | 0.00 (0.00) | × | -0.00 (0.00) | × | 0.00 (0.00) | × |
|  | 3 | Gender inequality in management occupations: total | **0.03^**^ (0.01)** | √ | -0.00 (0.00) | × | **-0.00^***^ (0.00)** | √ | 0.00 (0.00) | × |
|  | 4 | Gender inequality in management occupations: top executives | **0.03^***^ (0.01)** | √ | -0.00 (0.00) | × | **-0.00^***^ (0.00)** | √ | 0.00^*^ (0.00) | × |
|  | 5 | Gender inequality in business leadership (aggregated) | **0.31^***^ (0.07)** | √ | **-0.02^*^ (0.01)** | √ | **-0.03^***^ (0.01)** | √ | 0.00 (0.00) | × |
| Political leadership | 6 | Gender inequality in U.S. Senators | **0.07^*^ (0.03)** | √ | -0.00 (0.00) | × | -0.00 (0.00) | × | -0.00 (0.00) | × |
|  | 7 | Gender inequality in U.S. Representatives | 0.02 (0.02) | × | -0.00 (0.00) | × | -0.00 (0.00) | × | 0.00 (0.00) | × |
|  | 8 | Gender inequality in State Senators | **0.05^**^ (0.01)** | √ | **-0.00^**^ (0.00)** | √ | -0.00 (0.00) | × | 0.00 (0.00) | × |
|  | 9 | Gender inequality in State Representatives | **0.04^**^ (0.01)** | √ | -0.00 (0.00) | × | 0.00 (0.00) | × | 0.00 (0.00) | × |
|  | 10 | Gender inequality in State Governors | **0.75^*^ (0.37)** | √ | **-0.07^*^ (0.03)** | √ | 0.00 (0.04) | × | 0.03 (0.02) | × |
|  | 11 | Gender inequality in political leadership (aggregated) | **0.18^***^ (0.05)** | √ | **-0.01^**^ (0.00)** | √ | -0.00 (0.01) | × | 0.00 (0.00) | × |
| Innovation | 12 | Gender inequality in patent inventors: utility | **0.01^***^ (0.00)** | √ | 0.00 (0.00) | × | **-0.00^*^ (0.00)** | √ | -0.00 (0.00) | × |
|  | 13 | Gender inequality in patent inventors: design | -0.01 (0.01) | × | 0.00 (0.00) | × | -0.00 (0.00) | × | 0.00^+^ (0.00) | × |
|  | 14 | Gender inequality in patent inventors: plant | 0.07 (0.12) | × | 0.01 (0.01) | × | -0.00 (0.01) | × | 0.01^+^ (0.01) | × |
|  | 15 | Gender inequality in patent inventors (aggregate of design, plant, and utility patents) | **0.01^**^ (0.00)** | √ | 0.00 (0.00) | × | **-0.00^*^ (0.00)** | √ | -0.00 (0.00) | × |
|  | 16 | Gender inequality in STEM occupations | **0.01^**^ (0.00)** | √ | 0.00^+^ (0.00) | × | **-0.00^***^ (0.00)** | √ | 0.00 (0.00) | × |
|  | 17 | Gender inequality in attainment of doctoral degrees^†^ | **0.01^***^ (0.00)** | √ | 0.00 (0.00) | × | **-0.00^*^ (0.00)** | √ | 0.00 (0.00) | × |
|  | 18 | Gender inequality in number of startup firms | **0.03^***^ (0.01)** | √ | -0.00 (0.00) | × | -0.00^+^ (0.00) | × | -0.00 (0.00) | × |
|  | 19 | Gender inequality in all firm ownership | **0.02^**^ (0.01)** | √ | -0.00^+^ (0.00) | × | -0.00 (0.00) | × | -0.00 (0.00) | × |
|  |  | Percentage of supported propositions | 16/19 = 84.2% | | 4/19 = 21.1% | | 8/19 = 42.1% | | 0/19 = 0.0% | |

*Note.* Percentage of supported propositions = the number of supported propositions / the number of propositions. √ represents that the proposition is supported, while × represents that the proposition is not supported. The statistical criteria of determining whether a proposition is supported or not is whether the effect is significant (*P* < 0.05) in the expected direction (i.e., the direction of the coefficient is as the same as the direction of the proposition).

^†^ Regarding gender inequality in attainment of bachelor’s degrees, gender inequality in attainment of master’s degrees, and gender inequality in attainment of professional degrees, we did not expect them to be related to gender bias in cultural tightness, thus we did not include them in the table.

**Table S9.** **Summary of the relationships between gender bias in cultural tightness versus three gender equality scores (included together) and gender inequality in leadership and innovation.**

| Domain | ID | Variables | Gender bias in cultural tightness *included* *alone* | | Gender bias in cultural tightness and three gender equality scores *included together* | | | | | | | | | | |
| --- | --- | --- | --- | --- | --- | --- | --- | --- | --- | --- | --- | --- | --- | --- | --- |
|  |  |  | Gender bias in cultural tightness | | Gender bias in cultural tightness | | State gender parity index^‡^ | | Effect size difference between gender bias in cultural tightness and state gender parity index^§^ | State gender equality score | | Effect size difference between gender bias in cultural tightness and state gender equality score | State municipal equality index | | Effect size difference between gender bias in cultural tightness and state municipal equality index |
| Business leadership | 1 | Gender inequality in boards of publicly traded companies | **0.03^*^ (0.01)** | √ | 0.03^+^ (0.01) | × | -0.00 (0.00) | × | *P* = 0.091 | -0.00 (0.00) | × | *P* = 0.100 | 0.00 (0.00) | × | *P* = 0.075 |
|  | 2 | Gender inequality in CEOs of publicly traded companies | **0.04^*^ (0.02)** | √ | **0.05^*^ (0.02)** | √ | 0.00^*^ (0.00) | × | *P* = 0.008 | -0.00 (0.00) | × | *P* = 0.021 | 0.00^*a^ (0.00) | × | *P* = 0.010 |
|  | 3 | Gender inequality in management occupations: total | **0.03^**^ (0.01)** | √ | **0.03^*^ (0.01)** | √ | 0.00 (0.00) | × | *P* = 0.031 | **-0.00^*^ (0.00)** | √ | *P* = 0.068 | 0.00^*a^ (0.00) | × | *P* = 0.023 |
|  | 4 | Gender inequality in management occupations: top executives | **0.03^***^ (0.01)** | √ | **0.02^***^ (0.00)** | √ | 0.00 (0.00) | × | *P* < 0.001 | **-0.00^***^ (0.00)** | √ | *P* = 0.006 | 0.00^***a^ (0.00) | × | *P* < 0.001 |
|  | 5 | Gender inequality in business leadership (aggregated) | **0.31^***^ (0.07)** | √ | **0.24^***^ (0.07)** | √ | -0.00 (0.01) | × | *P* = 0.001 | **-0.02^**^ (0.01)** | √ | *P* = 0.003 | 0.01^+^ (0.00) | × | *P* < 0.001 |
| Political leadership | 6 | Gender inequality in U.S. Senators | **0.07^*^ (0.03)** | √ | **0.08^*^ (0.03)** | √ | -0.00 (0.00) | × | *P* = 0.023 | 0.00 (0.00) | × | *P* = 0.018 | -0.00 (0.00) | × | *P* = 0.021 |
|  | 7 | Gender inequality in U.S. Representatives | 0.02 (0.02) | × | 0.01 (0.03) | × | -0.00 (0.00) | × | *-* | -0.00 (0.00) | × | *-* | 0.00 (0.00) | × | *-* |
|  | 8 | Gender inequality in State Senators | **0.05^**^ (0.01)** | √ | **0.05^***^ (0.01)** | √ | **-0.00^**^ (0.00)** | √ | *P* = 0.002 | 0.00 (0.00) | × | *P* < 0.001 | 0.00 (0.00) | × | *P* < 0.001 |
|  | 9 | Gender inequality in State Representatives | **0.04^**^ (0.01)** | √ | **0.05^***^ (0.01)** | √ | -0.00 (0.00) | × | *P* < 0.001 | 0.00^*a^ (0.00) | × | *P* < 0.001 | 0.00 (0.00) | × | *P* < 0.001 |
|  | 10 | Gender inequality in State Governors | **0.75^*^ (0.37)** | √ | **0.97^*^ (0.39)** | √ | **-0.07^*^ (0.03)** | √ | *P* = 0.022 | 0.06^+^ (0.04) | × | *P* = 0.011 | 0.03 (0.02) | × | *P* = 0.011 |
|  | 11 | Gender inequality in political leadership (aggregated) | **0.18^***^ (0.05)** | √ | **0.21^***^ (0.06)** | √ | **-0.01^**^ (0.00)** | √ | *P* < 0.001 | 0.01^+^ (0.01) | × | *P* < 0.001 | 0.00 (0.00) | × | *P* < 0.001 |
| Innovation | 12 | Gender inequality in patent inventors: utility | **0.01^***^ (0.00)** | √ | **0.01^**^ (0.00)** | √ | 0.00 (0.00) | × | *P* = 0.002 | -0.00 (0.00) | × | *P* = 0.006 | -0.00 (0.00) | × | *P* = 0.003 |
|  | 13 | Gender inequality in patent inventors: design | -0.01 (0.01) | × | -0.01 (0.02) | × | 0.00 (0.00) | × | *-* | -0.00 (0.00) | × | *-* | 0.00^*a^ (0.00) | × | *-* |
|  | 14 | Gender inequality in patent inventors: plant | 0.07 (0.12) | × | 0.10 (0.12) | × | 0.01 (0.01) | × | *-* | -0.01 (0.01) | × | *-* | 0.01^*a^ (0.01) | × | *-* |
|  | 15 | Gender inequality in patent inventors (aggregate of design, plant, and utility patents) | **0.01^**^ (0.00)** | √ | **0.01^*^ (0.00)** | √ | 0.00 (0.00) | × | *P* = 0.019 | -0.00 (0.00) | × | *P* = 0.040 | 0.00 (0.00) | × | *P* = 0.023 |
|  | 16 | Gender inequality in STEM occupations | **0.01^**^ (0.00)** | √ | **0.01^*^ (0.00)** | √ | 0.00^***a^ (0.00) | × | *P* = 0.009 | **-0.00^***^ (0.00)** | √ | *P* = 0.071 | 0.00^+^ (0.00) | × | *P* = 0.018 |
|  | 17 | Gender inequality in attainment of doctoral degrees^†^ | **0.01^***^ (0.00)** | √ | **0.01^**^ (0.00)** | √ | 0.00 (0.00) | × | *P* = 0.005 | -0.00 (0.00) | × | *P* = 0.015 | 0.00 (0.00) | × | *P* = 0.006 |
|  | 18 | Gender inequality in number of startup firms | **0.03^***^ (0.01)** | √ | **0.03^***^ (0.01)** | √ | -0.00 (0.00) | × | *P* < 0.001 | 0.00 (0.00) | × | *P* = 0.001 | 0.00 (0.00) | × | *P* < 0.001 |
|  | 19 | Gender inequality in all firm ownership | **0.02^**^ (0.01)** | √ | **0.02^*^ (0.01)** | √ | -0.00 (0.00) | × | *P* = 0.046 | 0.00 (0.00) | × | *P* = 0.040 | -0.00 (0.00) | × | *P* = 0.037 |
|  |  | Percentage of supported propositions | 16/19 = 84.2% | | 15/19 = 78.9% | | 3/19 = 15.8% | | 15/16 = 93.8% | 4/19 = 21.1% | | 13/16 = 81.3% | 0/19 = 0.0% | | 15/16 = 93.8% |

*Note.* Percentage of supported propositions = the number of supported propositions / the number of propositions. √ represents that the proposition is supported, while × represents that the proposition is not supported. For Columns 7, 9 and 11, percentage of supported propositions = the number of effect sizes that have significant differences / the number of supported propositions. The statistical criteria of determining whether a proposition is supported or not is whether the effect is significant (*P* < 0.05) in the expected direction (i.e., the direction of the coefficient is as the same as the direction of the proposition).

^†^ Regarding gender inequality in attainment of bachelor’s degrees, gender inequality in attainment of master’s degrees, and gender inequality in attainment of professional degrees, we did not expect them to be related to gender bias in cultural tightness, thus we did not include them in the table.

^‡^ For each of three gender equality scores, when its coefficient was significantly negative (rather than significantly positive), the proposition was supported.

^§^ To test the effect size differences between gender bias in cultural tightness and the three gender equality scores, we conducted a series of regressions by including gender bias in cultural tightness and the three gender equality scores simultaneously to predict our outcomes. Then, we tested that (1) whether gender bias in cultural tightness had incremental effects on these outcomes beyond the effects of the three gender equality scores (i.e., the coefficient of gender bias in cultural tightness was significant in the expected direction), and (2) whether the coefficient of gender bias in cultural tightness was significantly larger than the coefficients of the three gender equality scores, respectively.

**Table S10. Descriptive summary of the sample’s gender, age, and education level with those of the general population within the state.**

| State | Gender distribution of the sample | Gender distribution of the general population | The difference of gender | Average age of the sample | Average age of the general population | The difference of age | Average education level of the sample | Average education level of the general population | The difference of education level |
| --- | --- | --- | --- | --- | --- | --- | --- | --- | --- |
| Alabama | 0.41 | 0.48 | -0.07 | 36.87 | 39.50 | -2.63 | 0.59 | 0.20 | 0.39 |
| Alaska | 0.52 | 0.52 | -0.01 | 33.30 | 35.30 | -2.00 | 0.68 | 0.21 | 0.47 |
| Arizona | 0.46 | 0.50 | -0.04 | 36.40 | 38.50 | -2.10 | 0.56 | 0.23 | 0.33 |
| Arkansas | 0.37 | 0.49 | -0.12 | 36.44 | 38.60 | -2.16 | 0.59 | 0.22 | 0.37 |
| California | 0.51 | 0.50 | 0.01 | 35.77 | 37.30 | -1.53 | 0.74 | 0.27 | 0.47 |
| Colorado | 0.40 | 0.50 | -0.11 | 35.84 | 37.30 | -1.46 | 0.72 | 0.33 | 0.40 |
| Connecticut | 0.36 | 0.49 | -0.13 | 33.41 | 41.20 | -7.79 | 0.75 | 0.32 | 0.43 |
| Delaware | 0.45 | 0.48 | -0.03 | 36.32 | 41.40 | -5.08 | 0.70 | 0.25 | 0.45 |
| Florida | 0.45 | 0.49 | -0.04 | 38.95 | 42.70 | -3.75 | 0.60 | 0.28 | 0.32 |
| Georgia | 0.37 | 0.49 | -0.12 | 38.02 | 37.30 | 0.72 | 0.61 | 0.24 | 0.37 |
| Hawaii | 0.44 | 0.50 | -0.06 | 34.38 | 40.00 | -5.62 | 0.65 | 0.27 | 0.38 |
| Idaho | 0.44 | 0.50 | -0.06 | 33.71 | 37.20 | -3.49 | 0.59 | 0.23 | 0.37 |
| Illinois | 0.49 | 0.49 | 0.00 | 37.61 | 38.80 | -1.19 | 0.65 | 0.29 | 0.36 |
| Indiana | 0.42 | 0.49 | -0.07 | 35.30 | 38.00 | -2.70 | 0.60 | 0.23 | 0.37 |
| Iowa | 0.36 | 0.50 | -0.14 | 36.90 | 38.60 | -1.70 | 0.62 | 0.22 | 0.40 |
| Kansas | 0.38 | 0.50 | -0.12 | 36.71 | 37.30 | -0.59 | 0.57 | 0.23 | 0.34 |
| Kentucky | 0.43 | 0.49 | -0.06 | 38.18 | 39.20 | -1.02 | 0.59 | 0.20 | 0.39 |
| Louisiana | 0.40 | 0.49 | -0.09 | 36.00 | 37.80 | -1.80 | 0.65 | 0.20 | 0.45 |
| Maine | 0.38 | 0.49 | -0.10 | 34.44 | 45.00 | -10.56 | 0.57 | 0.26 | 0.31 |
| Maryland | 0.46 | 0.48 | -0.03 | 36.26 | 39.20 | -2.94 | 0.67 | 0.36 | 0.32 |
| Massachusetts | 0.42 | 0.49 | -0.07 | 35.37 | 39.70 | -4.33 | 0.66 | 0.38 | 0.28 |
| Michigan | 0.37 | 0.49 | -0.13 | 36.96 | 40.10 | -3.14 | 0.63 | 0.25 | 0.37 |
| Minnesota | 0.43 | 0.50 | -0.06 | 35.25 | 38.50 | -3.25 | 0.67 | 0.29 | 0.38 |
| Mississippi | 0.44 | 0.48 | -0.05 | 35.25 | 38.30 | -3.05 | 0.63 | 0.18 | 0.45 |
| Missouri | 0.37 | 0.49 | -0.12 | 37.44 | 39.10 | -1.66 | 0.55 | 0.25 | 0.30 |
| Montana | 0.46 | 0.50 | -0.04 | 35.29 | 40.20 | -4.91 | 0.67 | 0.25 | 0.42 |
| Nebraska | 0.44 | 0.50 | -0.06 | 35.14 | 36.90 | -1.76 | 0.63 | 0.24 | 0.39 |
| Nevada | 0.43 | 0.50 | -0.07 | 35.70 | 38.50 | -2.80 | 0.60 | 0.21 | 0.38 |
| New Hampshire | 0.43 | 0.50 | -0.07 | 34.04 | 43.10 | -9.06 | 0.59 | 0.30 | 0.29 |
| New Jersey | 0.48 | 0.49 | -0.01 | 36.00 | 40.20 | -4.20 | 0.59 | 0.34 | 0.25 |
| New Mexico | 0.47 | 0.49 | -0.03 | 35.11 | 38.60 | -3.49 | 0.64 | 0.22 | 0.42 |
| New York | 0.46 | 0.49 | -0.02 | 36.14 | 39.40 | -3.26 | 0.65 | 0.32 | 0.33 |
| North Carolina | 0.37 | 0.49 | -0.11 | 38.49 | 39.20 | -0.71 | 0.64 | 0.25 | 0.39 |
| North Dakota | 0.46 | 0.51 | -0.05 | 34.04 | 35.40 | -1.36 | 0.72 | 0.22 | 0.50 |
| Ohio | 0.42 | 0.49 | -0.07 | 36.58 | 39.60 | -3.02 | 0.59 | 0.24 | 0.36 |
| Oklahoma | 0.39 | 0.50 | -0.10 | 35.77 | 37.10 | -1.33 | 0.45 | 0.21 | 0.24 |
| Oregon | 0.40 | 0.50 | -0.10 | 36.43 | 39.90 | -3.47 | 0.66 | 0.29 | 0.37 |
| Pennsylvania | 0.37 | 0.49 | -0.12 | 39.02 | 40.90 | -1.88 | 0.49 | 0.28 | 0.22 |
| Rhode Island | 0.45 | 0.49 | -0.04 | 33.95 | 40.30 | -6.35 | 0.45 | 0.30 | 0.14 |
| South Carolina | 0.41 | 0.48 | -0.08 | 36.27 | 40.10 | -3.83 | 0.65 | 0.26 | 0.40 |
| South Dakota | 0.46 | 0.50 | -0.05 | 35.05 | 37.60 | -2.55 | 0.60 | 0.21 | 0.38 |
| Tennessee | 0.38 | 0.49 | -0.11 | 36.92 | 39.10 | -2.18 | 0.43 | 0.23 | 0.20 |
| Texas | 0.48 | 0.50 | -0.01 | 36.40 | 35.20 | 1.20 | 0.74 | 0.23 | 0.51 |
| Utah | 0.50 | 0.50 | 0.00 | 33.07 | 31.50 | 1.57 | 0.62 | 0.24 | 0.38 |
| Vermont | 0.43 | 0.49 | -0.07 | 36.90 | 43.00 | -6.10 | 0.46 | 0.37 | 0.09 |
| Virginia | 0.43 | 0.49 | -0.07 | 36.14 | 38.70 | -2.56 | 0.60 | 0.34 | 0.27 |
| Washington | 0.42 | 0.50 | -0.08 | 36.35 | 37.90 | -1.55 | 0.67 | 0.27 | 0.40 |
| West Virginia | 0.42 | 0.50 | -0.08 | 34.91 | 43.00 | -8.09 | 0.49 | 0.17 | 0.31 |
| Wisconsin | 0.39 | 0.50 | -0.10 | 37.57 | 40.00 | -2.43 | 0.70 | 0.25 | 0.46 |
| Wyoming | 0.48 | 0.51 | -0.03 | 34.14 | 38.70 | -4.56 | 0.63 | 0.21 | 0.42 |

*Note.* gender distribution = the number of men/[the number of men + the number of women]; average age, in years; average education level = the number of equal to or higher than a bachelor’s degree/the total number.

**Table S11. Relationships between differences between sample and population demographics and gender bias in cultural tightness.**

| Variables | Model 1 |
| --- | --- |
| The difference in gender^†^ | -3.15 (3.74) |
| The difference in age | 0.06 (0.06) |
| The difference in education level | 1.83 (1.81) |
| Constant | 2.31^**^ (0.82) |
| *N* | 50 |

^+^*P* < 0.1; ^*^*P* < 0.05; ^**^*P* < 0.01; ^***^*P* < 0.001. Results from ordinary least squares regressions. Unstandardized regression coefficients are reported. Standard errors in parentheses.

^†^ Differences in age, gender, and education level were computed by subtracting our sample’s average age, gender, and education level at the state level from those of the general population within the state. The average age, gender, and education level of each state from the U.S. Census Bureau (2020).

**Table S12. Links between religion (Pew) and gender bias in cultural tightness (Wave 1).**

| Variables | Model 1 | Model 2 | Model 3 | Model 4 | Model 5 | Model 6 | Model 7 | Model 8 | Model 9 | Model 10 | Model 11 | Model 12 |
| --- | --- | --- | --- | --- | --- | --- | --- | --- | --- | --- | --- | --- |
| Percentage of adults who are highly religious^†^ | **4.93^***^ (1.14)** | **3.97^**^ (1.29)** |  |  |  |  |  |  |  |  |  |  |
| Importance of religion |  |  | **4.76^***^ (1.15)** | **3.77^**^ (1.35)** |  |  |  |  |  |  |  |  |
| Frequency of prayer |  |  |  |  | **5.51^***^ (1.31)** | **4.38^**^ (1.44)** |  |  |  |  |  |  |
| Worship attendance |  |  |  |  |  |  | **8.18^***^ (1.49)** | **7.09^***^ (1.63)** |  |  |  |  |
| Belief in God |  |  |  |  |  |  |  |  | **5.15^***^ (1.32)** | **3.89^*^ (1.50)** |  |  |
| Religious belief |  |  |  |  |  |  |  |  |  |  | **7.30^**^ (2.26)** | **6.14^*^ (2.39)** |
| GDP per capita (log): 1977-2020 |  | -1.34^+^ (0.78) |  | -1.33 (0.81) |  | -1.44^+^ (0.77) |  | -1.21^+^ (0.71) |  | -1.42^+^ (0.81) |  | -1.96^*^ (0.74) |
| Gender imbalance in population: 1970-2020 (more men than women) |  | 1.24 (3.82) |  | 1.51 (3.90) |  | 0.85 (3.81) |  | 1.04 (3.51) |  | 0.48 (3.89) |  | 3.12 (4.11) |
| Constant | 0.30 (0.64) | 14.94^+^ (8.60) | 0.49 (0.62) | 15.07^+^ (8.88) | 0.02 (0.72) | 15.78^+^ (8.49) | 0.08 (0.55) | 13.22^+^ (7.71) | -0.27 (0.85) | 15.44^+^ (9.06) | -2.56 (1.73) | 19.03^*^ (8.43) |
| *N* | 50 | 50 | 50 | 50 | 50 | 50 | 50 | 50 | 50 | 50 | 50 | 50 |
| *R*^2^ | 0.28 | 0.32 | 0.26 | 0.30 | 0.27 | 0.32 | 0.38 | 0.42 | 0.24 | 0.29 | 0.18 | 0.29 |
| *F* | 18.70 | 7.32 | 17.03 | 6.66 | 17.73 | 7.26 | 29.99 | 11.17 | 15.25 | 6.23 | 10.44 | 6.17 |

^+^*P* < 0.1; ^*^*P* < 0.05; ^**^*P* < 0.01; ^***^*P* < 0.001. Results from ordinary least squares regressions. Unstandardized regression coefficients are reported. Standard errors in parentheses.

^†^ Religious data in this table were obtained and computed from the Pew Research Center (2014).

**Table S13. Links between religion (Pew, breakdown of key religious affiliation) and gender bias in cultural tightness (Wave 1).**

| Variables | Model 1 | Model 2 | Model 3 | Model 4 | Model 5 | Model 6 | Model 7 | Model 8 | Model 9 | Model 10 | Model 11 | Model 12 | Model 13 | Model 14 | Model 15 | Model 16 | Model 17 | Model 18 |
| --- | --- | --- | --- | --- | --- | --- | --- | --- | --- | --- | --- | --- | --- | --- | --- | --- | --- | --- |
| Non-religious | **-7.30^**^ (2.31)** | **-6.17^*^ (2.43)** |  |  |  |  |  |  |  |  |  |  |  |  |  |  |  |  |
| Buddhists^†‡§^ |  |  | **-20.84^+^ (11.83)** | **-13.22 (11.91)** |  |  |  |  |  |  |  |  |  |  |  |  |  |  |
| Catholics |  |  |  |  | **-5.25^**^ (1.45)** | **-4.13^*^ (1.72)** |  |  |  |  |  |  |  |  |  |  |  |  |
| Evangelical Protestants |  |  |  |  |  |  | **3.46^**^ (1.22)** | **1.95 (1.40)** |  |  |  |  |  |  |  |  |  |  |
| Hindus |  |  |  |  |  |  |  |  | **-64.85^**^ (19.67)** | **-58.24^*^ (24.06)** |  |  |  |  |  |  |  |  |
| Black Protestants |  |  |  |  |  |  |  |  |  |  | **4.34^+^ (2.42)** | **4.02 (2.57)** |  |  |  |  |  |  |
| Jews |  |  |  |  |  |  |  |  |  |  |  |  | **-27.38^**^ (8.98)** | **-28.40^*^ (11.37)** |  |  |  |  |
| Mainline Protestants |  |  |  |  |  |  |  |  |  |  |  |  |  |  | **0.29 (2.47)** | **-0.50 (2.29)** |  |  |
| Mormons |  |  |  |  |  |  |  |  |  |  |  |  |  |  |  |  | **3.84^*^ (1.69)** | **3.96^*^ (1.67)** |
| GDP per capita (log): 1977-2020 |  | -1.99^*^ (0.74) |  | -2.23^**^ (0.78) |  | -1.21 (0.88) |  | -1.74^+^ (0.89) |  | -1.12 (0.90) |  | -2.35^**^ (0.76) |  | -1.05 (0.90) |  | -2.39^**^ (0.78) |  | -2.03^**^ (0.75) |
| Gender imbalance in population: 1970-2020 (more men than women) |  | 3.13 (4.12) |  | 0.94 (4.25) |  | -3.17 (4.09) |  | -0.57 (4.07) |  | -5.35 (4.43) |  | 3.01 (4.57) |  | -7.16 (4.76) |  | -0.32 (4.15) |  | -3.90 (4.20) |
| Constant | 4.69^***^ (0.55) | 25.44^**^ (7.75) | 3.15^***^ (0.16) | 26.54^**^ (8.21) | 4.00^***^ (0.30) | 16.41^+^ (9.11) | 2.10^***^ (0.34) | 20.74^*^ (9.57) | 3.23^***^ (0.15) | 14.83 (9.45) | 2.76^***^ (0.19) | 27.60^**^ (8.00) | 3.33^***^ (0.17) | 14.18 (9.48) | 2.95^***^ (0.43) | 28.24^**^ (8.29) | 2.89^***^ (0.14) | 24.05^**^ (7.93) |
| *N* | 50 | 50 | 50 | 50 | 50 | 50 | 50 | 50 | 50 | 50 | 50 | 50 | 50 | 50 | 50 | 50 | 50 | 50 |
| *R*^2^ | 0.17 | 0.28 | 0.06 | 0.21 | 0.21 | 0.28 | 0.14 | 0.22 | 0.18 | 0.28 | 0.06 | 0.23 | 0.16 | 0.28 | 0.00 | 0.19 | 0.10 | 0.27 |
| *F* | 10.03 | 6.11 | 3.10 | 3.97 | 13.03 | 5.84 | 8.09 | 4.27 | 10.87 | 5.86 | 3.22 | 4.47 | 9.29 | 6.02 | 0.01 | 3.49 | 5.12 | 5.76 |

^+^*P* < 0.1; ^*^*P* < 0.05; ^**^*P* < 0.01; ^***^*P* < 0.001. Results from ordinary least squares regressions. Unstandardized regression coefficients are reported. Standard errors in parentheses.

^†^ Religious data in this table were obtained and computed from the Pew Research Center (2014).

^‡^ Some other minor religions (e.g., Jehovah’s Witness) were considered but not significant.

^§^ To make the variables continuous, the values that were less than 1% were replaced by zero.

**Table S14. Links between religion (Gallup) and gender bias in cultural tightness (Wave 1).**

| Variables | Model 1 | Model 2 | Model 3 | Model 4 | Model 5 | Model 6 |
| --- | --- | --- | --- | --- | --- | --- |
| Percentage of adults who are very religious^†^ | **6.53^***^ (1.24)** | **5.66^***^ (1.45)** |  |  |  |  |
| Percentage of adults who are moderately religious |  |  | **-1.36 (4.71)** | **-0.54 (4.67)** |  |  |
| Percentage of adults who are non-religious |  |  |  |  | **-5.57^***^ (1.19)** | **-4.69^**^ (1.34)** |
| GDP per capita (log): 1977-2020 |  | -0.93 (0.77) |  | -2.36^**^ (0.79) |  | -1.31^+^ (0.76) |
| Gender imbalance in population: 1970-2020 (more men than women) |  | 0.65 (3.61) |  | -0.50 (4.45) |  | 1.96 (3.75) |
| Constant | 0.45 (0.50) | 10.60 (8.39) | 3.39^*^ (1.35) | 27.96^**^ (8.21) | 4.82^***^ (0.41) | 18.33^*^ (7.80) |
| *N* | 50 | 50 | 50 | 50 | 50 | 50 |
| *R*^2^ | 0.37 | 0.39 | 0.00 | 0.18 | 0.31 | 0.36 |
| *F* | 27.94 | 9.70 | 0.08 | 3.47 | 21.90 | 8.46 |

^+^*P* < 0.1; ^*^*P* < 0.05; ^**^*P* < 0.01; ^***^*P* < 0.001. Results from ordinary least squares regressions. Unstandardized regression coefficients are reported. Standard errors in parentheses.

^†^ Religious data in this table were obtained and computed from the Gallup Daily Tracking (2016).

**Table S15. Links between political ideology and gender bias in cultural tightness (Wave 1).**

| Variables | Model 1 | Model 2 | Model 3 | Model 4 | Model 5 | Model 6 |
| --- | --- | --- | --- | --- | --- | --- |
| Percentage of people having conservative political ideology^†^ | **10.06^***^**  **(1.63)** | **10.28^***^**  **(2.05)** |  |  |  |  |
| Percentage of Republicans in the U.S. Senate |  |  | **1.21^***^**  **(0.26)** | **1.09^***^**  **(0.28)** |  |  |
| Percentage of Republicans in the House of Representatives |  |  |  |  | **1.67^***^**  **(0.32)** | **1.69^***^**  **(0.35)** |
| GDP per capita (log): 1977-2020 |  | 0.05  (0.79) |  | -1.16  (0.75) |  | -0.81  (0.71) |
| Gender imbalance in population: 1970-2020 (more men than women) |  | -5.22  (3.48) |  | -4.14  (3.74) |  | -7.10^+^  (3.66) |
| Constant | -0.76  (0.62) | -1.56  (8.85) | 2.36^***^  (0.18) | 14.45^+^  (7.95) | 2.13^***^  (0.20) | 10.34  (7.61) |
| *N* | 50 | 50 | 50 | 50 | 50 | 50 |
| *R*^2^ | 0.44 | 0.47 | 0.31 | 0.38 | 0.36 | 0.46 |
| *F* | 38.21 | 13.75 | 21.38 | 9.56 | 27.29 | 13.07 |

^+^*P* < 0.1; ^*^*P* < 0.05; ^**^*P* < 0.01; ^***^*P* < 0.001. Results from ordinary least squares regressions. Unstandardized regression coefficients are reported. Standard errors in parentheses.

^†^ Percentage of people having conservative political ideology was obtained and computed from the Pew Research Center (2014), while percentages of republicans in the U.S. Senate and in the House of Representatives were obtained and computed from the Biographical Directory of the United States Congress (2019-2021).

**Table S16. Links between gender-related threats and gender bias in cultural tightness (Wave 1).**

| Variables | Model 1 | Model 2 | Model 3 | Model 4 | Model 5 | Model 6 | Model 7 | Model 8 | Model 9 | Model 10 | Model 11 | Model 12 | Model 13 | Model 14 | Model 15 | Model 16 |
| --- | --- | --- | --- | --- | --- | --- | --- | --- | --- | --- | --- | --- | --- | --- | --- | --- |
| State sexism belief i^†^ | **3.17^**^ (1.02)** | **2.60^*^ (1.00)** |  |  |  |  |  |  |  |  |  |  |  |  |  |  |
| State sexism belief ii |  |  | **4.54^***^ (0.79)** | **4.78^***^ (1.04)** |  |  |  |  |  |  |  |  |  |  |  |  |
| Percentage of people favoring non-discrimination LGBT protection |  |  |  |  | **-7.01^*^**  **(3.09)** | **-5.42**  **(3.29)** |  |  |  |  |  |  |  |  |  |  |
| Percentage of people viewing homosexuality as acceptable |  |  |  |  |  |  | **-6.15^***^**  **(1.24)** | **-5.37^***^**  **(1.51)** |  |  |  |  |  |  |  |  |
| Percentage of male-dominated industries: 2001-2018 |  |  |  |  |  |  |  |  | **13.01^***^ (3.26)** | **12.83^**^ (3.75)** |  |  |  |  |  |  |
| Sexual violence against women |  |  |  |  |  |  |  |  |  |  | **-0.05**  **(0.04)** | **-0.04**  **(0.05)** |  |  |  |  |
| Relative domestic violence |  |  |  |  |  |  |  |  |  |  |  |  | **0.33**  **(0.40)** | **0.22**  **(0.37)** |  |  |
| Relative human trafficking |  |  |  |  |  |  |  |  |  |  |  |  |  |  | **-0.26**  **(0.19)** | **-0.17**  **(0.18)** |
| GDP per capita (log): 1977-2020 |  | -1.95^*^ (0.79) |  | 0.19 (0.88) |  | -1.84^*^  (0.82) |  | -0.69  (0.84) |  | -0.96 (0.81) |  | -1.84^+^  (1.07) |  | -2.43^**^  (0.78) |  | -2.23^**^  (0.79) |
| Gender imbalance in population: 1970-2020 (more men than women) |  | -1.25 (4.47) |  | -3.75 (4.25) |  | -3.01  (4.35) |  | -1.98  (3.71) |  | -6.57 (4.14) |  | 2.49  (5.91) |  | -0.11  (4.14) |  | -0.82  (4.15) |
| Constant | -3.47 (2.09) | 18.09^+^ (9.06) | -10.02^***^ (2.28) | -12.84 (11.39) | 7.93^***^  (2.18) | 26.02^**^  (8.07) | 6.72^***^  (0.76) | 13.48  (8.34) | 0.15 (0.72) | 10.10 (9.01) | 4.35^**^  (1.21) | 23.36^*^  (11.27) | 2.56^***^  (0.57) | 28.24^**^  (8.27) | 3.52^***^  (0.40) | 26.71^**^  (8.24) |
| *N* | 49 | 49 | 48 | 48 | 50 | 50 | 50 | 50 | 50 | 50 | 41 | 41 | 49 | 49 | 50 | 50 |
| *R*^2^ | 0.17 | 0.27 | 0.42 | 0.43 | 0.10 | 0.23 | 0.34 | 0.36 | 0.25 | 0.35 | 0.03 | 0.10 | 0.01 | 0.20 | 0.04 | 0.20 |
| *F* | 9.72 | 5.63 | 32.84 | 10.92 | 5.15 | 4.58 | 24.81 | 8.62 | 15.91 | 8.24 | 1.22 | 1.43 | 0.69 | 3.83 | 1.90 | 3.83 |

^+^*P* < 0.1; ^*^*P* < 0.05; ^**^*P* < 0.01; ^***^*P* < 0.001. Results from ordinary least squares regressions. Unstandardized regression coefficients are reported. Standard errors in parentheses.

^†^ State sexism belief i was obtained and computed from the World Value Survey (2017), whereas state sexism belief ii was obtained and computed from the DDB Needham Life Style Survey (1975-1998). Percentage of people favoring non-discrimination LGBT protection was obtained and computed from the Research on LGBT in PRRI American Values Atlas (2019). Percentage of people viewing homosexuality as acceptable was obtained and computed from the Pew Research Center (2014). Percentage of male-dominated industries was obtained and computed from the NAICS Industry data from the Bureau of Economic Analysis (2001-2018). Sexual violence against women and relative domestic violence were obtained and computed from the National Intimate Partner and Sexual Violence Survey (2010). Relative human trafficking was obtained and computed from the National Human Trafficking Hotline (2018).

**Table S17. Links between religious factors (Pew) and gender bias in cultural tightness (Wave 2).**

| Variables | Model 1 | Model 2 | Model 3 | Model 4 | Model 5 | Model 6 | Model 7 | Model 8 | Model 9 | Model 10 | Model 11 | Model 12 |
| --- | --- | --- | --- | --- | --- | --- | --- | --- | --- | --- | --- | --- |
| Percentage of adults who are highly religious^†^ | **5.61^***^ (1.07)** | **4.63^***^ (1.22)** |  |  |  |  |  |  |  |  |  |  |
| Importance of religion |  |  | **5.64^***^ (1.07)** | **4.70^***^ (1.25)** |  |  |  |  |  |  |  |  |
| Frequency of prayer |  |  |  |  | **6.16^***^ (1.25)** | **4.98^***^ (1.36)** |  |  |  |  |  |  |
| Worship attendance |  |  |  |  |  |  | **8.77^***^ (1.42)** | **7.55^***^ (1.55)** |  |  |  |  |
| Belief in God |  |  |  |  |  |  |  |  | **5.95^***^ (1.25)** | **4.71^**^ (1.41)** |  |  |
| Religious belief |  |  |  |  |  |  |  |  |  |  | **8.86^***^ (2.14)** | **7.42^**^ (2.26)** |
| GDP per capita (log): 1977-2020 |  | -1.17 (0.74) |  | -1.08 (0.75) |  | -1.31^+^ (0.73) |  | -1.14^+^ (0.67) |  | -1.22 (0.77) |  | -1.88^*^ (0.70) |
| Gender imbalance in population: 1970-2020 (more men than women) |  | -0.83 (3.59) |  | -0.37 (3.62) |  | -1.33 (3.61) |  | -1.21 (3.33) |  | -1.69 (3.68) |  | 1.50 (3.88) |
| Constant | -0.07 (0.60) | 12.75 (8.09) | 0.03 (0.57) | 11.84 (8.27) | -0.34 (0.68) | 14.08^+^ (8.06) | -0.13 (0.52) | 12.22 (7.30) | -0.77 (0.80) | 12.77 (8.56) | -3.76^*^ (1.64) | 17.12^*^ (7.97) |
| *N* | 50 | 50 | 50 | 50 | 50 | 50 | 50 | 50 | 50 | 50 | 50 | 50 |
| *R*^2^ | 0.36 | 0.40 | 0.37 | 0.40 | 0.34 | 0.39 | 0.44 | 0.48 | 0.32 | 0.37 | 0.26 | 0.36 |
| *F* | 27.44 | 10.26 | 27.95 | 10.08 | 24.51 | 9.77 | 37.99 | 14.23 | 22.70 | 8.83 | 17.17 | 8.71 |

^+^*P* < 0.1; ^*^*P* < 0.05; ^**^*P* < 0.01; ^***^*P* < 0.001. Results from ordinary least squares regressions. Unstandardized regression coefficients are reported. Standard errors in parentheses.

^†^ Religious data in this table were obtained and computed from the Pew Research Center (2014).

**Table S18. Links between religious factors (Pew, breakdown of key religious affiliation) and gender bias in cultural tightness (Wave 2).**

| Variables | Model 1 | Model 2 | Model 3 | Model 4 | Model 5 | Model 6 | Model 7 | Model 8 | Model 9 | Model 10 | Model 11 | Model 12 | Model 13 | Model 14 | Model 15 | Model 16 | Model 17 | Model 18 |
| --- | --- | --- | --- | --- | --- | --- | --- | --- | --- | --- | --- | --- | --- | --- | --- | --- | --- | --- |
| Non-religious | **-8.69^***^ (2.20)** | **-7.22^**^ (2.32)** |  |  |  |  |  |  |  |  |  |  |  |  |  |  |  |  |
| Buddhists^†‡§^ |  |  | **-26.56^*^ (11.59)** | **-17.34 (11.58)** |  |  |  |  |  |  |  |  |  |  |  |  |  |  |
| Catholics |  |  |  |  | **-4.57^**^ (1.50)** | **-3.27^+^ (1.72)** |  |  |  |  |  |  |  |  |  |  |  |  |
| Evangelical Protestants |  |  |  |  |  |  | **3.70^**^ (1.20)** | **2.16 (1.37)** |  |  |  |  |  |  |  |  |  |  |
| Hindus |  |  |  |  |  |  |  |  | **-58.30^**^ (20.09)** | **-52.67^*^ (23.87)** |  |  |  |  |  |  |  |  |
| Black Protestants |  |  |  |  |  |  |  |  |  |  | **5.68^*^ (2.36)** | **4.90^+^ (2.49)** |  |  |  |  |  |  |
| Jews |  |  |  |  |  |  |  |  |  |  |  |  | **-25.13^**^ (9.12)** | **-27.98^*^ (11.16)** |  |  |  |  |
| Mainline Protestants |  |  |  |  |  |  |  |  |  |  |  |  |  |  | **-0.21 (2.47)** | **-1.06 (2.24)** |  |  |
| Mormons |  |  |  |  |  |  |  |  |  |  |  |  |  |  |  |  | **3.33^+^ (1.72)** | **3.73^*^ (1.65)** |
| GDP per capita (log): 1977-2020 |  | -1.93^**^ (0.71) |  | -2.18^**^ (0.76) |  | -1.46 (0.88) |  | -1.67^+^ (0.87) |  | -1.25 (0.89) |  | -2.35^**^ (0.73) |  | -1.08 (0.88) |  | -2.42^**^ (0.77) |  | -2.05^**^ (0.74) |
| Gender imbalance in population: 1970-2020 (more men than women) |  | 1.38 (3.93) |  | -1.00 (4.13) |  | -4.91 (4.11) |  | -2.93 (3.98) |  | -7.20 (4.40) |  | 1.41 (4.43) |  | -9.39^+^ (4.68) |  | -2.67 (4.07) |  | -6.03 (4.15) |
| Constant | 5.01^***^ (0.52) | 24.97^**^ (7.39) | 3.19^***^ (0.16) | 26.06^**^ (7.98) | 3.87^***^ (0.31) | 18.79^*^ (9.14) | 2.04^***^ (0.34) | 19.93^*^ (9.35) | 3.21^***^ (0.15) | 16.05^+^ (9.38) | 2.68^***^ (0.19) | 27.48^**^ (7.75) | 3.30^***^ (0.17) | 14.35 (9.31) | 3.03^***^ (0.43) | 28.50^**^ (8.13) | 2.90^***^ (0.15) | 24.24^**^ (7.83) |
| *N* | 50 | 50 | 50 | 50 | 50 | 50 | 50 | 50 | 50 | 50 | 50 | 50 | 50 | 50 | 50 | 50 | 50 | 50 |
| *R*^2^ | 0.24 | 0.35 | 0.10 | 0.25 | 0.16 | 0.27 | 0.16 | 0.25 | 0.15 | 0.29 | 0.11 | 0.27 | 0.14 | 0.31 | 0.00 | 0.22 | 0.07 | 0.29 |
| *F* | 15.55 | 8.25 | 5.25 | 5.09 | 9.29 | 5.66 | 9.45 | 5.19 | 8.42 | 6.20 | 5.78 | 5.77 | 7.59 | 6.79 | 0.01 | 4.23 | 3.76 | 6.30 |

^+^*P* < 0.1; ^*^*P* < 0.05; ^**^*P* < 0.01; ^***^*P* < 0.001. Results from ordinary least squares regressions. Unstandardized regression coefficients are reported. Standard errors in parentheses.

^†^ Religious data in this table were obtained and computed from the Pew Research Center (2014).

^‡^ Some other minor religions (e.g., Jehovah’s Witness) were considered but not significant.

^§^ To make the variables continuous, the values that were less than 1% were replaced by zero.

**Table S19. Links between religious factors (Gallup) and gender bias in cultural tightness (Wave 2).**

| Variables | Model 1 | Model 2 | Model 3 | Model 4 | Model 5 | Model 6 |
| --- | --- | --- | --- | --- | --- | --- |
| Percentage of adults who are very religious^†^ | **7.48^***^ (1.12)** | **6.70^***^ (1.31)** |  |  |  |  |
| Percentage of adults who are moderately religious |  |  | **1.29 (4.71)** | **1.57 (4.58)** |  |  |
| Percentage of adults who are non-religious |  |  |  |  | **-6.59^***^ (1.07)** | **-5.74^***^ (1.22)** |
| GDP per capita (log): 1977-2020 |  | -0.67 (0.70) |  | -2.43^**^ (0.78) |  | -1.07 (0.69) |
| Gender imbalance in population: 1970-2020 (more men than women) |  | -1.51 (3.27) |  | -2.12 (4.37) |  | 0.13 (3.41) |
| Constant | 0.08 (0.45) | 7.37 (7.61) | 2.63^+^ (1.35) | 28.03^**^ (8.06) | 5.16^***^ (0.37) | 16.14^*^ (7.09) |
| *N* | 50 | 50 | 50 | 50 | 50 | 50 |
| *R*^2^ | 0.48 | 0.50 | 0.00 | 0.21 | 0.44 | 0.47 |
| *F* | 44.59 | 15.15 | 0.07 | 4.19 | 37.62 | 13.51 |

^+^*P* < 0.1; ^*^*P* < 0.05; ^**^*P* < 0.01; ^***^*P* < 0.001. Results from ordinary least squares regressions. Unstandardized regression coefficients are reported. Standard errors in parentheses.

^†^ Religious data in this table were obtained and computed from the Gallup Daily Tracking (2016).

**Table S20. Links between political ideology and gender bias in cultural tightness (Wave 2).**

| Variables | Model 1 | Model 2 | Model 3 | Model 4 | Model 5 | Model 6 |
| --- | --- | --- | --- | --- | --- | --- |
| Percentage of people having conservative political ideology^†^ | **11.06^***^**  **(1.49)** | **11.98^***^**  **(1.78)** |  |  |  |  |
| Percentage of Republicans in the U.S. Senate |  |  | **1.41^***^**  **(0.24)** | **1.35^***^**  **(0.25)** |  |  |
| Percentage of Republicans in the House of Representatives |  |  |  |  | **1.62^***^**  **(0.33)** | **1.69^***^**  **(0.34)** |
| GDP per capita (log): 1977-2020 |  | 0.45  (0.68) |  | -0.86  (0.66) |  | -0.81  (0.69) |
| Gender imbalance in population: 1970-2020 (more men than women) |  | -8.36^**^  (3.02) |  | -7.40^*^  (3.30) |  | -9.44^*^  (3.56) |
| Constant | -1.13^+^  (0.56) | -6.47  (7.67) | 2.26^***^  (0.17) | 11.13  (7.01) | 2.15^***^  (0.21) | 10.29  (7.41) |
| *N* | 50 | 50 | 50 | 50 | 50 | 50 |
| *R*^2^ | 0.54 | 0.60 | 0.41 | 0.52 | 0.34 | 0.49 |
| *F* | 55.35 | 23.36 | 33.82 | 16.67 | 24.83 | 14.64 |

^+^*P* < 0.1; ^*^*P* < 0.05; ^**^*P* < 0.01; ^***^*P* < 0.001. Results from ordinary least squares regressions. Unstandardized regression coefficients are reported. Standard errors in parentheses.

^†^ Percentage of people having conservative political ideology was obtained and computed from the Pew Research Center (2014), while percentages of republicans in the U.S. Senate and in the House of Representatives were obtained and computed from the Biographical Directory of the United States Congress (2019-2021).

**Table S21. Links between gender-related threats and gender bias in cultural tightness (Wave 2).**

| Variables | Model 1 | Model 2 | Model 3 | Model 4 | Model 5 | Model 6 | Model 7 | Model 8 | Model 9 | Model 10 | Model 11 | Model 12 | Model 13 | Model 14 | Model 15 | Model 16 |
| --- | --- | --- | --- | --- | --- | --- | --- | --- | --- | --- | --- | --- | --- | --- | --- | --- |
| State sexism belief i^†^ | **4.01^***^ (0.94)** | **3.51^***^ (0.92)** |  |  |  |  |  |  |  |  |  |  |  |  |  |  |
| State sexism belief ii |  |  | **4.27^***^ (0.78)** | **4.48^***^ (1.02)** |  |  |  |  |  |  |  |  |  |  |  |  |
| Percentage of people favoring non-discrimination LGBT protection |  |  |  |  | **-7.87^*^**  **(3.05)** | **-7.09^*^**  **(3.16)** |  |  |  |  |  |  |  |  |  |  |
| Percentage of people viewing homosexuality as acceptable |  |  |  |  |  |  | **-6.53^***^**  **(1.19)** | **-5.85^***^**  **(1.44)** |  |  |  |  |  |  |  |  |
| Percentage of male-dominated industries: 2001-2018 |  |  |  |  |  |  |  |  | **12.22^**^ (3.33)** | **12.48^**^ (3.70)** |  |  |  |  |  |  |
| Sexual violence against women |  |  |  |  |  |  |  |  |  |  | **-0.09^*^**  **(0.04)** | **-0.08**  **(0.05)** |  |  |  |  |
| Relative domestic violence |  |  |  |  |  |  |  |  |  |  |  |  | **0.34**  **(0.39)** | **0.21**  **(0.36)** |  |  |
| Relative human trafficking |  |  |  |  |  |  |  |  |  |  |  |  |  |  | **-0.34^+^**  **(0.19)** | **-0.26**  **(0.18)** |
| GDP per capita (log): 1977-2020 |  | -1.73^*^ (0.73) |  | 0.12 (0.86) |  | -1.67^*^  (0.79) |  | -0.55  (0.79) |  | -1.01 (0.80) |  | -1.87^+^  (1.01) |  | -2.48^**^  (0.75) |  | -2.15^**^  (0.76) |
| Gender imbalance in population: 1970-2020 (more men than women) |  | -3.33 (4.09) |  | -4.57 (4.17) |  | -6.17  (4.18) |  | -4.46  (3.53) |  | -8.74^*^ (4.08) |  | 2.20  (5.59) |  | -2.41  (3.98) |  | -3.40  (4.02) |
| Constant | -5.18^**^ (1.92) | 13.95 (8.30) | -9.21^***^ (2.25) | -11.22 (11.17) | 8.54^***^  (2.15) | 25.38^**^  (7.74) | 6.95^***^  (0.73) | 12.15  (7.94) | 0.33 (0.74) | 10.56 (8.87) | 5.54^***^  (1.15) | 24.86^*^  (10.66) | 2.55^***^  (0.56) | 28.73^**^  (7.96) | 3.67^***^  (0.40) | 26.04^**^  (8.00) |
| *N* | 49 | 49 | 48 | 48 | 50 | 50 | 50 | 50 | 50 | 50 | 41 | 41 | 49 | 49 | 50 | 50 |
| *R*^2^ | 0.28 | 0.37 | 0.39 | 0.41 | 0.12 | 0.29 | 0.38 | 0.42 | 0.22 | 0.37 | 0.11 | 0.18 | 0.02 | 0.24 | 0.06 | 0.25 |
| *F* | 18.29 | 8.99 | 29.92 | 10.20 | 6.68 | 6.27 | 29.95 | 11.13 | 13.49 | 8.95 | 4.61 | 2.79 | 0.76 | 4.86 | 3.22 | 5.03 |

^+^*P* < 0.1; ^*^*P* < 0.05; ^**^*P* < 0.01; ^***^*P* < 0.001. Results from ordinary least squares regressions. Unstandardized regression coefficients are reported. Standard errors in parentheses.

^†^ State sexism belief i was obtained and computed from the World Value Survey (2017), whereas state sexism belief ii was obtained and computed from the DDB Needham Life Style Survey (1975-1998). Percentage of people favoring non-discrimination LGBT protection was obtained and computed from the Research on LGBT in PRRI American Values Atlas (2019). Percentage of people viewing homosexuality as acceptable was obtained and computed from the Pew Research Center (2014). Percentage of male-dominated industries was obtained and computed from the NAICS Industry data from the Bureau of Economic Analysis (2001-2018). Sexual violence against women and relative domestic violence were obtained and computed from the National Intimate Partner and Sexual Violence Survey (2010). Relative human trafficking was obtained and computed from the National Human Trafficking Hotline (2018).

**Table S22. Links between gender bias in cultural tightness (Wave 1) and gender inequality in business leadership.**

| Variables | Gender inequality in boards of publicly traded companies^†^ | | Gender inequality in CEOs of publicly traded companies | | Gender inequality in management occupations: total | | Gender inequality in management occupations: top executives | | Gender inequality in business leadership (aggregated)^‡^ | |
| --- | --- | --- | --- | --- | --- | --- | --- | --- | --- | --- |
|  | Model 1 | Model 2 | Model 3 | Model 4 | Model 5 | Model 6 | Model 7 | Model 8 | Model 9 | Model 10 |
| Gender bias in cultural tightness | **0.03^**^ (0.01)** | **0.03^*^ (0.01)** | **0.02^+^ (0.01)** | **0.03 (0.02)** | **0.03^***^ (0.01)** | **0.03^*^ (0.01)** | **0.02^***^ (0.01)** | **0.02^***^ (0.01)** | **0.28^***^ (0.07)** | **0.27^***^ (0.07)** |
| GDP per capita (log) |  | 0.18^***^ (0.04) |  | 0.45^***^ (0.08) |  | -0.05 (0.03) |  | 0.03 (0.03) |  | 0.60^*^ (0.23) |
| Gender imbalance in population (more men than women) |  | 0.51^+^ (0.28) |  | 0.88^+^ (0.49) |  | 1.17^***^ (0.26) |  | 0.08 (0.16) |  | 7.24^***^ (1.64) |
| State cultural tightness |  | 0.00^*^ (0.00) |  | 0.00^**^ (0.00) |  | 0.00 (0.00) |  | 0.00 (0.00) |  | 0.01^+^ (0.01) |
| Constant | 0.55^***^ (0.03) | -1.46^**^ (0.44) | 0.84^***^ (0.04) | -4.29^***^ (0.88) | 0.23^***^ (0.03) | 0.78^*^ (0.37) | 0.50^***^ (0.02) | 0.12 (0.32) | -1.54^***^ (0.21) | -8.41^**^ (2.66) |
| Year | 2007-2020 | 2007-2020 | 2007-2020 | 2007-2020 | 2005-2019 | 2005-2019 | 2005-2019 | 2005-2019 | 2007-2019 | 2007-2019 |
| *N* | 676 | 676 | 664 | 664 | 750 | 750 | 750 | 750 | 629 | 629 |

^+^*P* < 0.1; ^*^*P* < 0.05; ^**^*P* < 0.01; ^***^*P* < 0.001. Results from hierarchical linear modeling. Unstandardized regression coefficients are reported. Standard errors in parentheses.

^†^ Gender inequality in boards and CEOs of publicly traded companies were obtained and computed from the Institutional Shareholder Services (ISS) and Compustat (2007-2020), while gender inequality in management occupations, including top executives, were obtained and computed from the U.S. Census Bureau’s American Community Survey (2005-2019).

^‡^ Gender inequality in business leadership was calculated by taking an average of the standardized gender inequality in boards of publicly traded companies and total management occupations.

**Table S23. Links between gender bias in cultural tightness (Wave 1) and gender inequality in political leadership.**

| Variables | Gender inequality in U.S. Senators^†^ | | Gender inequality in U.S. Representatives | | Gender inequality in State Senators | | Gender inequality in State Representatives | | Gender inequality in State Governors | | Gender inequality in political leadership (aggregated)^‡^ | |
| --- | --- | --- | --- | --- | --- | --- | --- | --- | --- | --- | --- | --- |
|  | Model 1 | Model 2 | Model 3 | Model 4 | Model 5 | Model 6 | Model 7 | Model 8 | Model 9 | Model 10 | Model 11 | Model 12 |
| Gender bias in cultural tightness | **0.05^*^ (0.02)** | **0.07^*^ (0.03)** | **0.05^*^ (0.02)** | **0.02 (0.02)** | **0.07^***^ (0.01)** | **0.04^**^ (0.01)** | **0.07^***^ (0.01)** | **0.04^**^ (0.01)** | **0.65^*^ (0.29)** | **0.73^*^ (0.35)** | **0.28^***^ (0.05)** | **0.17^**^ (0.05)** |
| GDP per capita (log) |  | 0.05 (0.08) |  | 0.03 (0.06) |  | 0.02 (0.03) |  | 0.11^***^ (0.03) |  | 1.56 (1.21) |  | 0.23^*^ (0.12) |
| Gender imbalance in population (more men than women) |  | 0.08 (0.57) |  | 0.68 (0.43) |  | 0.34 (0.22) |  | 0.01 (0.19) |  | 4.62 (7.73) |  | 0.94 (0.83) |
| State cultural tightness |  | -0.00 (0.00) |  | 0.01^***^ (0.00) |  | 0.00^***^ (0.00) |  | 0.01^***^ (0.00) |  | 0.01 (0.03) |  | 0.02^***^ (0.00) |
| Constant | 0.84^***^ (0.08) | 0.36 (0.88) | 0.79^***^ (0.07) | 0.32 (0.66) | 0.74^***^ (0.04) | 0.43 (0.33) | 0.69^***^ (0.04) | -0.62^*^ (0.27) | 2.39^*^ (1.17) | -13.52 (12.43) | -0.11 (0.17) | -3.07^*^ (1.22) |
| Year | 1977-2020 | 1977-2020 | 1977-2020 | 1977-2020 | 1977-2020 | 1977-2020 | 1977-2020 | 1977-2020 | 1977-2020 | 1977-2020 | 1977-2020 | 1977-2020 |
| *N* | 2117 | 2117 | 2143 | 2143 | 2049 | 2049 | 2008 | 2008 | 2200 | 2200 | 1915 | 1915 |

^+^*P* < 0.1; ^*^*P* < 0.05; ^**^*P* < 0.01; ^***^*P* < 0.001. Results from hierarchical linear modeling, and hierarchical logistic modeling (Models 11 and 12). Unstandardized regression coefficients are reported. Standard errors in parentheses.

^†^ Gender inequality in U.S. Senators and Representatives (1901-2020), and state Senators, Representatives (1975-2020), and state Governors (1901-2020) were obtained and computed from the Center for American Women and Politics (CAWP).

^‡^ Gender inequality in political leadership was calculated by taking an average of the standardized gender inequality in U.S. Senators and Representatives, and State Senators, Representatives, and Governors.

**Table S24. Links between gender bias in cultural tightness (Wave 1) and gender inequality in innovation (patent inventors and STEM occupations).**

| Variables | Gender inequality in patent inventors: utility^†^ | | Gender inequality in patent inventors: design | | Gender inequality in patent inventors: plant | | Gender inequality in patent inventors (aggregate of design, plant, and utility patents) | | Gender inequality in STEM occupations | |
| --- | --- | --- | --- | --- | --- | --- | --- | --- | --- | --- |
|  | Model 1 | Model 2 | Model 3 | Model 4 | Model 5 | Model 6 | Model 7 | Model 8 | Model 9 | Model 10 |
| Gender bias in cultural tightness | **0.01^***^ (0.00)** | **0.01^***^ (0.00)** | **-0.01 (0.01)** | **-0.01 (0.01)** | **-0.06 (0.08)** | **-0.03 (0.11)** | **0.01^**^ (0.00)** | **0.01^*^ (0.00)** | **0.01^**^ (0.00)** | **0.02^**^ (0.00)** |
| GDP per capita (log) |  | -0.07^***^ (0.01) |  | -0.25^***^ (0.07) |  | 0.91^+^ (0.54) |  | -0.10^***^ (0.02) |  | -0.03 (0.02) |
| Gender imbalance in population (more men than women) |  | 0.22^*^ (0.09) |  | -0.39 (0.38) |  | 5.23 (3.62) |  | 0.24^*^ (0.10) |  | 0.43^***^ (0.13) |
| State cultural tightness |  | -0.00^+^ (0.00) |  | -0.00^*^ (0.00) |  | 0.01 (0.01) |  | -0.00^*^ (0.00) |  | -0.00 (0.00) |
| Constant | 0.85^***^ (0.01) | 1.69^***^ (0.15) | 0.87^***^ (0.04) | 3.75^***^ (0.76) | 0.51^+^ (0.29) | -9.90 (6.19) | 0.85^***^ (0.01) | 2.03^***^ (0.17) | 0.62^***^ (0.01) | 0.96^***^ (0.22) |
| Year | 2008-2020 | 2008-2020 | 2008-2020 | 2008-2020 | 2008-2020 | 2008-2020 | 2008-2020 | 2008-2020 | 2005-2019 | 2005-2019 |
| *N* | 650 | 650 | 626 | 626 | 187 | 187 | 650 | 650 | 750 | 750 |

^+^*P* < 0.1; ^*^*P* < 0.05; ^**^*P* < 0.01; ^***^*P* < 0.001. Results from hierarchical linear modeling. Unstandardized regression coefficients are reported. Standard errors in parentheses.

^†^ Gender inequality in patent inventors were obtained and computed from the PatentsView reported by the U.S. Patent and Trademark Office (USPTO; 2008-2020), while gender inequality in STEM was obtained and computed from the U.S. Census Bureau’s American Community Survey (ACS; 2005-2019).

**Table S25. Links between gender bias in cultural tightness (Wave 1) and gender inequality in higher education attainment.**

| Variables | Gender inequality in attainment of bachelor’s degrees^†^ | | Gender inequality in attainment of master’s degrees | | Gender inequality in attainment of professional degrees | | Gender inequality in attainment of doctoral degrees | |
| --- | --- | --- | --- | --- | --- | --- | --- | --- |
|  | Model 1 | Model 2 | Model 3 | Model 4 | Model 5 | Model 6 | Model 7 | Model 8 |
| Gender bias in cultural tightness | **-0.02^+^ (0.01)** | **-0.00 (0.01)** | **-0.03 (0.03)** | **0.01 (0.03)** | **0.02 (0.02)** | **0.04^+^ (0.02)** | **0.01^**^ (0.00)** | **0.01^**^ (0.00)** |
| GDP per capita (log) |  | 0.02 (0.05) |  | -0.18 (0.12) |  | 0.10 (0.11) |  | -0.00 (0.01) |
| Gender imbalance in population (more men than women) |  | 0.21 (0.30) |  | 0.93 (0.75) |  | 1.06^+^ (0.59) |  | 0.16^**^ (0.05) |
| State cultural tightness |  | -0.00 (0.00) |  | -0.01^**^ (0.00) |  | -0.00 (0.00) |  | -0.00 (0.00) |
| Constant | -0.12^***^ (0.03) | -0.28 (0.55) | -0.30^***^ (0.09) | 1.87 (1.30) | 0.10 (0.08) | -0.89 (1.19) | 0.91^***^ (0.01) | 0.96^***^ (0.10) |
| Year | 2003-2020 | 2003-2020 | 2003-2020 | 2003-2020 | 2003-2020 | 2003-2020 | 2003-2020 | 2003-2020 |
| *N* | 900 | 900 | 900 | 900 | 898 | 898 | 898 | 898 |

^+^*P* < 0.1; ^*^*P* < 0.05; ^**^*P* < 0.01; ^***^*P* < 0.001. Results from hierarchical linear modeling. Unstandardized regression coefficients are reported. Standard errors in parentheses.

^†^ Gender inequality in higher education was obtained and computed from the Current Population Survey (CPS; 2003-2020) reported by the U.S. Census Bureau.

**Table S26. Links between gender bias in cultural tightness (Wave 1) and gender inequality in entrepreneurship.**

| Variables | Gender inequality in number of startup firms | | Gender inequality in all firm ownership | |
| --- | --- | --- | --- | --- |
|  | Model 1 | Model 2 | Model 3 | Model 4 |
| Gender bias in cultural tightness | **0.02^***^ (0.01)** | **0.03^***^ (0.01)** | **0.02^**^ (0.01)** | **0.02^**^ (0.01)** |
| GDP per capita (log) |  | 0.08^*^ (0.03) |  | 0.11^**^ (0.04) |
| Gender imbalance in population (more men than women) |  | -0.64^***^ (0.18) |  | -0.19 (0.24) |
| State cultural tightness |  | 0.00 (0.00) |  | 0.00 (0.00) |
| Constant | 0.62^***^ (0.02) | -0.26 (0.35) | 0.30^***^ (0.02) | -0.91^*^ (0.41) |
| Year | 2014-2016 | 2014-2016 | 2002-2012 | 2002-2012 |
| *N* | 150 | 150 | 150 | 150 |

^+^*P* < 0.1; ^*^*P* < 0.05; ^**^*P* < 0.01; ^***^*P* < 0.001. Results from hierarchical linear modeling. Unstandardized regression coefficients are reported. Standard errors in parentheses.

^†^ Gender inequality in number of startup firms was obtained and computed from the Annual Survey of Entrepreneurs (ASE; 2014-2016), while gender inequality in all firm ownership was obtained and computed from the Survey of Business Owners (SBO; 2002-2012) from the U.S. Census Bureau.

**Table S27. Links between gender bias in cultural tightness (Wave 2) and gender inequality in business leadership.**

| Variables | Gender inequality in boards of publicly traded companies^†^ | | Gender inequality in CEOs of publicly traded companies | | Gender inequality in management occupations: total | | Gender inequality in management occupations: top executives | | Gender inequality in business leadership (aggregated)^‡^ | |
| --- | --- | --- | --- | --- | --- | --- | --- | --- | --- | --- |
|  | Model 1 | Model 2 | Model 3 | Model 4 | Model 5 | Model 6 | Model 7 | Model 8 | Model 9 | Model 10 |
| Gender bias in cultural tightness | **0.03^**^ (0.01)** | **0.03^*^ (0.01)** | **0.03^*^ (0.01)** | **0.04^*^ (0.02)** | **0.03^***^ (0.01)** | **0.03^**^ (0.01)** | **0.03^***^ (0.00)** | **0.03^***^ (0.01)** | **0.29^***^ (0.07)** | **0.31^***^ (0.07)** |
| GDP per capita (log) |  | 0.18^***^ (0.04) |  | 0.45^***^ (0.08) |  | -0.05 (0.03) |  | 0.03 (0.03) |  | 0.61^**^ (0.23) |
| Gender imbalance in population (more men than women) |  | 0.55^+^ (0.28) |  | 0.88^+^ (0.47) |  | 1.19^***^ (0.25) |  | 0.12 (0.16) |  | 7.62^***^ (1.59) |
| State cultural tightness |  | 0.00 (0.00) |  | 0.00^*^ (0.00) |  | 0.00 (0.00) |  | 0.00 (0.00) |  | 0.01 (0.01) |
| Constant | 0.55^***^ (0.04) | -1.44^**^ (0.44) | 0.82^***^ (0.04) | -4.25^***^ (0.86) | 0.23^***^ (0.03) | 0.78^*^ (0.36) | 0.50^***^ (0.02) | 0.12 (0.31) | -1.55^***^ (0.21) | -8.41^**^ (2.61) |
| Year | 2007-2020 | 2007-2020 | 2007-2020 | 2007-2020 | 2005-2019 | 2005-2019 | 2005-2019 | 2005-2019 | 2007-2019 | 2007-2019 |
| *N* | 676 | 676 | 664 | 664 | 750 | 750 | 750 | 750 | 629 | 629 |

^+^*P* < 0.1; ^*^*P* < 0.05; ^**^*P* < 0.01; ^***^*P* < 0.001. Results from hierarchical linear modeling. Unstandardized regression coefficients are reported. Standard errors in parentheses.

^†^ Gender inequality in boards and CEOs of publicly traded companies were obtained and computed from the Institutional Shareholder Services (ISS) and Compustat (2007-2020), while gender inequality in management occupations, including top executives, were obtained and computed from the U.S. Census Bureau’s American Community Survey (2005-2019).

^‡^ Gender inequality in business leadership was calculated by taking an average of the standardized gender inequality in boards of publicly traded companies and total management occupations.

**Table S28. Links between gender bias in cultural tightness (Wave 2) and gender inequality in political leadership.**

| Variables | Gender inequality in U.S. Senators^†^ | | Gender inequality in U.S. Representatives | | Gender inequality in State Senators | | Gender inequality in State Representatives | | Gender inequality in State Governors | | Gender inequality in political leadership (aggregated)^‡^ | |
| --- | --- | --- | --- | --- | --- | --- | --- | --- | --- | --- | --- | --- |
|  | Model 1 | Model 2 | Model 3 | Model 4 | Model 5 | Model 6 | Model 7 | Model 8 | Model 9 | Model 10 | Model 11 | Model 12 |
| Gender bias in cultural tightness | **0.04^+^ (0.02)** | **0.06^+^ (0.03)** | **0.05^*^ (0.02)** | **0.01 (0.02)** | **0.07^***^ (0.01)** | **0.05^***^ (0.01)** | **0.07^***^ (0.01)** | **0.04^**^ (0.01)** | **0.57^*^ (0.29)** | **0.69^+^ (0.38)** | **0.30^***^ (0.05)** | **0.17^**^ (0.06)** |
| GDP per capita (log) |  | 0.05 (0.09) |  | 0.03 (0.06) |  | 0.02 (0.03) |  | 0.11^***^ (0.03) |  | 1.47 (1.21) |  | 0.23^+^ (0.12) |
| Gender imbalance in population (more men than women) |  | 0.15 (0.57) |  | 0.70 (0.43) |  | 0.35 (0.22) |  | 0.03 (0.19) |  | 5.58 (7.83) |  | 1.04 (0.83) |
| State cultural tightness |  | -0.00 (0.00) |  | 0.01^**^ (0.00) |  | 0.00^***^ (0.00) |  | 0.01^***^ (0.00) |  | 0.00 (0.03) |  | 0.02^***^ (0.00) |
| Constant | 0.87^***^ (0.09) | 0.45 (0.88) | 0.79^***^ (0.07) | 0.36 (0.65) | 0.71^***^ (0.04) | 0.42 (0.32) | 0.67^***^ (0.04) | -0.60^*^ (0.27) | 2.62^*^ (1.17) | -12.25 (12.46) | -0.16 (0.16) | -2.94^*^ (1.21) |
| Year | 1977-2020 | 1977-2020 | 1977-2020 | 1977-2020 | 1977-2020 | 1977-2020 | 1977-2020 | 1977-2020 | 1977-2020 | 1977-2020 | 1977-2020 | 1977-2020 |
| *N* | 2117 | 2117 | 2143 | 2143 | 2049 | 2049 | 2008 | 2008 | 2200 | 2200 | 1915 | 1915 |

^+^*P* < 0.1; ^*^*P* < 0.05; ^**^*P* < 0.01; ^***^*P* < 0.001. Results from hierarchical linear modeling, and hierarchical logistic modeling (Models 11 and 12). Unstandardized regression coefficients are reported. Standard errors in parentheses.

^†^ Gender inequality in U.S. Senators and Representatives (1901-2020), and state Senators, Representatives (1975-2020), and state Governors (1901-2020) were obtained and computed from the Center for American Women and Politics (CAWP).

^‡^ Gender inequality in political leadership was calculated by taking an average of the standardized gender inequality in U.S. Senators and Representatives, and State Senators, Representatives, and Governors.

**Table S29. Links between gender bias in cultural tightness (Wave 2) and gender inequality in innovation (patent inventors and STEM occupations).**

| Variables | Gender inequality in patent inventors: utility^†^ | | Gender inequality in patent inventors: design | | Gender inequality in patent inventors: plant | | Gender inequality in patent inventors (aggregate of design, plant, and utility patents) | | Gender inequality in STEM occupations | |
| --- | --- | --- | --- | --- | --- | --- | --- | --- | --- | --- |
|  | Model 1 | Model 2 | Model 3 | Model 4 | Model 5 | Model 6 | Model 7 | Model 8 | Model 9 | Model 10 |
| Gender bias in cultural tightness | **0.01^***^ (0.00)** | **0.01^***^ (0.00)** | **-0.00 (0.01)** | **0.00 (0.01)** | **0.02 (0.08)** | **0.13 (0.11)** | **0.01^**^ (0.00)** | **0.01^**^ (0.00)** | **0.01^*^ (0.00)** | **0.01^*^ (0.01)** |
| GDP per capita (log) |  | -0.07^***^ (0.01) |  | -0.24^***^ (0.07) |  | 1.08^*^ (0.52) |  | -0.10^***^ (0.01) |  | -0.03 (0.02) |
| Gender imbalance in population (more men than women) |  | 0.24^**^ (0.08) |  | -0.45 (0.38) |  | 5.95^+^ (3.57) |  | 0.26^**^ (0.09) |  | 0.46^***^ (0.13) |
| State cultural tightness |  | -0.00^*^ (0.00) |  | -0.00^**^ (0.00) |  | 0.01 (0.01) |  | -0.00^**^ (0.00) |  | -0.00 (0.00) |
| Constant | 0.85^***^ (0.01) | 1.70^***^ (0.15) | 0.84^***^ (0.04) | 3.57^***^ (0.76) | 0.26 (0.27) | -11.82^*^ (5.88) | 0.85^***^ (0.01) | 2.01^***^ (0.17) | 0.63^***^ (0.02) | 0.99^***^ (0.23) |
| Year | 2008-2020 | 2008-2020 | 2008-2020 | 2008-2020 | 2008-2020 | 2008-2020 | 2008-2020 | 2008-2020 | 2005-2019 | 2005-2019 |
| *N* | 650 | 650 | 626 | 626 | 187 | 187 | 650 | 650 | 750 | 750 |

^+^*P* < 0.1; ^*^*P* < 0.05; ^**^*P* < 0.01; ^***^*P* < 0.001. Results from hierarchical linear modeling. Unstandardized regression coefficients are reported. Standard errors in parentheses.

^†^ Gender inequality in patent inventors were obtained and computed from the PatentsView reported by the U.S. Patent and Trademark Office (USPTO; 2008-2020), while gender inequality in STEM was obtained and computed from the U.S. Census Bureau’s American Community Survey (ACS; 2005-2019).

**Table S30. Links between gender bias in cultural tightness (Wave 2) and gender inequality in higher education attainment.**

| Variables | Gender inequality in attainment of bachelor’s degrees^†^ | | Gender inequality in attainment of master’s degrees | | Gender inequality in attainment of professional degrees | | Gender inequality in attainment of doctoral degrees | |
| --- | --- | --- | --- | --- | --- | --- | --- | --- |
|  | Model 1 | Model 2 | Model 3 | Model 4 | Model 5 | Model 6 | Model 7 | Model 8 |
| Gender bias in cultural tightness | **-0.01 (0.01)** | **0.00 (0.01)** | **-0.03 (0.03)** | **0.02 (0.03)** | **0.01 (0.02)** | **0.03 (0.02)** | **0.01^**^ (0.00)** | **0.01^**^ (0.00)** |
| GDP per capita (log) |  | 0.02 (0.05) |  | -0.17 (0.11) |  | 0.08 (0.11) |  | -0.00 (0.01) |
| Gender imbalance in population (more men than women) |  | 0.18 (0.30) |  | 0.92 (0.74) |  | 1.16^*^ (0.59) |  | 0.18^***^ (0.05) |
| State cultural tightness |  | -0.00 (0.00) |  | -0.01^**^ (0.00) |  | -0.00 (0.00) |  | -0.00 (0.00) |
| Constant | -0.12^***^ (0.03) | -0.34 (0.55) | -0.30^***^ (0.09) | 1.79 (1.29) | 0.14^+^ (0.08) | -0.72 (1.19) | 0.91^***^ (0.01) | 0.97^***^ (0.09) |
| Year | 2003-2020 | 2003-2020 | 2003-2020 | 2003-2020 | 2003-2020 | 2003-2020 | 2003-2020 | 2003-2020 |
| *N* | 900 | 900 | 900 | 900 | 898 | 898 | 898 | 898 |

^+^*P* < 0.1; ^*^*P* < 0.05; ^**^*P* < 0.01; ^***^*P* < 0.001. Results from hierarchical linear modeling. Unstandardized regression coefficients are reported. Standard errors in parentheses.

^†^ Gender inequality in higher education was obtained and computed from the Current Population Survey (CPS; 2003-2020) reported by the U.S. Census Bureau.

**Table S31. Links between gender bias in cultural tightness (Wave 2) and gender inequality in entrepreneurship.**

| Variables | Gender inequality in number of startup firms | | Gender inequality in all firm ownership | |
| --- | --- | --- | --- | --- |
|  | Model 1 | Model 2 | Model 3 | Model 4 |
| Gender bias in cultural tightness | **0.02^***^ (0.01)** | **0.03^***^ (0.01)** | **0.02^*^ (0.01)** | **0.02^*^ (0.01)** |
| GDP per capita (log) |  | 0.08^*^ (0.03) |  | 0.10^**^ (0.04) |
| Gender imbalance in population (more men than women) |  | -0.58^**^ (0.18) |  | -0.13 (0.24) |
| State cultural tightness |  | 0.00 (0.00) |  | 0.00 (0.00) |
| Constant | 0.61^***^ (0.02) | -0.23 (0.35) | 0.30^***^ (0.02) | -0.86^*^ (0.41) |
| Year | 2014-2016 | 2014-2016 | 2002-2012 | 2002-2012 |
| *N* | 150 | 150 | 150 | 150 |

^+^*P* < 0.1; ^*^*P* < 0.05; ^**^*P* < 0.01; ^***^*P* < 0.001. Results from hierarchical linear modeling. Unstandardized regression coefficients are reported. Standard errors in parentheses.

^†^ Gender inequality in number of startup firms was obtained and computed from the Annual Survey of Entrepreneurs (ASE; 2014-2016), while gender inequality in all firm ownership was obtained and computed from the Survey of Business Owners (SBO; 2002-2012) from the U.S. Census Bureau.

**Table S32. Relationship between gender, age, and education level and gender bias in cultural tightness perceptions.**

| Variables | Model 1 |
| --- | --- |
| Gender | -0.08^***^ (0.01) |
| Age | 0.00 (0.00) |
| Education level | 0.01 (0.01) |
| Socioeconomic status | -0.01 (0.01) |
| State | Included |
| Constant | 4.13^***^ (0.05) |
| *N* | 15425 |

^+^*P* < 0.1; ^*^*P* < 0.05; ^**^*P* < 0.01; ^***^*P* < 0.001. Results from ordinary least squares regressions. Unstandardized regression coefficients are reported. Standard errors in parentheses.

**Table S33. Relationships between state characteristics and differences between sample and population demographics.**

| Variables | The difference in gender^†^ | The difference in age | The difference in education level |
| --- | --- | --- | --- |
| GDP per capita (log) | 0.03 (0.03) | 0.56 (1.98) | 0.09 (0.08) |
| Population (log) | -0.01 (0.01) | 0.98^*^ (0.37) | 0.00 (0.01) |
| Urbanization | 0.09^+^ (0.05) | 0.90 (2.97) | -0.04 (0.12) |
| Constant | -0.30 (0.37) | -24.76 (22.10) | -0.70 (0.86) |
| *N* | 50 | 50 | 50 |

^+^*P* < 0.1; ^*^*P* < 0.05; ^**^*P* < 0.01; ^***^*P* < 0.001. Results from ordinary least squares regressions. Unstandardized regression coefficients are reported. Standard errors in parentheses.

^†^ Differences in age, gender, and education level were computed by subtracting our sample’s average age, gender, and education level at the state level from those of the general population within the state. The average age, gender, and education level of each state from the U.S. Census Bureau (2020). GDP per capita was obtained from the Institute for Policy & Social Research, the University of Kansas (2020). Population and urbanization from the U.S. Census Bureau (2020).

**
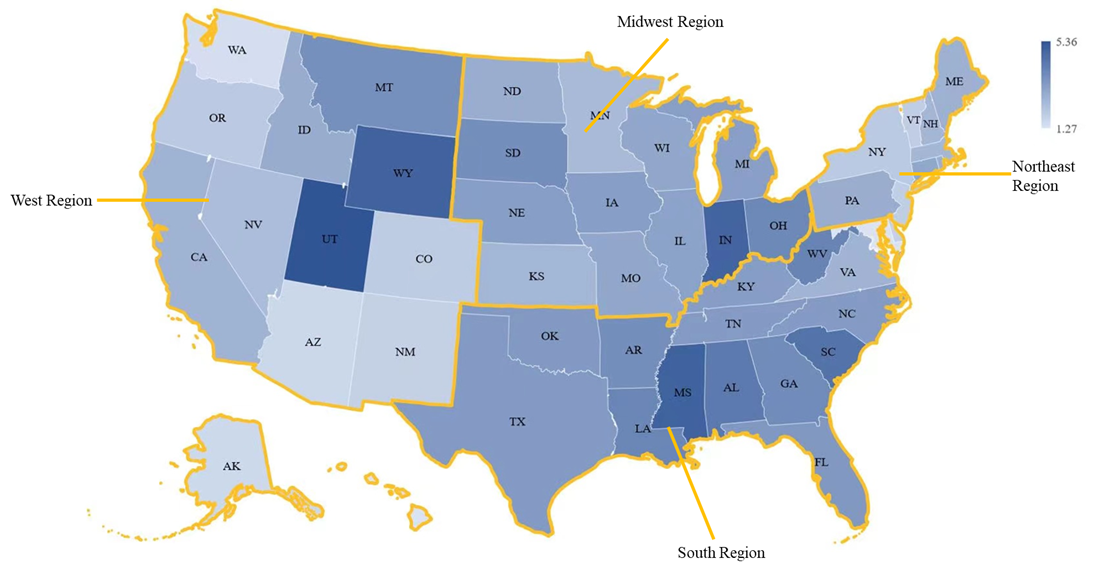
**

**Fig. S1. Gender bias in cultural tightness in the four U.S. census regions.**


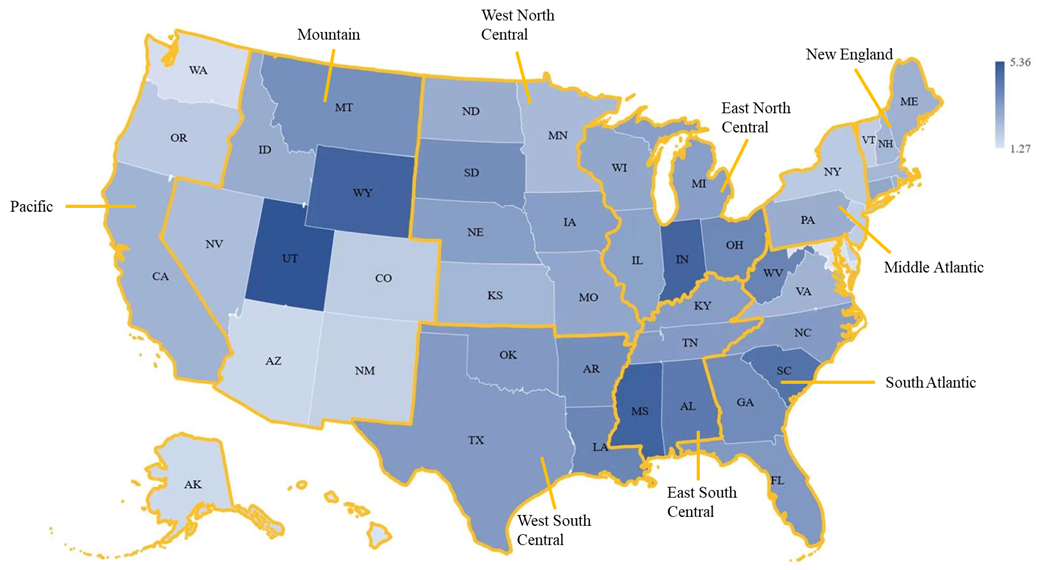


**Fig. S2. Gender bias in cultural tightness in the nine U.S. census regional divisions.**

**Supplementary Materials References**

1. V. Galasso *et al*., Gender differences in COVID-19 attitudes and behavior: Panel evidence from eight countries. Proc. Natl. Acad. Sci. U.S.A. 117, 27285-27291 (2020).
2. L. M. Giurge, A. V. Whillans, A. Yemiscigil, A multicountry perspective on gender differences in time use during COVID-19. *Proc. Natl. Acad. Sci. U.S.A.*, 10.1073/pnas.2018494118, in press.
3. B. L. Perry, B. Aronson, B. A. Pescosolido, Pandemic precarity: COVID-19 is exposing and exacerbating inequalities in the American heartland. *Proc. Natl. Acad. Sci. U.S.A.*, 10.1073/pnas.2020685118, in press.
4. T. K. Kumar, Multicollinearity in Regression Analysis. *Rev. Econ. Stat.* **57**, 365–366 (1975).
5. RepresentWomen, Gender Parity Index 2019 Report. <https://fairvote.app.box.com/s/zqj8hcwwnjy5ikhdu8ui67t2jbropxs5>. Accessed 29 January 2021.
6. WalletHub, WalletHub 2020’s Best & Worst States for Women’s Equality Report. <https://wallethub.com/edu/best-and-worst-states-for-women-equality/5835/>. Accessed 29 January 2021.
7. Human Rights Campaign Foundation and the Equality Federation Institute, Municipal Equality Index. <https://www.hrc.org/resources/municipal-equality-index> (2020). Accessed 29 January 2021.
8. L. Ruppanner, D. J. Maume, The state of domestic affairs: Housework, gender and state-level institutional logics. *Soc. Sci. Res*. **60**, 15–28 (2016).
9. J. Vandello, D. Cohen, Patterns of individualism and collectivism across the United States. *J. Pers. Soc. Psychol.* **77**, 279–292 (1999).
10. U.S. Centers for Disease Control and Prevention (CDC), Behavioral Risk Factor Surveillance System*.* [https://www.cdc.gov/brfss/annual_data/annual_data.htm. Accessed 29 January 2021](https://www.cdc.gov/brfss/annual_data/annual_data.htm.%20Accessed%2029%20January%202021).
11. U.S. Bureau of Economic Analysis, Annual Gross Domestic Product (GDP) by State*.* <https://apps.bea.gov/itable/iTable.cfm?ReqID=70&step=1>. Accessed 10 September 2020.
12. U.S. Census Bureau, State Population by Characteristics. <https://www.census.gov/data/tables/time-series/demo/popest/2010s-state-detail.html>. Accessed 6 January 2022.
13. U.S. Census Bureau, 2020 Census. <https://www.census.gov/newsroom/press-releases/2022/urban-rural-populations.html>. Accessed 10 January 2023.
14. Pew Research Center, Religious Landscape Study, Beliefs and practices by state. <https://www.pewforum.org/religious-landscape-study/> (2014). Accessed 03 November 2020.
15. Gallup, Gallup Daily Tracking, State by state. <https://news.gallup.com/poll/203747/mississippi-retains-standing-religious-state.aspx> (2016). Accessed 03 November 2020.
16. C. Haerpfer et al., Eds., *World Values Survey: Round Seven - Country-Pooled Datafile* (Madrid, Spain & Vienna, Austria, JD Systems Institute & WVSA Secretariat, 2020).
17. DDB Worldwide, DDB Life Style Survey Data, 1975-1998. <http://bowlingalone.com/?page_id=7>. Accessed 30 June 2020.
18. Public Religion Research Institute (PRRI), American Values Atlas, Research on LGBT. <http://ava.prri.org/#lgbt/2019/States/lgbtdis/m/US-VT> (2019). Accessed 29 January 2021.
19. Pew Research Center, Religious Landscape Study, Views about homosexuality by state. <https://www.pewforum.org/religious-landscape-study/compare/views-about-homosexuality/by/state/> (2014). Accessed 29 January 2021.
20. U.S. Bureau of Economic Analysis, Total Full-Time and Part-Time Employment by NAICS Industry*.* <https://apps.bea.gov/regional/downloadzip.cfm>. Accessed 10 September 2020.
21. National Center for Injury Prevention and Control of the Centers for Disease Control and Prevention, The National Intimate Partner and Sexual Violence Survey: 2010 Summary Report. <https://www.cdc.gov/violenceprevention/pdf/NISVS_Report2010-a.pdf>. (2021). Accessed 20 October 2022.
22. National Human Trafficking Hotline, <https://humantraffickinghotline.org/states>. Accessed 20 October 2022.
23. Biographical Directory of the United States Congress, 2019-2021. <https://bioguide.congress.gov/search>. Accessed 29 January 2021.
24. Institutional Shareholder Services (ISS). <https://www.issgovernance.com>. Accessed 17 March 2022.
25. Compustat. <http://wrds.wharton.upenn.edu>. Accessed 18 March 2022.
26. Center for American Women and Politics (CAWP), IWPR analysis of American Community Survey data, Integrated Public Use Microdata Series, Version 5.0. <https://statusofwomendata.org/explore-the-data/employment-and-earnings/additional-state-data/managerial-professional-jobs-by-race-ethnicity/>. Accessed 30 June 2020.
27. Center for American Women and Politics (CAWP), IWPR compilation of data. <https://statusofwomendata.org/explore-the-data/political-participation/#download-data> (2015). Accessed 30 June 2020.
28. Center for American Women and Politics (CAWP). <https://cawp.rutgers.edu/women-candidates-state-leg-historical-summary>. Accessed 30 June 2020.
29. U.S. Patent and Trademark Office, Office of the Chief Economist, PatentsView. [https://www.patentsview.org/](https://www.patentsview.org/download/). Accessed 4 January 2022.
30. U.S. Census Bureau, American Community Survey, ACS 1 Year Estimate-Sex by occupation for the full-time, year-round civilian employed population 16 years and over. <https://www.census.gov/programs-surveys/acs/>. Accessed 4 January 2022.
31. U.S. Census Bureau, Current Population Survey (CPS). <https://www.census.gov/programs-surveys/cps.html>. Accessed 6 January 2022.
32. U.S. Census Bureau, Annual Survey of Entrepreneurs, ASE-2014/2015/2016. <https://factfinder.census.gov/faces/nav/jsf/pages/download_center.xhtml>. Accessed 30 June 2020.
33. U.S. Census Bureau, Survey of Business Owners (SBO). <https://factfinder.census.gov/faces/nav/jsf/pages/download_center.xhtml>. Accessed 30 June 2020.

1. Since gender bias in cultural tightness by using data reported by women and men were highly correlated (*r*_[48]_ = 0.78, *P* < 0.001, *n* = 50), including both of them simultaneously to predict gender inequality in leadership and innovation in the regressions induces serious multicollinearity problems and may yield misleading even opposite results (4). Accordingly, we did not include them simultaneously to predict gender inequality in leadership and innovation in the regressions. [↑](#footnote-ref-1)
2. We also conducted additional analyses to test whether state GDP per capita, population, or urbanization were correlated with the discrepancies in representativeness. Results in Table S33 indicated that, while state urbanization was marginally and positively related to the difference in gender (*b* = 0.09, *SE* = 0.05, *P* = 0.077), and state population was positively related to the difference in age (*b* = 0.98, *SE* = 0.37, *P* = 0.011), state GDP per capita, population, and urbanization were not related to other differences in gender, age, and education level. [↑](#footnote-ref-2)
3. GDP per capita in 2020 were obtained from the Institute for Policy & Social Research, The University of Kansas, (https://ipsr.ku.edu/ksdata/ksah/business/, Accessed 24 March 2022), as BEA has not yet released its data. [↑](#footnote-ref-3)
4. This measure is comprised of five items: “When a mother works for pay, the children suffer,” “On the whole, men make better political leaders than women do,” “A university education is more important for a boy than for a girl,” “On the whole, men make better business executives than women do,” and “Being a housewife is just as fulfilling as working for pay.” All items are reversed coded. [↑](#footnote-ref-4)
5. This measure is comprised of five items: “The father should be the boss in the house,” “Men are naturally better leaders than women,” “Men are smarter than women,” “I think the women’s liberation movement is a good thing” (reversed), and “Woman’s place is in the home.” [↑](#footnote-ref-5)
6. Gender information of the board members were obtained from the ISS and the locations of company’s headquarters were obtained from the Compustat. [↑](#footnote-ref-6)
7. Nebraska is unicameral, i.e., only state senate. [↑](#footnote-ref-7)
8. Gender inequality in state governors = 1 if the number of women state governors = 0; gender inequality in state governors = 0 if the number of women state governors = 1. [↑](#footnote-ref-8)
9. For detailed descriptions of the methodology applied to identify the gender of patent inventors based on their names and a combination of data extracted from the PatentsView and other resources, please refer to USPTO office of the chief economist, “Progress and Potential: A profile of women inventors on U.S. patents” (2019; https://www.uspto.gov/sites/default/files/documents/Progress-and-Potential.pdf). [↑](#footnote-ref-9)
10. Data from the ACS estimate the number of full-time, year-round civilian employed population with 16 years and over in STEM occupations by gender. [↑](#footnote-ref-10)
